# Cross-correlated relaxation measurements under adiabatic sweeps: determination of local order in proteins

**DOI:** 10.1007/s10858-015-9994-8

**Published:** 2015-10-28

**Authors:** Pavel Kadeřávek, Sarina Grutsch, Nicola Salvi, Martin Tollinger, Lukáš Žídek, Geoffrey Bodenhausen, Fabien Ferrage

**Affiliations:** École Normale Supérieure - PSL Research University, Département de Chimie, 24 rue Lhomond, 75005 Paris, France; Sorbonne Universités UPMC Univ Paris 06, LBM, 4 place Jussieu, 75005 Paris, France; UMR 7203 LBM, CNRS, 75005 Paris, France; National Centre for Biomolecular Research, Faculty of Science, Masaryk University, Kamenice 5, 625 00 Brno, Czech Republic; Central European Institute of Technology, Masaryk University, Kamenice 5, 625 00 Brno, Czech Republic; Institut des Sciences et Ingénierie Chimiques, École polytechnique fédérale de Lausanne, 1015 Lausanne, Switzerland; Institute of Organic Chemistry, Center for Molecular Biosciences Innsbruck (CMBI), University of Innsbruck, Innrain 80/82, 6020 Innsbruck, Austria

**Keywords:** NMR, Protein dynamics, Adiabatic sweep, Cross-correlated cross relaxation

## Abstract

Adiabatically swept pulses were originally designed for the purpose of broadband spin inversion. Later, unexpected advantages of their utilization were also found in other applications, such as refocusing to excite spin echoes, studies of chemical exchange or fragment-based drug design. Here, we present new experiments to characterize fast (ps–ns) protein dynamics, which benefit from little-known properties of adiabatic pulses. We developed a strategy for measuring cross-correlated cross-relaxation (CCCR) rates during adiabatic pulses. This experiment provides a linear combination of longitudinal and transverse CCCR rates, which is offset-independent across a typical amide $^{15}\hbox {N}$ spectrum. The pulse sequence can be recast to provide accurate transverse CCCR rates weighted by the populations of exchanging states. Sensitivity can be improved in systems in slow exchange. Finally, the experiments can be easily modified to yield residue-specific correlation times. The average correlation time of motions can be determined with a single experiment while at least two different experiments had to be recorded until now.

**Electronic supplementary material:**

The online version of this article (doi:10.1007/s10858-015-9994-8) contains supplementary material, which is available to authorized users.

## Introduction

Protein dynamics are essential for most biological processes. The possibility of studying dynamics on various timescales at atomic resolution makes the analysis of NMR relaxation rates unique among other biophysical methods. In principle, motions of any arbitrary spin can be studied, but relaxation studies of $^{15}\hbox {N}{-}^1\hbox {H}$ spin pairs in peptide bonds have been popular since they offer a series of benefits (i) they can be easily introduced into proteins by $^{15}\hbox {N}$ labeling, (ii) the approximation of isolated spin pairs can be safely assumed to be fulfilled, provided all other protons are replaced by deuterons, (iii) they provide a residue-specific description of protein backbone dynamics, and finally (iv) the measurement techniques are quite robust (Korzhnev et al. 2001; Lakomek et al. 2012).

According to semi-classical NMR relaxation theory (Wangsness and Bloch 1953; Redfield 1965) all relaxation rates are determined by linear combinations of discrete values of the spectral density function $J(\omega )$ which describes the probability of finding motions at a given frequency. The determination of the values of the spectral density function and their interpretation is known as spectral density mapping (Peng and Wagner 1992a, b). A simplified variant is called reduced spectral density mapping (Ishima and Nagayama 1995a, b; Farrow et al. 1995). Later, it has been proposed to complement reduced spectral density mapping analysis by protocols that utilize CCCR rates (Kadeřávek et al. 2014; Kroenke et al. 1998). Robust symmetrical reconversion methods allow accurate measurement of transverse CCCR rates (Pelupessy et al. 2003), but the precision is significantly reduced in the presence of slow exchange when the precession frequency changes during the exchange process.

Relaxation under adiabatic pulses has been studied in the presence of protein-ligand interactions (Auer et al. 2010), slow motions in the $\upmu$s–ms regime (Mulder et al. 2001; Mangia et al. 2010; Auer et al. 2011) and in the presence of fast (ps–ns) dynamics (Konrat and Tollinger 1999). Here, we present measurements of CCCR rates under adiabatically swept pulses and show that deleterious effects of chemical exchange are significantly reduced.

In addition, we introduce variants of experiments that can directly provide values of the spectral density function at zero frequency *J*(0). These *J*(0) values provide interesting information about the timescales of the dominant motion in the ps-ns range. Local disorder in a protein can be readily identified by a decrease of *J*(0) values in flexible segments where the weights of rapid internal motions characterized by small correlation times increase. To the best of our knowledge, this is the first method that allows one to measure *J*(0) directly in a single experiment.

## Materials and methods

### Protein samples

Uniformly $^{15}\hbox {N}$ labeled and deuterated KID-binding domain (KIX) [residues 586–672 of human CREB-binding protein (CBP)] was expressed using *Escherichia coli* strain BL21 (DE3). Cells were grown in minimal $\hbox {D}_{2}\hbox {O}$ medium containing $^{15}\hbox {N}$ labeled ammonium chloride as sole nitrogen source. KIX was purified as described elsewhere (Tollinger et al. 2006), and the exchangeable deuterons were back-exchanged in $\hbox {H}_{2}\hbox {O}$ buffer. The KIX sample contained 1 mM protein in 50 mM potassium phosphate buffer, pH 5.8, and 25 mM NaCl before adding 10 % $\hbox {D}_{2}\hbox {O}$.

Human ubiquitin was purchased from VLI. The sample was prepared by dissolution of lyophilized ubiquitin in 50 mM acetate buffer (pH 4.6 before 10 % of $\hbox {D}_{2}\hbox {O}$ was added to the solution). The final protein concentration was 0.67 mM.

### NMR experiments

The experiments were performed in magnetic fields $B_0=11.74$ and 18.79 T on Bruker Avance spectrometers ($^{1}\hbox {H}$ Larmor frequencies 500 and 800 MHz, respectively). Both spectrometers were equipped with TCI cryoprobes. The KIX domain was studied at $20\,^{\circ }\hbox {C}$ at 500 MHz. Experiments with ubiquitin were carried at $5\,^{\circ }\hbox {C}$ (500 MHz) and $30\,^{\circ }\hbox {C}$ (500 and 800 MHz). The temperature was calibrated by measuring the difference between $^{1}\hbox {H}$ chemical shifts of a 4 % solution of methanol in deuterated methanol.

**Fig. 1:**
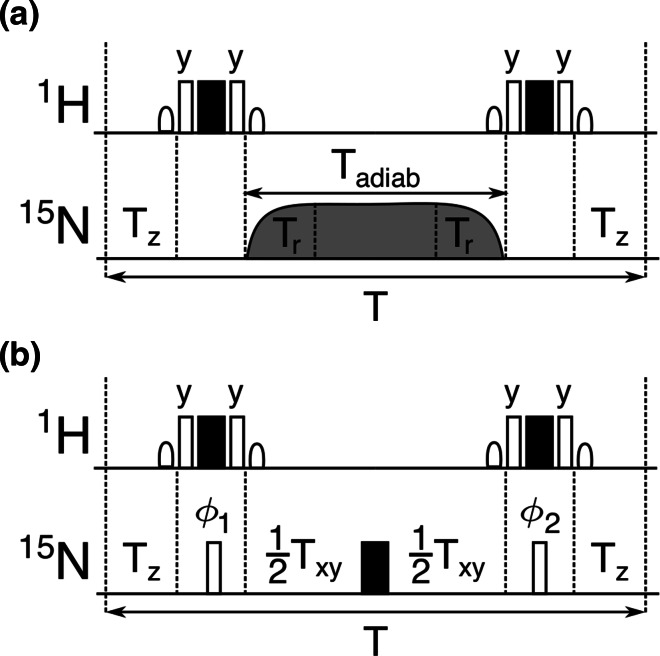
Variants of relaxation intervals **a** scheme 1—with adiabatically swept pulse and **b** scheme 2—with single spin echo. Rectangular $\pi /2$ and $\pi$ pulses are denoted as *open and filled boxes*. All pulses are applied with phase along *x* axis unless indicated. The phases $\phi _1$ and $\phi _2$ follow cycles {y,$-$y} and {y,y,$-$y,$-$y}, respectively. The long *grey shaped* pulse represents the adiabatic inversion pulse of length $T_{\mathrm{adiab}}$ where the amplitude is modulated by two ramps of length $T_{\mathrm{r}}$ each. Sinc type selective pulses applied to the water resonance are represented by *semi-elliptic symbols*. The carrier frequency for all hard pulses was placed in the centre of the amide region while all flip back pulses were applied on-resonance with respect to the water signal. The amplitudes of the selective pulses preceding and following the composite pulses were calibrated separately

Symmetrical reconversion experiments (Pelupessy et al. 2003, 2007) were used to measure the transverse ($\eta _{xy}$) and longitudinal ($\eta _z$) cross-relaxation rates due to cross-correlated fluctuations of the $^{15}\hbox {N}$ chemical shielding anisotropy (CSA) and the $^{15}\hbox {N}$–$^{1}\hbox {H}$ dipole–dipole interaction in ubiquitin. The transverse cross-relaxation rate $\eta _{xy}$ was measured with relaxation delays $T=50$ and 70 ms, the longitudinal cross-relaxation rate $\eta _z$ with $T=100$, 175, and 250 ms. Experiments with adiabatic sweeps employed $T_{\mathrm{adiab}}=60$ and 80 ms long apodized chirp pulses (Böhlen et al. 1990). The maximum amplitude of all chirp pulses was applied for 60 % of the duration of the pulses. The chirp pulses were divided into 10,000 discrete steps to cover a sweep width of 10 kHz. The central part of the relaxation blocks ($T_{xy}$ in Fig. 1) of our experiments with a single $^{15}\hbox {N}$ echo and non-selective $^{15}\hbox {N}$ pulses was 50 or 70 ms long.

The transverse CCCR rates $\eta _{xy}$ of KIX were determined by a single experiment with a relaxation delay $T =38.768\,\hbox {ms}$ and by our experiment with a chirp pulse of a length $T_{\mathrm{adiab}}=80\,\hbox {ms}$ (all other parameters were as for the experiments performed on ubiquitin).

The longitudinal relaxation rates $R_1(^{15}\hbox {N})$ in ubiquitin were measured (Korzhnev et al. 2001) with relaxation delays $T=140$ (twice), 220, 380, 500, 540 (twice), and 660 ms at 500 MHz and with $T=20$, 60, 140 (twice), 220, 380, 500, 540 (twice), and 660 ms at 800 MHz.

The $$^{15}\hbox {N}$$–$$^{1}\hbox {H}$$ steady-state nuclear Overhauser effect $$\sigma \{{}^{1}\hbox {H}\}$$ (ssNOE) in ubiquitin at 800 MHz and 30$$\,^{\circ }$$C was determined from the ratio between the intensities of spectra acquired with proton saturation achieved by a 8 s long repetition of a segment consisting of a $$180\,^\circ$$$${^1}\hbox {H}$$ pulse and a 10.87 ms delay and the intensities in reference spectra acquired with a 17 s interscan delay (Ferrage et al. 2009). The steady-state and reference spectra were measured in interleaved fashion. The same experiments were performed at 500 MHz and $$5\,^{\circ }\hbox {C}$$, but the duration of the proton saturation period was 6 s and the interscan delay in the reference experiments was 15 s.

The transverse relaxation rates $R_2(^{15}\hbox {N})$ of ubiquitin were determined from the relaxation rates $R_1$ and $R_{1\rho }$ (Mulder et al. 2001):

1 $$\begin{aligned} R_2=\frac{R_{1\rho }-R_1\cos ^2\theta }{\sin ^2\theta } \end{aligned}$$

where $\theta = \text {atan}(\omega _1/\varOmega )$ is the tilt angle of the effective field during the spin-lock irradiation, $\varOmega$ being the offset between the resonance frequency and the carrier frequency and $\omega _1$ the rf amplitude. The carrier frequency during the spin-lock was set in the middle of the amide region for durations $T=0, 20, 40$ (twice), 60, 80, 100, 120, and 140 (twice) ms.

The rf amplitudes for spin locking and chirp pulses were calibrated using a comparison of residual and true $J{(^{15}\mathrm {N}{-}{^1}\mathrm {H}^{\mathrm{N}})}$ coupling constants in spectra acquired with continuous-wave decoupling (Palmer et al. 2001). The calibrated rf amplitudes of chirp pulses were $2303.8 \pm 1.1, 2343.8 \pm 0.7, 2278.2 \pm 1.1,$ and $2306.1 \pm 3.0$ Hz for measurements of ubiquitin at 800 MHz ($30\,^{\circ }\hbox {C}$), 500 MHz ($30\,^{\circ }\hbox {C}$), 500 MHz ($5\,^{\circ }\hbox {C}$), and KIX domain, respectively. The calibrated rf amplitudes used for spin locking in $\hbox {R}_{1\rho }$ experiments were $1617.2 \pm 0.5,$ and $1040.6 \pm 0.5$ Hz for experiments at 800 MHz ($30\,^{\circ }\hbox {C}$), and 500 MHz ($5\,^{\circ }\hbox {C}$), respectively. Note that the errors were evaluated as asymptotic standard errors of the fit and are likely underestimated but this has no consequence on the analysis.

A compensation block was inserted to ensure uniform sample heating in measurements of $R_1$, $R_{1\rho }$, $\eta _{xy}$ and $\eta _z$.

All spectra were processed with NMRPipe (Delaglio et al. 1995) and analyzed with Sparky (Goddard and Kneller 2006). The $R_1$ and $R_{1\rho }$ relaxation rates were obtained from fits of the signal decays to mono-exponential functions using the program Octave 3.2.4 (Eaton et al. 2009).

The CCCR rates $\eta _{xy}$, $\eta _z$, and their linear combinations $\eta _{\mathrm{ave}}$ were obtained from a fit of the observable *Y*(*T*) defined as:

2 $$\begin{aligned} Y(T)=\sqrt{\frac{I_{P,Q}(T)I_{Q,P}(T)}{I_{P,P}(T)I_{Q,Q}(T)}} \end{aligned}$$

where $I_{P,Q}(T)$ denotes a peak intensity in spectra derived from operator terms *P* and *Q* selected before and after the relaxation period *T* in the manner of symmetrical reconversion. *P* and *Q* stand for $N_z$ and $2N_zH_z$ operators, respectively. The rates $\eta _{xy}$ and $\eta _{\mathrm{ave}}$ were determined from a linearized equation:

3 $$\begin{aligned} \mathrm {atanh} \left( Y(T) \right) \simeq \eta _i T, \end{aligned}$$

with $\eta _i=\eta _{xy}$ or $\eta _{\mathrm{ave}}$. $\eta _z$ was determined from [Eq. 7 in Pelupessy et al. (2007)]

4 $$\begin{aligned} \mathrm {atanh} \left( Y^{-1}(T) \right) =\eta _z T \left( 1 + \frac{\mu ^2 T^2}{24}\right) \end{aligned}$$

where $\mu$ is a correction factor determined from a simultaneous fit of [Eq. 8 in Pelupessy et al. (2007)]:

5 $$\begin{aligned} \frac{I_{P,P}(T)}{I_{Q,Q}(T)}= \frac{\lambda \exp (-\mu T)}{6+\eta _z^2 \mu T^3} \end{aligned}$$

where $\lambda$ is a fitted pre-exponential factor.

The errors of the peak intensities were estimated based on random noise in the spectra and the uncertainties of the relaxation rates were estimated from 2000 independent fits of the relaxation rates while the peaks intensities were varied according to their estimated errors assuming a normal error distribution.

The values of spectral density functions $J(\omega )$ were calculated using the reduced spectral density mapping approach, with the internuclear distance $r_{\mathrm{N{-}H}}$ = 1.02 Å, the anisotropy of the nitrogen chemical shielding tensor $\varDelta \sigma = \sigma _{\parallel } - \sigma _{\perp } = -170\,\hbox {ppm}$ and the angle between the unique axis of the CSA tensor and the N–H bond to $20.6\,^{\circ }$. The choice of the parameters was justified by a comparison of the spectral density values at the $^{15}\hbox {N}$ frequency obtained from the CCCR rates and the reduced spectral density mapping (Kadeřávek et al. 2014).

Simulations of the evolutions of the spin system (Levitt and Bari 1992; Ghose 2000) during our pulse sequence were performed with Matlab R2014a [MATLAB] using Spinach 1.5.2440 (Hogben et al. 2011). The full basis set was used with Redfield relaxation theory (Wangsness and Bloch 1953; Redfield 1965) using the secular approximation. Simulations for a two-site exchange between states on different time scales were performed by a script written in Mathematica 9 (Wolfram Research Inc. 2012).

## Theory

### Cross-correlated cross-relaxation

The relaxation of an isolated $^{1}\hbox {H}{-}^{15}\hbox {N}$ spin pair can be described by the semi-classical relaxation theory (Wangsness and Bloch 1953; Redfield 1965). Cross-correlation between the $^{15}\hbox {N}$ chemical shielding anisotropy (CSA) and the dipole–dipole interaction with the $\hbox {H}^{\mathrm{N}}$ proton is responsible for cross-relaxation pathways between the in-phase and anti-phase $^{15}\hbox {N}$ coherences (the transverse CCCR rate $\eta _{xy}$) and between the polarization $N_z$ and the two-spin order $2N_zH_z$ (the longitudinal CCCR rate $\eta _z$). The CCCR rates depend on the spectral density function:

6 $$\begin{aligned} \eta _{xy} & = C_{cd}\left( 8J(0)+6J(\omega _{\mathrm{N}})\right) , \end{aligned}$$

7 $$\begin{aligned} \eta _z & = 12C_{cd}J(\omega _{\mathrm{N}}), \end{aligned}$$

where $\omega _{\mathrm{N}}$ is the nitrogen Larmor frequency, $C_{cd} = (3\cos ^2\varphi -1)\gamma ^2_{\mathrm{N}}\gamma _{\mathrm{H}} B_0 \varDelta \sigma \mu _0 \hbar r^{-3}_{\mathrm{N{-}H}} / 96\pi$, $\gamma _{\mathrm{H}}$ and $\gamma _{\mathrm{N}}$ are the gyromagnetic ratios of $^{1}\hbox {H}$ and $^{15}\hbox {N}$, respectively, $r_{\mathrm{N{-}H}}$ is the H–N internuclear distance, $\mu _0$ is the permeability of vacuum, $\hbar$ is Planck’s constant divided by $2\pi$, $\varDelta \sigma$ is the anisotropy of the $^{15}\hbox {N}$ chemical shielding tensor, $\varphi$ is the angle between the H–N bond and the symmetry axis of the $^{15}\hbox {N}$ chemical shielding tensor, and $B_0$ is the external magnetic field. The spectral density function is described as a sum of *k* Lorentzian functions $J(\omega ) = (2/5)\sum _{i=0}^{k-1} a_i \tau _i / (1+(\omega \tau _i)^2)$, where $\tau _i$ are the effective correlation times and $a_i$ are weighting constants. Hence, $J(0) = (2/5)\tau _0$ for motions with a correlation function described by a mono-exponential decay characterized by a single correlation time $\tau _0$, and $J(0) = (2/5)\sum _{i=0}^{k-1} a_i \tau _i$ for more general cases where statistically independent motions participate. Within the frame of the popular model-free approach (Lipari and Szabo 1982a, b; Halle et al. 1981; Halle 2009) a local order parameter is defined as $S^2 = a_0$, where the index $i = 0$ refers to the overall tumbling of the molecule in the solution with a correlation time $\tau _0$ and $\tau _1 = (\tau _0^{-1} + \tau _f^{-1})^{-1}$, where $\tau _f$ is the timescale of the fast internal motions.

### Adiabatic sweeps

The adiabatic fast passage pulses (Abragam 1961; Böhlen and Bodenhausen 1993; Kupče and Freeman 1995) used in the relaxation period (Fig. 1) are phase-modulated pulses. The time dependence of the phase effectively sweeps the resonance frequency of the pulse in linear fashion over a defined range. The time dependence of the carrier frequency for the chirp pulses used in this study is:

8 $$\begin{aligned} \varOmega (t)= 2 \pi \left( \frac{t}{T_{\mathrm{adiab}}}-0.5\right) W \end{aligned}$$

where $T_{\mathrm{adiab}}$ is the total pulse duration and *W* is the sweep width in Hz. The amplitude of the chirp pulse starts from zero and smoothly increases like the first quarter of the period of a sine function during a ramping interval $0<t<T_{\mathrm{r}}$:

9 $$\begin{aligned} \omega _1(t)= 2 \pi B_1 \sin \frac{ \pi t}{2 T_{\mathrm{r}}} \end{aligned}$$

where $B_1$ is the maximum of rf amplitude in Hz, and $T_{\mathrm{r}}$ is the length of the ramp. At time $(T_{\mathrm{adiab}} - T_{\mathrm{r}})$ the amplitude of the rf pulse is gradually decreased according to equation:

10 $$\begin{aligned} \omega _1(t)= 2 \pi B_1 \sin \frac{ \pi (t-T_{\mathrm{adiab}}+2T_{\mathrm{r}})}{2 T_{\mathrm{r}}} \end{aligned}$$

The amplitude is constant $\omega _1(t) = 2 \pi B_1$ for $t\in [T_{\mathrm{r}},T_{\mathrm{adiab}}-T_{\mathrm{r}}]$. In order to evaluate the effective relaxation of a $^{15}\hbox {N}$–$^{1}\hbox {H}$ spin pair during the chirp pulse, average Liouvillian theory can be applied (Levitt and Bari 1992; Ghose 2000) as average Hamiltonian theory (Valentine et al. 2007). The relaxation can be described in a spin operator subspace $\{N_z, 2N_zH_z, N_x, 2N_xH_z\}$ in the instantaneous rotating frame (assuming that the chirp pulse was applied along the x-axis of the accelerating rotating frame). The zero-order average Liouvillian over the whole length of the pulse is given by:

11 $$\begin{aligned} \overline{\mathbf{L}}= \frac{1}{T_{\mathrm{adiab}}} \int \limits _0^{T_{\mathrm{adiab}}} \mathbf{L}'(t) \text {d}t. \end{aligned}$$

$\mathbf{L}'(t)$ is the Liouvillian at a time *t* which can be calculated as:

12 $$\begin{aligned} \mathbf{L}'(t)= \mathbf{U}^{-1}(t) \mathbf{L} \mathbf{U}(t), \end{aligned}$$

where $\mathbf{L}$ is the Liouvillian of the initial state with the following matrix representation in the basis $\{N_z, 2N_zH_z, N_x, 2N_xH_z\}$:

13 $$\begin{aligned} \mathbf{L}=\left( \begin{array}{cccc} R_1 &{}\quad \eta _z &{}\quad 0 &{}\quad 0 \\ \eta _z &{}\quad R'_1 &{}\quad 0 &{}\quad 0 \\ 0 &{}\quad 0 &{}\quad R_2 &{}\quad \eta _{xy} \\ 0 &{}\quad 0 &{}\quad \eta _{xy}&{}\quad R'_2 \\ \end{array} \right) \end{aligned}$$

where $R_1$ is the longitudinal $^{15}\hbox {N}$ relaxation rate, $R'_1$ is the relaxation rate of the two spin order and $R_2$ and $R'_2$ are the transverse relaxation rates of the in-phase and anti-phase $^{15}\hbox {N}$ coherences, respectively. $\eta _z$ and $\eta _{xy}$ are the longitudinal and transverse CCCR rates. $\mathbf{U}(t)$ is the transformation into the time-dependent tilted frame.

14 $$\begin{aligned} \mathbf{U}(t)=\left( \begin{array}{cccc} c &{}\quad 0 &{}\quad -s &{}\quad 0 \\ 0 &{}\quad c &{}\quad 0 &{}\quad -s \\ s &{}\quad 0 &{}\quad c &{}\quad 0 \\ 0 &{}\quad s &{}\quad 0 &{}\quad c \\ \end{array}\right) , \end{aligned}$$

where *c* and *s* stand for $\cos (\theta (t))$ and $\sin (\theta (t))$, respectively. The angle $\theta (t)$ can be calculated with the knowledge of the time-dependence of the offset and the amplitude (Eqs. 8–10)

15 $$\begin{aligned} \cos \theta (t)= & {} \frac{\varOmega (t)}{\sqrt{\varOmega ^2(t)+\omega ^2_1(t)}} \end{aligned}$$

16 $$\begin{aligned} \sin \theta (t)= & {} \frac{\omega _1(t)}{\sqrt{\varOmega ^2(t)+\omega ^2_1(t)}} \end{aligned}$$

Eqs. 12, 13, and 14 provide a Liouvillian in the transformed base $\{N_{{z^{\prime }}}(t), 2N_{{z^{\prime }}}H_{{z}}(t), N_{{x^{\prime }}}(t), 2N_{{x^{\prime }}}H_{{z}}(t)\}$:

17 $$\begin{aligned} \mathbf{L}'(t)=\left( \begin{array}{cccc} R_1 c^2 + R_2 s^2 &{} \quad \eta _z c^2 + \eta _{xy} s^2 &{} \quad (R_2-R_1) cs &{} \quad (\eta _{xy}-\eta _z) cs \\ \eta _z c^2 + \eta _{xy} s^2 &{} \quad R'_1 c^2 + R'_2 s^2 &{} \quad (\eta _{xy}-\eta _z) cs &{} \quad (R'_2-R'_1) cs \\ (R_2-R_1)cs &{} \quad (\eta _{xy}-\eta _z) cs &{} \quad R_2 c^2 + R_1 s^2 &{} \quad \eta _{xy} c^2 + \eta _z s^2 \\ (\eta _{xy}-\eta _z)cs &{} \quad (R'_2-R'_1) cs &{} \quad \eta _{xy} c^2 + \eta _z s^2 &{} \quad R'_2 c^2 + R'_1 s^2 \\ \end{array} \right) . \end{aligned}$$

The integral of $\mathbf{L}'(t)$ over the interval $[0,T_{\mathrm{adiab}}]$ gives the average Liouvillian. Note that the integral of the function $\cos (\theta (t))\sin (\theta (t))$ over the integration interval $[0,T_{\mathrm{adiab}}]$ vanishes. We introduce the parameter $\alpha$:

18 $$\begin{aligned} \alpha = \frac{1}{T_{\mathrm{adiab}}} \int \limits _0^{T_{\mathrm{adiab}}} \sin ^2\left( \theta (t)\right) \text {d}t \end{aligned}$$

which can be evaluated numerically. The averaged Liouvillian has the form:

19 $$\begin{aligned} \overline{\mathbf{L}}=\left( \begin{array}{cccc} R_1 (1-\alpha ) + R_2 \alpha &{} \quad \eta _z (1-\alpha ) + \eta _{xy} \alpha &{} \quad 0 &{} \quad 0 \\ \eta _z (1-\alpha ) + \eta _{xy} \alpha &{} \quad R'_1 (1-\alpha ) + R'_2 \alpha &{} \quad 0 &{} \quad 0 \\ 0 &{} \quad 0 &{} \quad R_2 (1-\alpha ) + R_1 \alpha &{} \quad \eta _{xy} (1-\alpha ) + \eta _z \alpha \\ 0 &{} \quad 0 &{} \quad \eta _{xy} (1-\alpha ) + \eta _z \alpha &{} \quad R'_2 (1-\alpha ) + R'_1 \alpha \\ \end{array} \right) \end{aligned}$$

We provide a list of $\alpha$ parameters calculated for various sets $\{B_1, W, T_{\mathrm{r}}/T_{\mathrm{adiab}}\}$ in the supplementary material (Tab. S1). It should be noted that the calculated $\alpha$ parameters and the zero-order averaged Liouvillian presented in Eq. 19 correspond to $^{15}\hbox {N}$ with a resonance frequency in the centre of the adiabatic sweep. For all other cases a constant offset must be added to $\varOmega (t)$ in Eq. 8. Consequently, the proportion of $\eta _{xy}$ and $\eta _z$ contributions to the average cross-relaxation rates depends on the offset. In addition, the integral of the product $\cos (\theta (t))\sin (\theta (t))$ does not vanish, so all terms in the matrix in Eq. 19 are non-zero a priori. However, errors are limited under the usual conditions (see below).

### Principles of the new experiments

The experiments that are presented in this paper combine relaxation during an adiabatic pulse with the inversion of proton magnetization and additional relaxation delays. The complete relaxation block (scheme 1) is shown in Fig 1a. It starts with a longitudinal relaxation delay $T_z$ followed by an adiabatic pulse of a length $T_{\mathrm{adiab}}$ flanked by two proton inversion pulses and terminated by a second longitudinal relaxation delay $T_z$. The averaged Liouvillian can be easily calculated as a weighted sum of three Liouvillians representing relaxation during the three delays. The Liouvillian during the first free relaxation period $T_z$ is equal to $\mathbf{L}$ in Eq. 13. The effect of the proton inversion pulses is described by a unitary transformation $\mathbf{U}_{\mathrm{H}}$ in the same basis as Eq. 13:

20 $$\begin{aligned} \mathbf{U}_{\mathrm{H}} =\left( \begin{array}{cccc} 1 &{}\quad 0 &{}\quad 0 &{}\quad 0 \\ 0 &{}\quad -1 &{}\quad 0 &{}\quad 0 \\ 0 &{}\quad 0 &{}\quad 1 &{}\quad 0 \\ 0 &{}\quad 0 &{}\quad 0 &{}\quad -1 \\ \end{array}\right) \end{aligned}$$

The averaged Liouvillian over the relaxation delay $T\simeq (2T_z+T_{\mathrm{adiab}})$ where the duration of pulses can be neglected is:

21 $$\begin{aligned} \overline{\mathbf{L}}_1=\frac{1}{T}\left( 2\mathbf{L}T_z+T_{\mathrm{adiab}}\mathbf{U}^{-1}_{\mathrm{H}}\overline{\mathbf{L}}\mathbf{U}_{\mathrm{H}}\right) \end{aligned}$$

where $\overline{\mathbf{L}}$ is defined in Eq. 19.

A similar experiment (scheme 2) can be proposed if the adiabatic sweep is replaced by a $$^{15}\hbox {N}$$ refocusing $$\pi$$ pulse flanked by two delays $$T_{xy}/2$$ and two non-selective $$^{15}\hbox {N}$$$$\pi /2$$ pulses (Fig 1b). The transformation corresponding to the $$^{15}\hbox {N}$$ refocusing pulse is described by a simple multiplication by the factor $$-1$$ which has no effect on the Liouvillian. $$\mathbf{U}_{\mathrm{N}}$$ represents the transformation of the Liouvillian due to a $$^{15}\hbox {N}$$$$\pi /2$$ pulse applied along y-axis:

22 $$\begin{aligned} \mathbf{U}_{\mathrm{N}} = \left( \begin{array}{cccc} 0 &{}\quad 0 &{}\quad -1 &{}\quad 0 \\ 0 &{}\quad 0 &{}\quad 0 &{}\quad -1 \\ 1 &{}\quad 0 &{}\quad 0 &{}\quad 0 \\ 0 &{}\quad 1 &{}\quad 0 &{}\quad 0 \\ \end{array} \right) \end{aligned}$$

The averaged Liouvillian over the relaxation interval in scheme 2 is thus given by:

23 $$\begin{aligned} \overline{\mathbf{L}}_2 =\frac{1}{2T_z+T_{xy}}\left( 2\mathbf{L}T_z+T_{xy}\mathbf{U}^{-1}_{\mathrm{N}}\mathbf{U}^{-1}_{\mathrm{H}}\mathbf{L}\mathbf{U}_{\mathrm{H}}\mathbf{U}_{\mathrm{N}}\right) \end{aligned}$$

which is equal to $\overline{\mathbf{L}}_1$ of Eq. 21 if $\alpha = 1$ and $T_{xy} = T_{\mathrm{adiab}}$.

## Results and discussion

### Choice of the relaxation intervals

The cross-relaxation measurements use symmetrical reconversion (Pelupessy et al. 2003): $N_z$ or $2N_zH_z$ terms are prepared prior to the relaxation period *T* and either $N_z$ or $2N_zH_z$ are selected at the end of *T*. All four relaxation pathways are recorded. An analysis of relaxation during our experiments shows that the observable *Y*(*T*) (Eqs. 2, 3) results from a linear combination of transverse ($\eta _{xy}$) and longitudinal ($\eta _z$) CCCR rates in the zeroth order average Liouvillian. The observed averaged CCCR rate $\eta _{\mathrm{ave}}$ determined by our experiments is:

24 $$\begin{aligned} \eta _{\mathrm{ave}}=\frac{2T_z\eta _z-T_{\mathrm{centr}}\left( \alpha \eta _{xy}+ \left( 1-\alpha \right) \eta _z\right) }{T} \end{aligned}$$

where *T* is the total relaxation period and $T_{\mathrm{centr}} = T_{\mathrm{adiab}}$ or $T_{\mathrm{centr}} = T_{xy}$ for scheme 1 or 2 as defined in Fig 1. The parameter $\alpha$ defines the proportion of time that the $^{15}\hbox {N}$ magnetization spends in the transverse plane during the $T_{\mathrm{centr}}$ period. $\alpha = 1$ for all N–H$^{\mathrm{N}}$ groups for scheme 2 (called single echo experiment in this article) provided the pulses are sufficiently strong. $0<\alpha <1$ when an adiabatically swept pulse is used (scheme 1, called adiabatic experiment in this article). If the $T_z$ and $T_{\mathrm{centr}}$ periods are chosen to fulfill $T_z = 0.5(1-\alpha )T_{\mathrm{centr}}$, the right hand side of Eq. 24 does not depend on $\eta _z$, so that $\eta _{xy}$ can readily be determined:

25 $$\begin{aligned} \eta _{\mathrm{ave}}=\eta ^{xy}_{\mathrm{ave}}=\eta _{xy}\frac{\alpha }{\alpha -2} \end{aligned}$$

On the other hand, if $T_z = 0.5 (1-0.5\alpha )T_{\mathrm{centr}}$ the right hand side of Eq. 24 does not depend on $J(\omega _{\mathrm{N}})$. Then, *J*(0) can be calculated directly from the analysis of this single experiment. Equations 6, 7 and 24 give in this case:

26 $$\begin{aligned} \eta _{\mathrm{ave}}=\eta ^{J}_{\mathrm{ave}}=J(0)\frac{16\alpha C_{cd}}{\alpha -4} \end{aligned}$$

With $0<\alpha <1$, the magnitude of both $\eta ^{xy}_{\mathrm{ave}}$ and $\eta ^{J}_{\mathrm{ave}}$ increase with $\alpha$. The $\alpha$ parameter should be maximized to increase the precision of the $\eta _{xy}$ and *J*(0) determination.

Finally, if the flanking $T_z$ periods are omitted in the adiabatic experiment (scheme 1 in Fig. 1) and if the relaxation delay is reduced to the adiabatic pulse, a linear combination of longitudinal and transverse CCCR rates is obtained

27 $$\begin{aligned} \eta _{\mathrm{ave}}=\eta ^{xyz}_{\mathrm{ave}}=\alpha \eta _{xy}+\left( 1-\alpha \right) \eta _z \end{aligned}$$

Both $\eta _z$ and $\eta _{xy}$ can be determined from at least two experiments with different $\alpha$ parameters. In principle, this is similar to the standard experiments for the separate determination of $\eta _{xy}$ and $\eta _z$. But it might be more advantageous if an exchange significantly contributes to relaxation of the $^{15}\hbox {N}$ magnetization, as discussed below.

### Systematic errors

The $\alpha$ parameter weakly depends on the rf field strength $B_1$, so that the method is robust with respect to a possible miscalibration of the amplitude of the adiabatic pulse. The relative errors are (see Appendix):

28 $$\begin{aligned} \frac{ \eta _{xy}-\eta ^{\prime }_{xy}}{\eta _{xy}}= & {} \left( 1-\frac{\alpha }{\alpha ^{\prime }}\right) F_{\eta } \end{aligned}$$

29 $$\begin{aligned} \frac{ J(0)-J^{\prime }(0)}{J(0)}= & {} \left( 1-\frac{\alpha }{\alpha ^{\prime }}\right) F_J \end{aligned}$$

where $J^{\prime }(0)$ and $\eta ^{\prime }_{xy}$ are obtained from the analysis of experimental data based on an $\alpha ^{\prime }$ parameter set to an erroneous value, while the correct value was $\alpha$. The factors $F_{\eta }$ and $F_{J}$ depend only on the rotational diffusion of the molecule, its internal motions and the magnetic field of the spectrometer:

30 $$\begin{aligned} F_{\eta }= & {} \frac{4J(0)-3J(\omega _{\mathrm{N}})}{4J(0)+3J(\omega _{\mathrm{N}})} \end{aligned}$$

31 $$\begin{aligned} F_J= & {} \frac{4J(0)-3J(\omega _{\mathrm{N}})}{4J(0)} \end{aligned}$$

**Fig. 2:**
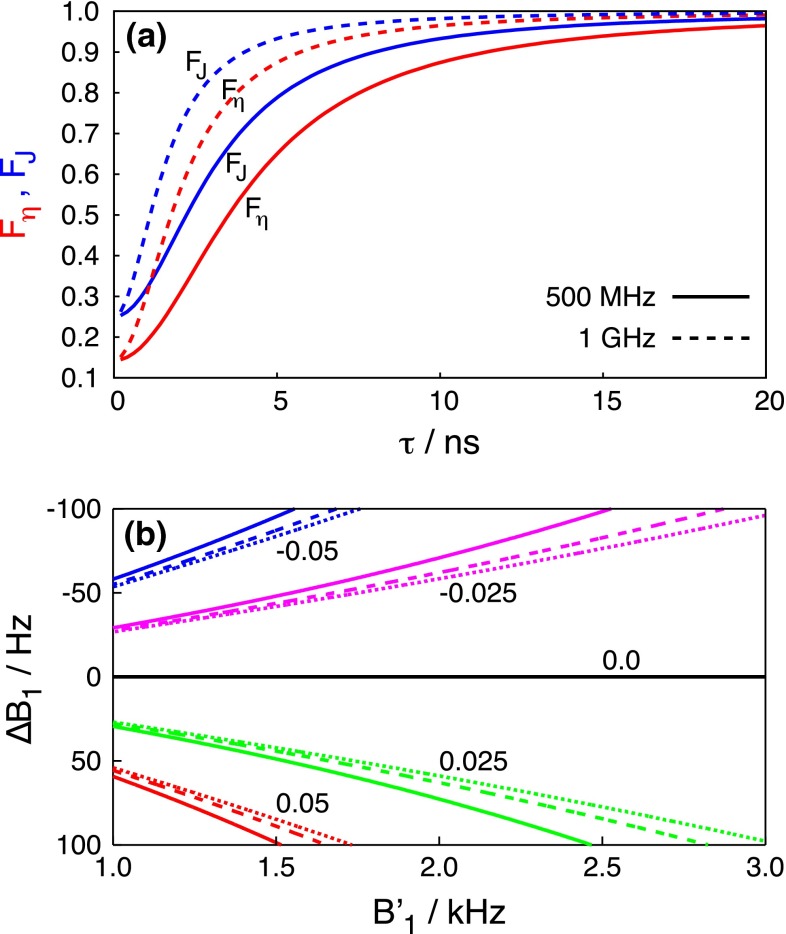
Contributions to the relative errors of *J*(0) and $\eta _{xy}$ caused by miscalibration of the amplitude of the chirp pulse. **a** A dependence of the factors $F_{\eta }$ and $F_J$ on the correlation time of rotational diffusion. $F_{\eta }$ is related to the error of $\eta _{xy}$ (*red lines*) and $F_J$ is related to the error of J(0) (*blue lines*). The *solid and dashed lines* correspond to measurements at 500 MHz and 1 GHz, respectively. **b** A *contour plot* of the dependence of the error factor $(1-\alpha /\alpha ^{\prime })$ on the amplitude of the adiabatic pulse $B^{\prime }_1$ and its deviation $\varDelta B_1 = B_1-B^{\prime }_1$ from the nominal value $B_1$. The *blue*, *magenta*, *black*, *green*, and *red lines* represent contours of the function $(1-\alpha /\alpha ^{\prime })$ at levels $-0.05, -0.025, 0, 0.025$, and 0.05, respectively. The *solid, dashed, and dotted lines* show contours for chirp pulses with 10, 15, and 20 kHz sweep widths, respectively, and with a ratio of the length of the ramp and the total length of the chirp pulse $T_{\mathrm{r}}/T_{\mathrm{adiab}} = 0.05$. **b** The relative errors for slow motion. For faster motions the errors are scaled down by the factor $F_{\eta }$ or $F_J$ shown in (**a**)

For slow motions, when $J(0)\gg J(\omega _{\mathrm{N}})$, the factors converge to $F_{\eta } = F_J = 1$ as shown by two examples in Fig. 2a. The protocol employed here (Palmer et al. 2001) ensures that a miscalibration of the $B_1$ field larger than 100 Hz is unlikely. Therefore, the factor $(1-\alpha /\alpha ^{\prime })$ was evaluated within the limits $|B_1-B^{\prime }_1|<100\,\hbox {Hz}$, where $B^{\prime }_1$ is an erroneously determined amplitude of the chirp pulse. Fig. 2b shows that errors arising from such $B_1$ field miscalibrations are often negligible. Errors increase with increasing sweep width, decreasing $B_1$ field, and decreasing ratio of the length of ramp and the total length of chirp pulse $T_{\mathrm{r}}/T_{\mathrm{adiab}}$. For clarity only the case $T_{\mathrm{r}}/T_{\mathrm{adiab}}=0.05$ is shown in Fig. 2b.

**Fig. 3:**
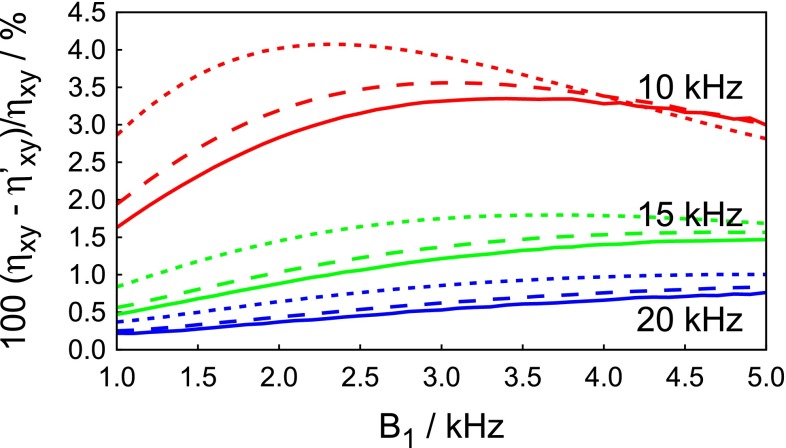
Simulated dependence of the relative errors of $\eta _{xy}$ determined by the adiabatic experiment (scheme 1) on the amplitude of the adiabatic pulse [the relative errors of *J*(0) were almost identical]. The simulations refer to 1 GHz, the $^{15}\hbox {N}$ resonance was shifted by 15  ppm from the centre of the frequency sweep of the adiabatic pulse. The *red, green, and blue lines* show the errors predicted for a 80 ms chirp pulse with sweep widths of 10, 15, and 20 kHz, respectively. The *solid, dashed and dotted lines* show errors for chirp pulses with the ratios of the length of the ramp and the total duration of the chirp pulse $T_{\mathrm{r}}/T_{\mathrm{adiab}} = 0.05, 0.1$, and 0.2, respectively

As mentioned in the “Theory” section, the average Liouvillian differs from Eq. 19 for residues with $^{15}\hbox {N}$ resonance frequencies that are not in the centre of the adiabatic sweep. Therefore, a systematic error arising from off-resonance effects must be considered. The error was simulated for the case of a $^{15}\hbox {N}$ nucleus with a 15 ppm resonance offset from the centre of the adiabatic sweep. The single correlation time of the motion was varied over the range $1<\tau _0<50\,\hbox {ns}$. The dependence of the error on the correlation time between these limits was monotonic but non-linear, suggesting that the simulation for $\tau _0=50\,\hbox {ns}$ is close to the slow motion limit. The dependence of the error on the $B_1$ amplitude of the chirp pulses is shown in Fig. 3 for $\tau _0 =10\,\hbox {ns}$ and $B_0 = 23.5\,\hbox {T}$ (1 GHz $^{1}\hbox {H}$ Larmor frequency). The results for $B_0 = 18.79$ and 11.74 T (800 and 500 MHz $^{1}\hbox {H}$ Larmor frequencies) follow the same trends but errors are scaled down so that they do not exceed 2.7 and 1.1 %, respectively. The absolute values of the relative errors calculated for extreme correlation times $\tau _0 = 1$ and 50 ns do not exceed 1.8 and 4.3 %, respectively, even at 1 GHz.

**Fig. 4:**
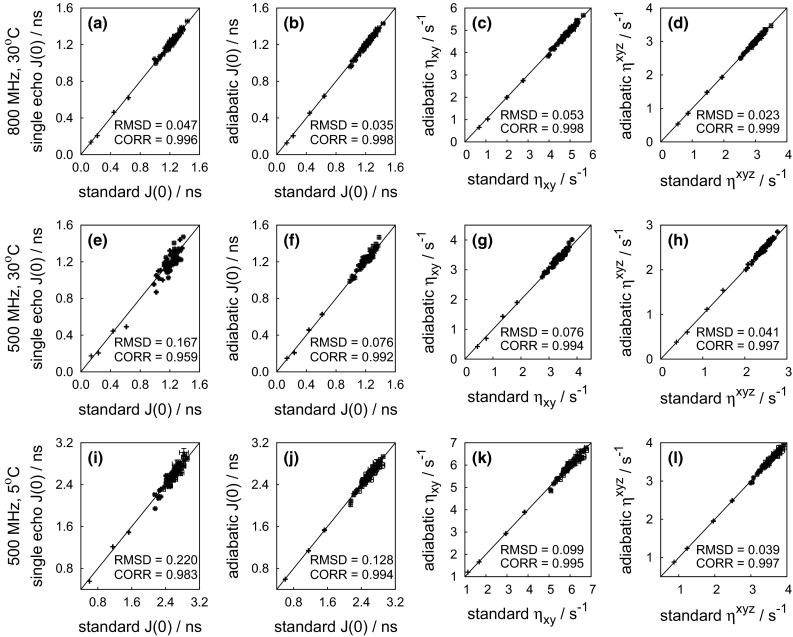
Correlations between the results of our experiments and standard experiments. Panels **a–d**, **e–h** and **i–l** correspond to the results measured for ubiquitin at 800 MHz and $30\,^{\circ }\hbox {C}$, 500 MHz and $30\,^{\circ }\hbox {C}$, and 500 MHz and $5\,^{\circ }\hbox {C}$, respectively. The comparisons of *J*(0) measured using the single-echo variant of our pulse program with non-selective nitrogen pulses and with adiabatically swept pulse are shown in panels (**a, e, i**) and (**b, f, j**), respectively. The correlations of the transverse CCCR are shown in panels (**c, g, k**) and the linear combinations of transverse and longitudinal CCCR rates are in panels (**d, h, l**). Root mean square deviation (RMSD) and correlation parameter (CORR) are shown in each panel

The theory is valid for an isolated pair of spins. An amide $^{15}\hbox {N}$–$^{1}\hbox {H}$ spin pair in an otherwise deuterated protein sample fulfills these assumptions to a very good approximation. However, effects of $\hbox {H}^\alpha$ and other protons should be considered for partially deuterated or fully protonated protein samples. We performed simulations for a spin system where dipole-dipole interactions with two additional protons were considered. The protons were placed 2.1, 2.5, and 2.9 Å from both amide nitrogen and hydrogen. The three studied cases approximate fully protonated, partially deuterated, and fully deuterated protein samples, respectively. First, the simulations show (see Supplementary information) that the error rises significantly with decreasing distance between the amide spin pair and the additional interacting protons. Second, the accuracy decreases with increasing correlation time of a motion of an amide spin pair. Third, the error rises with the length of the relaxation delay *T*, and finally, the error is larger if an adiabatic pulse with a smaller $\alpha$ parameter is used (selected dependences of the error on the correlation time and the adiabatic pulse parameters are shown in Supplementary information). Generally, the results of the simulations show that a high level of deuteration is required for a quantitative analysis if methods with adiabatically swept pulses (scheme 1) are used. Although a partial deuteration can suffice in small proteins, scheme 2 should be favoured for a protonated or partially deuterated sample. The enhanced performance of scheme 2 comes from the averaging of the auto-relaxation rates of the in-phase and anti-phase terms effectively performed by the evolution under scalar coupling. The use of a conversion block between in-phase and anti-phase terms (Kroenke et al. 1998; Ghose et al. 1999) in scheme 1 should enhance the accuracy of the measurements.

Finally, the effect of amide proton exchange was considered. The simulations show that the results of experiments with an adiabatically swept pulse (scheme 1) are corrupted in the presence of very fast proton exchange. The error rises with increasing proton exchange rate and decreasing correlation time of the studied amide spin pair. The error is larger for longer relaxation delays *T* and/or if an adiabatic pulse with a smaller $\alpha$ parameter is used (see Supplementary information). For a typical experimental set up similar to the one used in this study (simulated for an experiment performed at 500 MHz with an adiabatic pulse with the following parameters: $B_1=2.3\,\hbox {kHz}$, $SW=10\,\hbox {kHz}$, $T_{\mathrm{adiab}}=80\,\hbox {ms}$, $T_{\mathrm{r}}/T_{\mathrm{adiab}}=0.2$) a maximum exchange rate of 4 Hz was tolerable to keep the error below 3.5 % for residues with correlation time $\tau =1\,\hbox {ns}$. Because faster proton exchange rates are typical for disordered proteins (Croke et al. 2005) the scheme 2 should be the method of choice in this case. The error was lower than 1.5 % even for the simulation with a proton exchange rate equal to 20 s^−1^, a correlation time $\tau =1\,\hbox {ns}$, and a relaxation delay $T=120\,\hbox {ms}$. Like for proton-proton dipolar relaxation, fast averaging of in-phase and anti-phase auto-relaxation rates makes scheme 2 more accurate in these cases.

### Experimental results

**Fig. 5:**
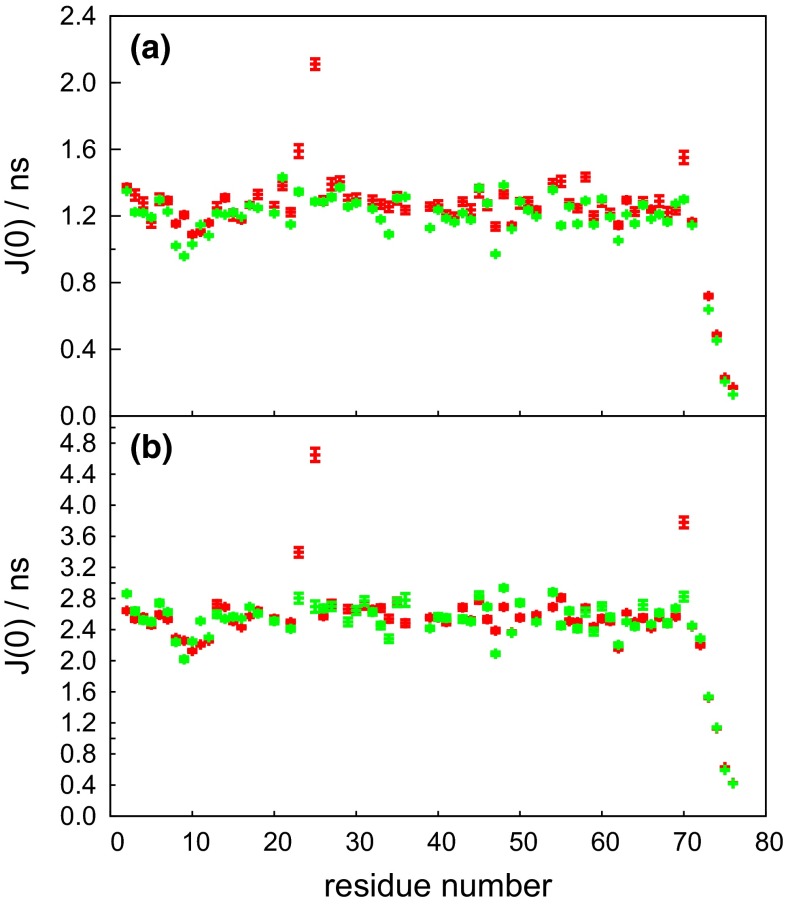
J(0) values in ubiquitin measured **a** at 800 MHz and $30\,^{\circ }\hbox {C}$, and **b** at 500 MHz and $5\,^{\circ }\hbox {C}$ as a function of the residue number. The *red symbols* represent spectral density values obtained by reduced spectral density mapping, the *green symbols* correspond to data measured by our experiment using an adiabatically swept pulse

The experiments were first tested on ubiquitin. Spectral density values *J*(0) and experimental relaxation rates $\eta _{xy}$, and $\eta ^{xyz}_{\mathrm{ave}}$ were compared with the expected values derived from transverse and longitudinal CCCR rates measured with standard experiments. The values obtained by both adiabatic and single echo experiments (schemes 1 and 2 in Fig. 1) are in good agreement with the conventional approach (Fig. 4). As expected, the *J*(0) values obtained from any analysis based on CCCR rates are not contaminated by chemical exchange, in contrast to *J*(0) values obtained from classical reduced spectral density mapping (Ishima and Nagayama 1995b, a; Farrow et al. 1995). A comparison of the determined *J*(0) values is shown in Fig. 5.

**Fig. 6:**
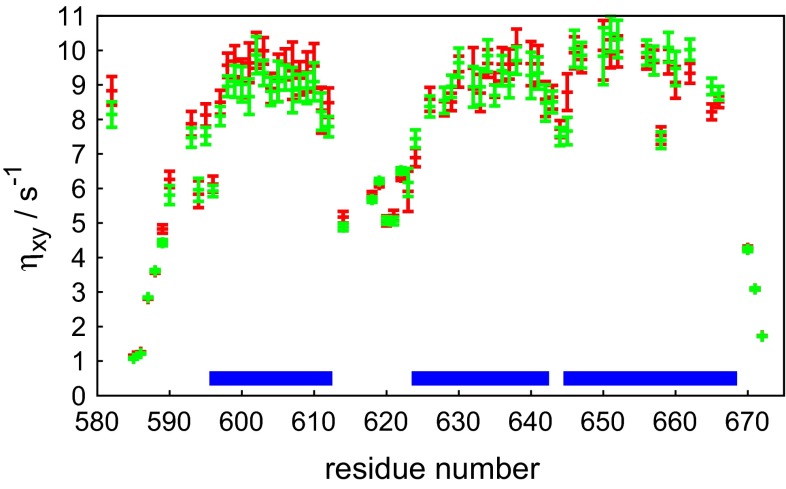
Transverse cross-correlated cross-relaxation rates for different residues in the KIX domain. The *red and green symbols* show data measured by the standard method and by our pulse program using adiabatically swept pulse in the relaxation period. The *blue horizontal bars* show the positions of three main $\alpha$-helices in the KIX domain

Our method was also applied to the KIX domain of CBP, which is known to have a significant amount of slow conformational exchange (Tollinger et al. 2006). The transverse CCCR rates were measured with the adiabatic experiment (scheme 1 in Fig 1) and conventional experiments. The results displayed in Fig. 6 show a very good agreement between the two methods. However, our experiment yields $\eta _{xy}$ values that are systematically lower for residues in the first $\alpha$ helix of KIX (the average difference is $0.4\,\hbox {s}^{-1}$), while the agreement between the data in other parts of the sequence is satisfactory. It has been reported (Tollinger et al. 2006) that helices 1 and 2 undergo a slow exchange between a folded and a partially un-folded state. H/D exchange protecting factors (Schanda et al. 2008) were shown to be higher in helix 2 than helix 1, suggesting the persistence of residual structure for helix 2 in the partially unfolded state.

In order to identify the source of the disagreement, simulations of both experiments were performed. Two-state exchange was included in the Liouvillian matrix and the exchange and relaxation parameters were chosen to correspond to published values for KIX (Tollinger et al. 2006). Relaxation due to fast motions (ps–ns) was treated following the semi-classical relaxation theory (Wangsness and Bloch 1953; Redfield 1965) assuming that only an unrestricted isotropic motion with a single correlation time $\tau _0$ contributes to relaxation. Two simulations were performed in which the slow exchange parameters were identical, but the parameters defining the fast motions differed. In the first simulation, the correlation times of both exchanging states were equal ($\tau _0=10\,\hbox {ns}$), while in the second case the motion of the less populated state was ten times faster ($\tau _0=1\,\hbox {ns}$). The results of the simulations of both the standard experiment and of the adiabatic experiment (scheme 1 in Fig. 1) were identical in the first case but different in the second case. While the simulations of the standard experiment provide very similar results in both cases, the result of the adiabatic experiment is affected by the short correlation time of the less populated excited state. If the standard method to measure $\eta _{xy}$ is used, both in-phase and anti-phase coherences of the excited state are lost rapidly due to enhanced relaxation caused by the exchange. This effect reduces the weight of cross-relaxation in the excited state to a negligible value. So, the measured value reflects mostly the major folded state. On the contrary, the adiabatic sweep efficiently suppresses the loss of coherence due to exchange in the minor state. Therefore, the measured value represents a population-weighted $\eta _{xy}$ rate that is determined by both the ground and excited states, provided the exchange rate is fast enough to explore both states during the relaxation delay.

Theoretically, knowledge of the major state $\eta _{xy}$, of the population weighted $\eta _{xy}$, and the population of the excited state [based on relaxation dispersion experiments (Tollinger et al. 2006; Schanda et al. 2008)] allows one to calculate the CCCR rate of the excited state. Unfortunately, the precision of our measurements is not sufficient to obtain reasonably precise CCCR rates of the excited state. Nevertheless, the systematic deviations of the $\eta _{xy}$ values between the adiabatic experiment (scheme 1 in Fig. 1) and the standard experiment clearly demonstrate the dynamic character of the first $\alpha$ helix in the excited state. It is well known from relaxation dispersion and hydrogen/deuterium exchange experiments (Tollinger et al. 2006; Schanda et al. 2008) that this $\alpha$ helix is unfolded in the excited state, however, to the best of our knowledge, no proof had been obtained so far of any enhanced ps–ns motions of this $\alpha$ helix in the excited state. Interestingly, we find that helix $\alpha _2$, which is expected to be in fast exchange between unfolded and folded states (both roughly 50 % populated; Schanda et al. 2008), does not show a significant change of its average ps–ns dynamics in the excited state. Not surprisingly, helix $\alpha _3$ was found to be as rigid in the excited state (where it remains mostly $\alpha$-helical) as in the folded state.

**Fig. 7:**
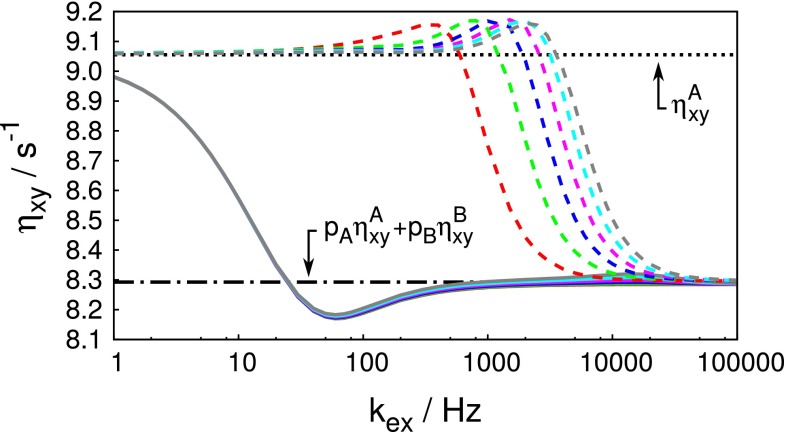
Simulated dependence of the transverse CCCR rate $\eta _{xy}$ obtained by the standard experiment (*dashed lines*) and our experiment (Eq. 25) using adiabatically swept pulse (*solid lines*) on the rate of the exchange. The simulations were performed for a static field of 11.75 T, an isolated $^{15}\hbox {N}{-}^{1}\hbox {H}$ spin system undergoing a two-state exchange $\hbox {A}\leftrightarrow \hbox {B}$ characterized by equilibrium populations $p_{\mathrm{A}} = 0.9$ and $p_{\mathrm{B}} = 0.1$, the $^{15}\hbox {N}$ chemical shift of the major state was in the middle of the chirp sweep width and the frequency of the minor state was shifted by 100 Hz (*red*), 200 Hz (*green*), 300 Hz (*blue*), 400 Hz (*magenta*), 500 Hz (*cyan*), and 600 Hz (*grey*). The simulations were done for a chirp pulse with sweep width $W=10\,\hbox {kHz}$, amplitude $B_1=2.3\,\hbox {kHz}$, proportion of the relative length of the apodization ramps $T_{\mathrm{r}}/T_{\mathrm{adiab}}=0.2$, and total length of the chirp pulse $T_{\mathrm{adiab}}=80\,\hbox {ms}$. The transverse CCCR rates were calculated for correlation times $\tau _0=10$ and 1 ns for the major and minor states, respectively. The *black dotted and dash and dot lines* represent the transverse CCCR rate of the major state $\eta ^{\mathrm{A}}_{xy}$ and population averaged value $p_{\mathrm{A}}\eta ^{\mathrm{A}}_{xy}+p_{\mathrm{B}}\eta ^{\mathrm{B}}_{xy}$, respectively

Several simulations were performed with various populations, chemical shift differences, and rates of exchange. An example is shown in Fig. 7. Generally, it was found that our adiabatic experiment (scheme 1 in Fig. 1) provides a good estimate of the population weighted CCCR rate unless the exchange rate is very slow ($k_{\mathrm{ex}} \lesssim 1/T_{\mathrm{adiab}}$), in which case the system does not ’hop’ frequently enough to effectively average $\eta _{xy}$. The standard experiment provides a value close to $\eta _{xy}$ of the major state, unless the process is fast enough to reach the fast exchange regime.

For comparison of the sensitivity, the relaxation delay *T* of the standard experiment was set to $T = \alpha T_{\mathrm{adiab}}$. A comparison of the intensities are shown in Supplementary information. Generally, the intensities of residues that are not affected by slow exchange should always be larger in spectra measured by the standard experiment because its total relaxation delay $T = \alpha T_{\mathrm{adiab}}$ is shorter than the total relaxation delay *T* of scheme 1 in Fig. 1. The difference is most pronounced for flexible residues, which typically have intense signals. Because the relaxation of the two-spin order $2N_zH_z$ is faster than the relaxation of the $N_z$ polarization, the most dramatic attenuation affects the intensities $I_{P,Q}$ where both *P* and *Q* represent $2N_zH_z$, which usually show the most intense signals in symmetrical reconversion. Therefore, a drop of the sensitivity compared to the standard experiment observed for almost all peaks in this spectrum is not critical. However, if slow exchange contributes significantly to relaxation the adiabatic method becomes the method of choice since the irradiation diminishes the dominant relaxation mechanism. The precision of the symmetrical reconversion method is mostly limited by the weak intensities of $I_{P,Q}$ and $I_{Q,P}$, where *P* stands for $N_z$ and *Q* stands for $2N_zH_z$. The intensity was improved for 46 out of 68 peaks and only 3 peaks exhibit an intensity decrease larger than 10 % in the spectra, where $N_z$ and $2N_zH_z$ terms were selected before and after the relaxation period. A similar proportion of the peaks (43 out of 68) with an increased intensity was found in spectra corresponding to the complementary cross-relaxation pathway ($2N_zH_z$ term selected before the relaxation period and $N_z$ after the relaxation period). A decrease of intensity larger than 10 % was found only for 9 peaks in this case. Finally, the intensities of 97 % peaks were increased in spectra where $N_z$ magnetization was selected both before and after the relaxation period.

Note that $\eta _{xy}$ and $\eta _z$ can be also determined from two experiments with adiabatic pulses without the flanking $T_z$ periods. In that case the relaxation during $T_z$ is avoided, while the advantage of the suppression of exchange is preserved. A measurement of $\eta _z$ by standard experiments and a linear combination of $\eta _{xy}$ and $\eta _z$ by adiabatic experiments is another possibility which keeps the advantage of an independent and direct determination of $\eta _z$.

## Conclusions

We introduced a series of experiments to quantify cross-correlated cross-relaxation CCCR rates under adiabatically swept pulses. These experiments allow one to extend applications of such pulses to the study of fast (ps–ns) motions in proteins. The adiabatic experiment for the determination of transverse CCCR rates provides results that are consistent with standard experiments. The adiabatic sweeps ensure almost uniform conditions over the whole range of amide chemical shifts even at the highest fields accessible. The method was demonstrated for the KIX domain and human ubiquitin measured at room and low temperatures to mimic different dynamic behaviours. Two different advantages of our approach could be useful in the presence of slow chemical exchange. First, the sensitivity is improved thanks to the suppression of a loss of phase coherence due to slow exchange. Second, the measured transverse CCCR rates represent ensemble-averaged values over all states that undergo slow exchange except if the exchange rates are very small. Finally, the experiment can be modified to yield spectral density values at zero frequency *J*(0) directly free of any bias due to exchange. In addition, a single experiment is sufficient for the determination of *J*(0). Precise values *J*(0) can be extracted even when the standard analysis is limited by the precision of the measurement of longitudinal CCCR rates.

The methods with an adiabatically swept pulse are best suited for deuterated protein samples because interactions with further spins may introduce significant systematic errors. In addition, the presented adiabatic methods are not well suited for rapidly moving proteins undergoing fast amide proton exchange (>4 s^−1^) which is typical of intrinsically disordered proteins close to physiological conditions. In both these cases the standard experiment for a measurement of CCCR rates (Pelupessy et al. 2003, 2007) or the variant of the presented method for a direct *J*(0) measurement without adiabatically swept pulse should be used.

### Electronic supplementary material

Supplementary material 1 (pdf 287 KB)

## Appendix

### Derivation of errors due to erroneous $\alpha$ parameter

$\alpha$: the true parameter
$\alpha ^{\prime }$: the assumed parameter
$\eta _{xy}^{\prime }$: the result of the experiment performed on the assumption that $\alpha ^{\prime }$ is correct
$J^{\prime }(0)$: the result of the experiment performed on the assumption that $\alpha ^{\prime }$ is correct

The experiment for the determination of the CCCR rate $\eta _{xy}$ provides $\eta _{\mathrm{ave}}^{xy}$:

32 $$\begin{aligned} \eta ^{xy}_{\mathrm{ave}}=\frac{(1-\alpha ^{\prime })\eta _z - (\alpha \eta _{xy} + (1-\alpha )\eta _z)}{1+1-\alpha ^{\prime }} \end{aligned}$$

and $\eta _{xy}^{\prime }$ is calculated according to Eq. 25:

33 $$\begin{aligned} \eta _{xy}^{\prime }=\eta ^{xy}_{\mathrm{ave}}\frac{\alpha ^{\prime }-2}{\alpha ^{\prime }} \end{aligned}$$

Then, a relative error of $\eta _{xy}^{\prime }$ with respect to the correct value is given by:

34 $$\begin{aligned} \frac{\eta _{xy}-\eta _{xy}^{\prime }}{\eta _{xy}}=\left( 1- \frac{\alpha }{\alpha ^{\prime }}\right) \left( 1- \frac{\eta _z}{\eta _{xy}}\right) \end{aligned}$$

A similar approach is used for the derivation of an error *J*(0):

35 $$\begin{aligned} \eta ^{J}_{\mathrm{ave}}=\frac{(1-0.5\alpha ^{\prime })\eta _z - (\alpha \eta _{xy} + (1-\alpha )\eta _z)}{1+1-0.5\alpha ^{\prime }} \end{aligned}$$

providing $J^{\prime }(0)$ calculated according to Eq. 26:

36 $$\begin{aligned} J^{\prime }(0)=\eta ^J_{\mathrm{ave}}\frac{\alpha ^{\prime }-4}{16C_{cd}\alpha ^{\prime }} \end{aligned}$$

Finally, a relative error with respect to the correct value $J(0) = (\eta _{xy}-0.5\eta _z)/8C_{cd}$ is calculated as:

37 $$\begin{aligned} \frac{J(0)-J^{\prime }(0)}{J(0)}=\left( 1- \frac{\alpha }{\alpha ^{\prime }}\right) \left( 1- \frac{\eta _z}{2\eta _{xy}-\eta _z}\right) \end{aligned}$$

The expressions in Eqs. 28 and 29 can be derived simply by substitution of $\eta _{xy}$ and $\eta _z$ according to Eqs. 6 and 7 into Eqs. 34 and 37, respectively.

## References

[CR1] Abragam A (1961). The principles of nuclear magnetism.

[CR2] Auer R, Kloiber K, Vavrinska A, Geist L, Coudevylle N, Konrat R (2010). Pharmacophore mapping via cross-relaxation during adiabatic fast passage. J Am Chem Soc.

[CR3] Auer R, Tollinger M, Kuprov I, Konrat R, Kloiber K (2011). Mathematical treatment of adiabatic fast passage pulses for the computation of nuclear spin relaxation rates in proteins with conformational exchange. J Biomol NMR.

[CR4] Böhlen JM, Bodenhausen G (1993). Experimental aspects of chirp NMR spectroscopy. J Magn Reson Ser A.

[CR5] Böhlen JM, Burghardt I, Rey M, Bodenhausen G (1990). Frequency modulated “Chirp” pulses for broadband inversion recovery in magnetic resonance. J Magn Reson.

[CR6] Croke RL, Sallum CO, Watson E, Watt ED, Alexandrescu AT (2005). Hydrogen exchange of monomeric $$\alpha $$-synuclein shows unfolded structure persists at physiological temperature and is independent of molecular crowding in *Escherichia coli*. Protein Sci..

[CR7] Delaglio F, Grzesiek S, Vuister GW, Zhu G, Pfeifer J, Bax A (1995). NMRPipe: a multidimensional spectral processing system based on UNIX pipes. J Biomol NMR.

[CR8] Eaton JW, Bateman D, Hauberg S (2009). GNU Octave version 3.0.1 manual: a high-level interactive language for numerical computations.

[CR9] Farrow NA, Zhang O, Szabo A, Torchia DA, Kay LE (1995). Spectral density function mapping using $$^{15}\text{ N }$$ relaxation data exclusively. J Biomol NMR.

[CR10] Ferrage F, Cowburn D, Ghose R (2009). Accurate sampling of high frequency motions in proteins by steady-state $$^{15}\text{ N }{}^{1}\text{ H }$$ nuclear Overhauser effect measurements in the presence of cross-correlated relaxation. J Am Chem Soc.

[CR12] Ghose R, Eykyn TR, Bodenhausen G (1999). Average Liouvillian theory revisited: cross-correlated relaxation between chemical shift anisotropy and dipolar couplings in the rotating frame in nuclear magnetic resonance. Mol Phys.

[CR13] Ghose R (2000). Average Liouvillian theory in nuclear magnetic resonance - Principles, properties, and applications. Concepts Magn Reson.

[CR14] Goddard TD, Kneller DG (2006) Sparky. UCSF, San Francisco

[CR15] Halle B (2009). The physical basis of model-free analysis of NMR relaxation data from proteins and complex fluids. J Chem Phys.

[CR16] Halle B, Andersson T, Forsén S, Lindman B (1981). Protein hydration from water oxygen-17 magnetic relaxation. J Am Chem Soc.

[CR17] Hogben HJ, Krzystyniak M, Charnock GTP, Hore PJ, Kuprov I (2011). Spinach A software library for simulation of spin dynamics in large spin systems. J Magn Reson.

[CR18] Ishima R, Nagayama K (1995). Protein backbone dynamics revealed by quasi spectral density function analysis of amide N-15 nuclei. Biochemistry.

[CR19] Ishima R, Nagayama K (1995). Quasi-spectral-density function analysis for nitrogen-15 nuclei in proteins. J Magn Reson Ser B.

[CR20] Kadeřávek P, Zapletal V, Rabatinová A, Krásný L, Sklenář V, Žídek L (2014). Spectral density mapping protocols for analysis of molecular motions in disordered proteins. J Biomol NMR.

[CR21] Konrat R, Tollinger M (1999). Heteronuclear relaxation in time-dependent spin systems: $$^{15}\text{ N }$$-$$\text{ T }_{1\rho }$$ dispersion during adiabatic fast passage. J Biomol NMR.

[CR22] Korzhnev DM, Billeter M, Arseniev AS, Orekhov VY (2001). NMR studies of Brownian tumbling and internal motions in proteins. Prog Nucl Magn Reson Spectrosc.

[CR23] Kroenke CD, Loria JP, Lee LK, Rance M, Palmer AG (1998). Longitudinal and transverse $$^{1}\text{ H }$$-$$^{15}\text{ N }$$ dipolar/$$^{15}\text{ N }$$ chemical shift anisotropy relaxation interference: Unambiguous determination of rotational diffusion tensors and chemical exchange effects in biological macromolecules. J Am Chem Soc.

[CR24] Kupče E, Freeman R (1995). Adiabatic pulses for wide-band inversion and broad-band decoupling. J Magn Reson Ser A.

[CR25] Lakomek NA, Ying J, Bax A (2012). Measurement of $$^{15}\text{ N }$$ relaxation rates in perdeuterated proteins by TROSY-based methods. J Biomol NMR.

[CR26] Levitt MH, Bari LD (1992). Steady state in magnetic-resonance pulse experiments. Phys Rev Lett.

[CR27] Lipari G, Szabo A (1982). Model-free approach to the interpretation of nuclear magnetic resonance relaxation in macromolecules 1. Theory and range of validity. J Am Chem Soc.

[CR28] Lipari G, Szabo A (1982). Model-free approach to the interpretation of nuclear magnetic resonance relaxation in macromolecules 2. Analysis of experimental results. J Am Chem Soc.

[CR29] Mangia S, Traaseth NJ, Veglia G, Garwood M, Michaeli S (2010). Probing slow protein dynamics by adiabatic $$R_{1\rho }$$ and $$R_{2\rho }$$ NMR experiments. J Am Chem Soc.

[CR30] MATLAB and statistics toolbox release R2014a, The MathWorks, Inc, Natick, Massachusetts, United States

[CR31] Mulder FAA, Mittermaier A, Hon B, Dahlquist FW (2001). Studying excited states of proteins by NMR spectroscopy. Nat Struct Biol.

[CR32] Palmer AG, Kroenke CD, Loria JP (2001). Nuclear magnetic resonance methods for quantifying microsecond-to-millisecond motions in biological macromolecules. Methods Enzymol.

[CR33] Pelupessy P, Espallargas GM, Bodenhausen G (2003). Symmetrical reconversion: measuring cross-correlation rates with enhanced accuracy. J Magn Res.

[CR34] Pelupessy P, Ferrage F, Bodenhausen G (2007). Accurate measurement of longitudinal cross-relaxation rates in nuclear magnetic resonance. J Chem Phys.

[CR35] Peng JW, Wagner G (1992). Mapping of spectral density-functions using heteronuclear NMR relaxation measurements. J Magn Reson.

[CR36] Peng JW, Wagner G (1992). Mapping of the spectral densities of N–H bond motions in Eglin* c* using heteronuclear relaxation experiments. Biochemistry.

[CR37] Redfield AG (1965). The theory of relaxation processes. Adv Magn Reson.

[CR38] Schanda P, Brutscher B, Konrat R, Tollinger M (2008). Folding of the KIX domain: characterization of the equilibrium analog of a folding intermediate using $$^{15}\text{ N }/{}^{13}\text{ C }$$ relaxation dispersion and fast $$^{1}\text{ H }/{}^2\text{ H }$$ amide exchange NMR spectroscopy. J Mol Biol.

[CR39] Tollinger M, Kloiber K, Agoston B, Dorigoni C, Lichtenecker R, Schmid W, Konrat R (2006). An isolated helix persists in a sparsely populated form of KIX under native conditions. Biochemistry.

[CR40] Valentine ER, Ferrage F, Massi F, Cowburn D, Palmer AG (2007). Joint composite-rotation adiabatic-sweep isotope filtration. J Biomol NMR.

[CR41] Wangsness R, Bloch F (1953). The dynamical theory of nuclear induction. Phys Rev Lett.

[CR42] Wolfram Research Inc. (2012) Mathematica

