# Supplementary material for: Cross-correlated relaxation measurements under adiabatic sweeps: determination of local order in proteins
Source: J Biomol NMR. 2015 Oct 28;63(4):353–65. doi: 10.1007/s10858-015-9994-8 (PMC4662729; doi:10.1007/s10858-015-9994-8)
Supplement: Supplementary file 1 — Supplementary material 1 (pdf 287 KB) [file 10858_2015_9994_MOESM1_ESM.pdf]

Supplementary information to: J Biomol NMR

Cross-correlated relaxation measurements under adiabatic sweeps: determination of local order in proteins

Pavel Kadeřávek, Sarina Grutsch, Nicola Salvi, Martin Tollinger, Lukáš Žídek, Geoffrey Bodenhausen, Fabien Ferrage

Pavel Kadeřávek · Lukáš Žídek
National Centre for Biomolecular Research, Faculty of Science and Central European Institute of Technology, Masaryk University, Kamenice 5, 625 00 Brno, Czech Republic

Pavel Kadeřávek · Nicola Salvi · Geoffrey Bodenhausen
Institut des Sciences et Ingénierie Chimiques, École polytechnique fédérale de Lausanne, CH-1015 Lausanne, Switzerland

Sarina Grutsch · Martin Tollinger
Institute of Organic Chemistry, Center for Molecular Biosciences Innsbruck (CMBI), University of Innsbruck, 6020 Innsbruck, Austria

Pavel Kadeřávek · Geoffrey Bodenhausen · Fabien Ferrage (✉)
École Normale Supérieure - PSL Research University, Département de Chimie, 24 rue Lhomond, 75005 Paris, France
Sorbonne Universités, UPMC Univ Paris 06, LBM, 4 place Jussieu, 75005 Paris, France
CNRS, UMR 7203 LBM, 75005 Paris, France, Tel.: +123-45-678910, Fax: +123-45-678910
e-mail: fabien.ferrage@ens.fr

a)

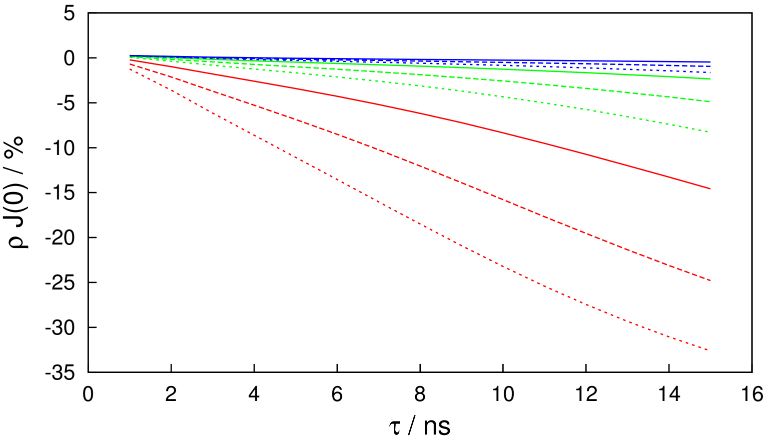

b)

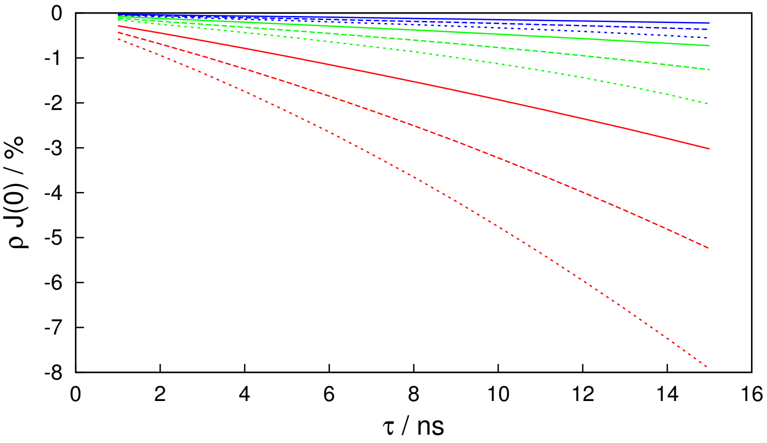

Figure S1: Dependence of the relative error of $J(0)$ on the correlation time τ of the motion (e.g. overall tumbling) in case of a non-isolated ^{15}N-^{1}H spin pairs for **a)** scheme 1 (with an adiabatic pulse) and **b)** scheme 2 (without an adiabatic pulse). Two additional protons were placed at a distance 2.1 Å(red), 2.5 Å(green), and 2.9 Å(blue) from both the amide nitrogen and proton. The solid, dashed, and dotted lines correspond to the simulations with T_{adiab} **a)** or T_{xy} **b)** equal to 40, 60, and 80 ms, respectively. The Chirp pulse was used with the proportion of the ramps $T_{\mathrm{r}}/T_{\mathrm{adiab}} = 0.2$, the sweep width $SW = 10\,\mathrm{kHz}$, and the maximum amplitude $B_1 = 2.3\,\mathrm{kHz}$ in the all presented cases of the simulations with the adiabatic pulse. The simulations (it is also valid for the results shown in the figures Fig S2–S6) were performed with MATLAB R2015A [MATLAB] using SPINACH 1.5.2440 [Hogben et al(2011)]. The full basis set was used with Redfield relaxation theory [Wangsness and Bloch(1953), Redfield(1965)] using the secular approximation. The internuclear distance $r_{\mathrm{N-H}}$ was set to 1.02 Å, the anisotropy of the nitrogen chemical shielding tensor to $\Delta\sigma = \sigma_{\parallel} - \sigma_{\perp} = -170\,\mathrm{ppm}$ and the angle between the unique axis of the CSA tensor and the N-H bond to 20.6°. The simulations were performed for a magnetic field $B_0 = 11.75\,\mathrm{T}$.

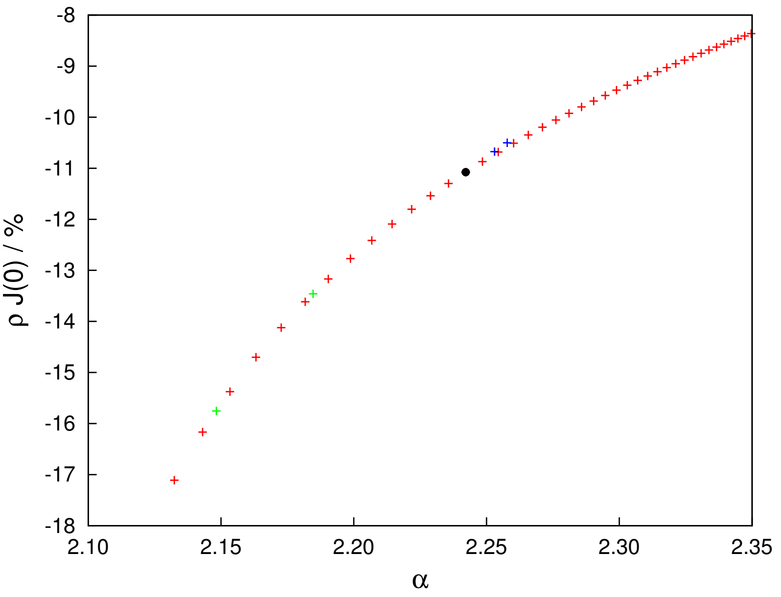

Figure S2: Dependence of the relative error of $J(0)$ value on the α parameter in case of a non-isolated ^{15}N-^{1}H spin pair. Two additional protons were placed at distance 2.1 Å from both the amide nitrogen and proton. The black circle represents a reference case of the simulation for an experiment using the Chirp adiabatic pulse of the total length $T_{\mathrm{adiab}} = 80\,\mathrm{ms}$, the proportion of ramps $T_{\mathrm{r}}/T_{\mathrm{adiab}} = 0.2$, the sweep width $SW = 10\,\mathrm{kHz}$, and the maximum amplitude $B_1 = 2.3\,\mathrm{kHz}$. The red, blue and green points represent an effect of the variation of B_1, $T_{\mathrm{r}}/T_{\mathrm{adiab}}$, and SW, respectively, while the other parameters were unchanged. B_1 was linearly varied between 1.0 and 5.0 kHz, SW was tested with values 15 and 20 kHz, and the ratio $T_{\mathrm{r}}/T_{\mathrm{adiab}}$ with the values 0.1 and 0.05.

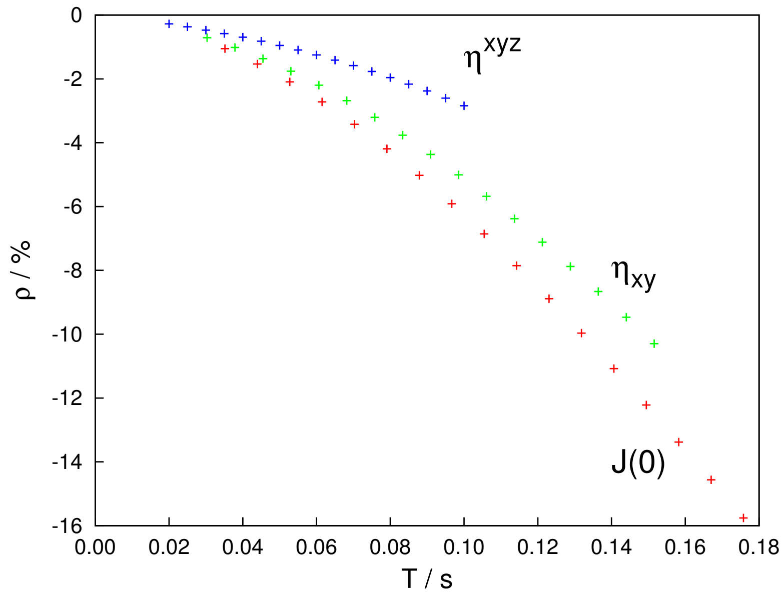

Figure S3: Dependence of the relative error of $J(0)$ (red), η_{xy} (green), and $\eta^{xyz} = \alpha\eta_{xy} + (1-\alpha)\eta_z$ on the relaxation delay T in case of a non-isolated ^{15}N-^{1}H spin pair. Two additional protons were placed 2.1 Å from both the amide nitrogen and proton. In all cases the length of the adiabatic Chirp pulse was varied between 20 and 100 ms (other parameters were constant: the proportion of ramps $T_{\mathrm{r}}/T_{\mathrm{adiab}} = 0.2$, the sweep width $SW = 10\,\mathrm{kHz}$, and the maximum amplitude $B_1 = 2.3\,\mathrm{kHz}$).

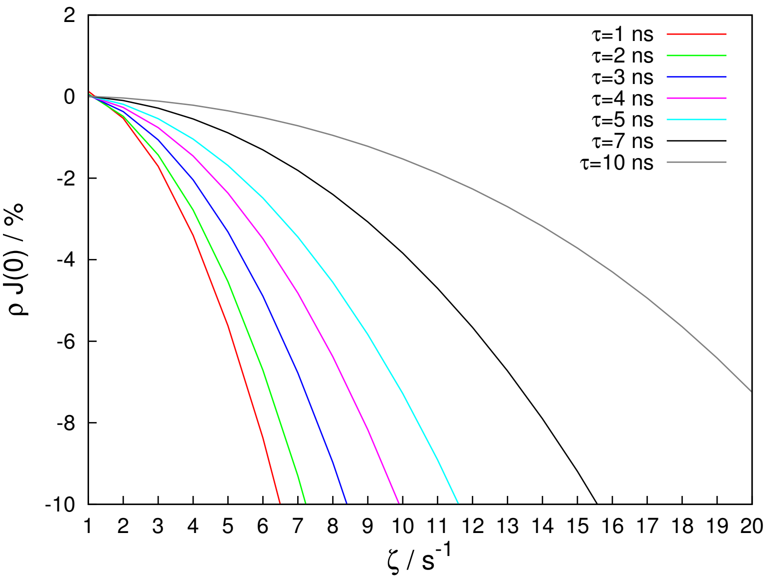

Figure S4: Dependence of the relative error of $J(0)$ on the proton exchange rate ζ. The simulations for various correlation time τ of the motion of an isolated ^{15}N-^{1}H amide spin pair are distinguished by colors. The 80 ms Chirp pulse was used with the proportion of the ramps $T_{\mathrm{r}}/T_{\mathrm{adiab}} = 0.2$, the sweep width $SW = 10\,\mathrm{kHz}$, and the maximum amplitude $B_1 = 2.3\,\mathrm{kHz}$.

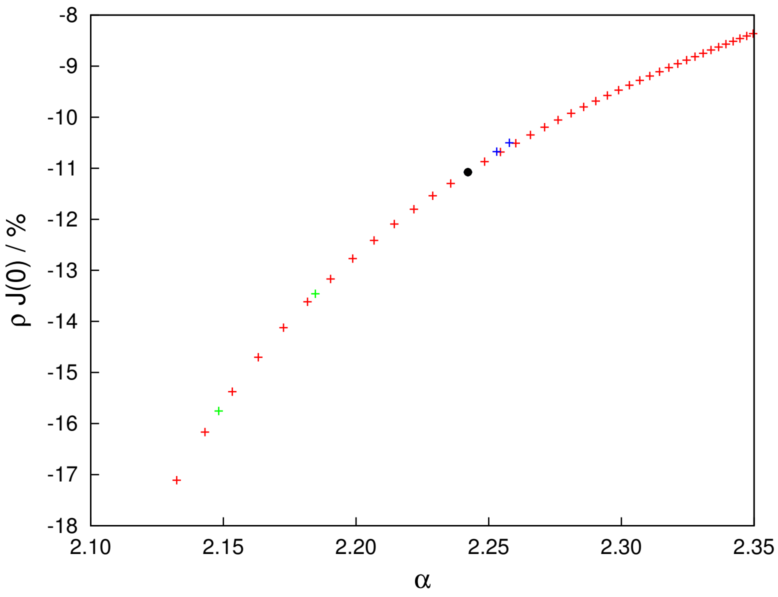

Figure S5: Dependence of the relative error of $J(0)$ value on the α parameter in case of a proton exchange rate $\zeta = 10\,\text{Hz}$. The black circle represents a reference case of the simulation for an experiment using the Chirp adiabatic pulse with the total length $T_{\text{adiab}} = 80\,\text{ms}$, proportion of the ramps $T_{\text{r}}/T_{\text{adiab}} = 0.2$, the sweep width $SW = 10\,\text{kHz}$, and the maximum amplitude $B_1 = 2.3\,\text{kHz}$. The red, blue and green points represent an effect of the variation of B_1, $T_{\text{r}}/T_{\text{adiab}}$, and SW, respectively, while the other parameters were unchanged. B_1 was linearly varied between 1.0 and 5.0 kHz, SW was tested with the values 15 and 20 kHz, and the ratio $T_{\text{r}}/T_{\text{adiab}}$ with the values 0.1 and 0.05.

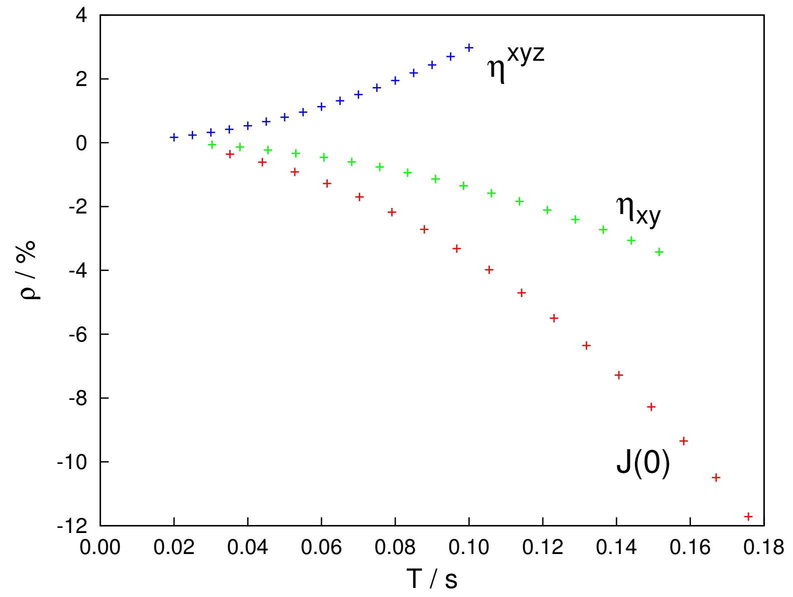

Figure S6: Dependence of the relative error of $J(0)$ (red), η_{xy} (green), and $\eta^{xyz} = \alpha\eta_{xy} + (1-\alpha)\eta_z$ on the relaxation delay T in case of a proton exchange rate $\zeta = 10\,\mathrm{Hz}$. In all cases the length of the adiabatic Chirp pulse was varied between 20 and 100 ms (other parameters were constant: the proportion of ramps $T_\mathrm{r}/T_\mathrm{adiab} = 0.2$, the sweep width $SW = 10\,\mathrm{kHz}$, and the maximum amplitude $B_1 = 2.3\,\mathrm{kHz}$).

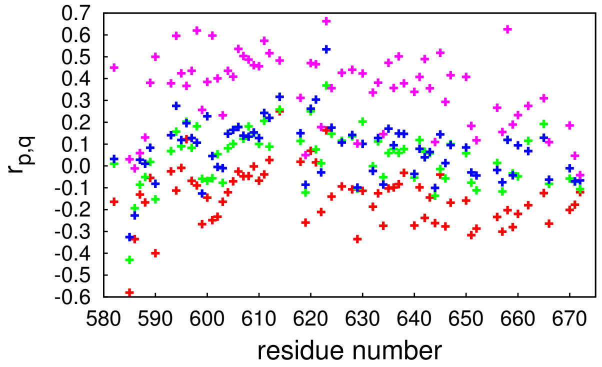

Figure S7: Dependence of the relative change of the intensities on the residue number of KIX, where $r_{p,q} = (I'_{p,q} - I_{p,q})/I_{p,q}$, $I_{p,q}$ and $I'_{p,q}$ are the intensities in the spectra obtained by standard and adiabatic experiment, respectively. The subscripts p and q denote intensity in spectra derived from terms p and q selected at the beginning and end of the relaxation period T: $p = N_z$ and $q = 2N_zH_z$ (blue), $p = N_z$ and $q = N_z$ (magenta), $p = 2N_zH_z$ and $q = N_z$ (green), $p = 2N_zH_z$ and $q = 2N_zH_z$ (red).

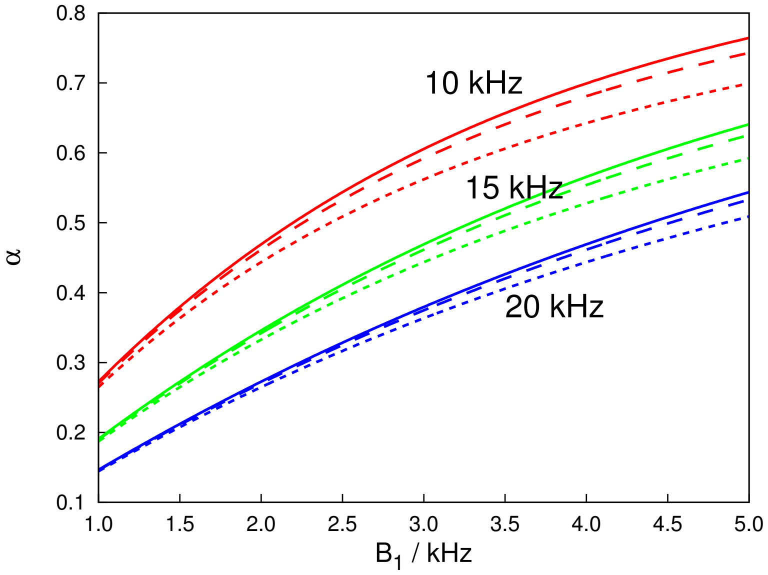

Figure S8: Dependence of the calculated parameters α describing the proportion between η_{xy} and η_z contributions during chirp pulses on the maximum amplitude B_1 of the pulse. The red, blue, and green lines stand for chirp pulses with the sweep widths $W = 10$, 15, and 20 kHz, respectively. The solid, dashed and dotted lines stand for the relative length of the amplitude ramps with respect to the total length of the pulse equal to $T_\mathrm{r}/T_\mathrm{adiab} = 0.05$, 0.1, and 0.2, respectively.

Table S1: Parameters α for selected adiabatic chirp pulses: Dependence of the calculated parameters α describing the relative contribution of η_{xy} and η_z as a function of the parameters of the chirp pulse. The lower and upper subscripts denote the parameter T_r/T_{adiab} (the proportion of the length of the amplitude ramp with respect to the total length of the pulse) and the total sweep width W (kHz), respectively. For instance $\alpha^{10}_{0.1}$ corresponds to a 10 kHz sweep width and $T_r = 0.1T_{adiab}$

B_1/Hz	$\alpha^{10}_{0.05}$	$\alpha^{10}_{0.1}$	$\alpha^{10}_{0.2}$	$\alpha^{15}_{0.05}$	$\alpha^{15}_{0.1}$	$\alpha^{15}_{0.2}$	$\alpha^{20}_{0.05}$	$\alpha^{20}_{0.1}$	$\alpha^{20}_{0.2}$
1000	0.2727	0.2704	0.2648	0.1908	0.1898	0.1872	0.1466	0.1460	0.1445
1010	0.2750	0.2727	0.2670	0.1926	0.1915	0.1889	0.1479	0.1473	0.1458
1020	0.2773	0.2749	0.2692	0.1943	0.1932	0.1905	0.1493	0.1487	0.1471
1030	0.2796	0.2772	0.2713	0.1960	0.1949	0.1921	0.1507	0.1500	0.1485
1040	0.2819	0.2794	0.2735	0.1977	0.1966	0.1938	0.1520	0.1514	0.1498
1050	0.2842	0.2817	0.2756	0.1994	0.1983	0.1954	0.1534	0.1527	0.1511
1060	0.2865	0.2839	0.2777	0.2011	0.2000	0.1971	0.1547	0.1541	0.1524
1070	0.2887	0.2862	0.2799	0.2028	0.2016	0.1987	0.1561	0.1554	0.1537
1080	0.2910	0.2884	0.2820	0.2045	0.2033	0.2003	0.1574	0.1567	0.1550
1090	0.2933	0.2906	0.2841	0.2062	0.2050	0.2019	0.1588	0.1581	0.1563
1100	0.2955	0.2928	0.2862	0.2079	0.2067	0.2035	0.1601	0.1594	0.1576
1110	0.2978	0.2950	0.2883	0.2096	0.2083	0.2052	0.1614	0.1607	0.1589
1120	0.3000	0.2972	0.2903	0.2113	0.2100	0.2068	0.1628	0.1620	0.1602
1130	0.3022	0.2994	0.2924	0.2130	0.2116	0.2084	0.1641	0.1634	0.1615
1140	0.3044	0.3015	0.2945	0.2146	0.2133	0.2100	0.1655	0.1647	0.1628
1150	0.3067	0.3037	0.2965	0.2163	0.2149	0.2116	0.1668	0.1660	0.1641
1160	0.3089	0.3059	0.2986	0.2180	0.2166	0.2131	0.1681	0.1673	0.1653
1170	0.3111	0.3080	0.3006	0.2197	0.2182	0.2147	0.1694	0.1686	0.1666
1180	0.3133	0.3102	0.3027	0.2213	0.2199	0.2163	0.1708	0.1699	0.1679
1190	0.3154	0.3123	0.3047	0.2230	0.2215	0.2179	0.1721	0.1713	0.1692
1200	0.3176	0.3144	0.3067	0.2246	0.2231	0.2195	0.1734	0.1726	0.1704
1210	0.3198	0.3166	0.3087	0.2263	0.2248	0.2210	0.1747	0.1739	0.1717
1220	0.3220	0.3187	0.3107	0.2279	0.2264	0.2226	0.1761	0.1752	0.1730
1230	0.3241	0.3208	0.3127	0.2296	0.2280	0.2242	0.1774	0.1765	0.1743
1240	0.3263	0.3229	0.3147	0.2312	0.2296	0.2257	0.1787	0.1778	0.1755
1250	0.3284	0.3250	0.3166	0.2329	0.2312	0.2273	0.1800	0.1791	0.1768
1260	0.3305	0.3270	0.3186	0.2345	0.2329	0.2288	0.1813	0.1804	0.1780
1270	0.3326	0.3291	0.3206	0.2361	0.2345	0.2304	0.1826	0.1817	0.1793
1280	0.3348	0.3312	0.3225	0.2377	0.2361	0.2319	0.1839	0.1829	0.1806
1290	0.3369	0.3332	0.3244	0.2394	0.2377	0.2334	0.1852	0.1842	0.1818
1300	0.3390	0.3353	0.3264	0.2410	0.2393	0.2350	0.1865	0.1855	0.1831
1310	0.3411	0.3373	0.3283	0.2426	0.2408	0.2365	0.1878	0.1868	0.1843
1320	0.3432	0.3394	0.3302	0.2442	0.2424	0.2380	0.1891	0.1881	0.1855
1330	0.3452	0.3414	0.3321	0.2458	0.2440	0.2396	0.1904	0.1894	0.1868
1340	0.3473	0.3434	0.3340	0.2474	0.2456	0.2411	0.1917	0.1906	0.1880

Continued on next page

B_1/Hz	$\alpha^{10}_{0.05}$	$\alpha^{10}_{0.1}$	$\alpha^{10}_{0.2}$	$\alpha^{15}_{0.05}$	$\alpha^{15}_{0.1}$	$\alpha^{15}_{0.2}$	$\alpha^{20}_{0.05}$	$\alpha^{20}_{0.1}$	$\alpha^{20}_{0.2}$
1350	0.3494	0.3454	0.3359	0.2490	0.2472	0.2426	0.1930	0.1919	0.1893
1360	0.3514	0.3474	0.3378	0.2506	0.2487	0.2441	0.1943	0.1932	0.1905
1370	0.3535	0.3494	0.3397	0.2522	0.2503	0.2456	0.1956	0.1945	0.1917
1380	0.3555	0.3514	0.3416	0.2538	0.2519	0.2471	0.1969	0.1957	0.1930
1390	0.3576	0.3534	0.3434	0.2554	0.2534	0.2486	0.1981	0.1970	0.1942
1400	0.3596	0.3554	0.3453	0.2570	0.2550	0.2501	0.1994	0.1983	0.1954
1410	0.3616	0.3574	0.3471	0.2586	0.2566	0.2516	0.2007	0.1995	0.1966
1420	0.3637	0.3593	0.3490	0.2602	0.2581	0.2531	0.2020	0.2008	0.1979
1430	0.3657	0.3613	0.3508	0.2617	0.2597	0.2546	0.2033	0.2021	0.1991
1440	0.3677	0.3632	0.3526	0.2633	0.2612	0.2560	0.2045	0.2033	0.2003
1450	0.3697	0.3652	0.3544	0.2649	0.2627	0.2575	0.2058	0.2046	0.2015
1460	0.3717	0.3671	0.3563	0.2664	0.2643	0.2590	0.2071	0.2058	0.2027
1470	0.3736	0.3690	0.3581	0.2680	0.2658	0.2604	0.2083	0.2071	0.2039
1480	0.3756	0.3710	0.3599	0.2696	0.2673	0.2619	0.2096	0.2083	0.2052
1490	0.3776	0.3729	0.3616	0.2711	0.2689	0.2634	0.2109	0.2096	0.2064
1500	0.3795	0.3748	0.3634	0.2727	0.2704	0.2648	0.2121	0.2108	0.2076
1510	0.3815	0.3767	0.3652	0.2742	0.2719	0.2663	0.2134	0.2121	0.2088
1520	0.3834	0.3786	0.3670	0.2758	0.2734	0.2677	0.2146	0.2133	0.2100
1530	0.3854	0.3805	0.3687	0.2773	0.2749	0.2692	0.2159	0.2145	0.2112
1540	0.3873	0.3823	0.3705	0.2788	0.2764	0.2706	0.2172	0.2158	0.2124
1550	0.3892	0.3842	0.3722	0.2804	0.2779	0.2720	0.2184	0.2170	0.2135
1560	0.3912	0.3861	0.3739	0.2819	0.2794	0.2735	0.2197	0.2182	0.2147
1570	0.3931	0.3879	0.3757	0.2834	0.2809	0.2749	0.2209	0.2195	0.2159
1580	0.3950	0.3898	0.3774	0.2849	0.2824	0.2763	0.2221	0.2207	0.2171
1590	0.3969	0.3916	0.3791	0.2865	0.2839	0.2777	0.2234	0.2219	0.2183
1600	0.3988	0.3935	0.3808	0.2880	0.2854	0.2792	0.2246	0.2231	0.2195
1610	0.4007	0.3953	0.3825	0.2895	0.2869	0.2806	0.2259	0.2244	0.2206
1620	0.4025	0.3971	0.3842	0.2910	0.2884	0.2820	0.2271	0.2256	0.2218
1630	0.4044	0.3989	0.3859	0.2925	0.2899	0.2834	0.2283	0.2268	0.2230
1640	0.4063	0.4007	0.3876	0.2940	0.2913	0.2848	0.2296	0.2280	0.2242
1650	0.4081	0.4025	0.3893	0.2955	0.2928	0.2862	0.2308	0.2292	0.2253
1660	0.4100	0.4043	0.3909	0.2970	0.2943	0.2876	0.2320	0.2304	0.2265
1670	0.4118	0.4061	0.3926	0.2985	0.2957	0.2890	0.2333	0.2316	0.2277
1680	0.4137	0.4079	0.3942	0.3000	0.2972	0.2903	0.2345	0.2329	0.2288
1690	0.4155	0.4097	0.3959	0.3015	0.2986	0.2917	0.2357	0.2341	0.2300
1700	0.4173	0.4114	0.3975	0.3030	0.3001	0.2931	0.2369	0.2353	0.2311
1710	0.4192	0.4132	0.3991	0.3044	0.3015	0.2945	0.2382	0.2365	0.2323
1720	0.4210	0.4150	0.4008	0.3059	0.3030	0.2959	0.2394	0.2377	0.2334
1730	0.4228	0.4167	0.4024	0.3074	0.3044	0.2972	0.2406	0.2389	0.2346
1740	0.4246	0.4185	0.4040	0.3089	0.3059	0.2986	0.2418	0.2400	0.2357
1750	0.4264	0.4202	0.4056	0.3103	0.3073	0.2999	0.2430	0.2412	0.2369
1760	0.4282	0.4219	0.4072	0.3118	0.3087	0.3013	0.2442	0.2424	0.2380

Continued on next page

B_1/Hz	$\alpha_{0.05}^{10}$	$\alpha_{0.1}^{10}$	$\alpha_{0.2}^{10}$	$\alpha_{0.05}^{15}$	$\alpha_{0.1}^{15}$	$\alpha_{0.2}^{15}$	$\alpha_{0.05}^{20}$	$\alpha_{0.1}^{20}$	$\alpha_{0.2}^{20}$
1770	0.4299	0.4236	0.4088	0.3133	0.3102	0.3027	0.2454	0.2436	0.2392
1780	0.4317	0.4254	0.4104	0.3147	0.3116	0.3040	0.2466	0.2448	0.2403
1790	0.4335	0.4271	0.4120	0.3162	0.3130	0.3053	0.2478	0.2460	0.2415
1800	0.4352	0.4288	0.4135	0.3176	0.3144	0.3067	0.2490	0.2472	0.2426
1810	0.4370	0.4305	0.4151	0.3191	0.3158	0.3080	0.2502	0.2484	0.2437
1820	0.4387	0.4322	0.4166	0.3205	0.3173	0.3094	0.2514	0.2495	0.2449
1830	0.4405	0.4338	0.4182	0.3220	0.3187	0.3107	0.2526	0.2507	0.2460
1840	0.4422	0.4355	0.4197	0.3234	0.3201	0.3120	0.2538	0.2519	0.2471
1850	0.4440	0.4372	0.4213	0.3248	0.3215	0.3133	0.2550	0.2531	0.2482
1860	0.4457	0.4388	0.4228	0.3263	0.3229	0.3147	0.2562	0.2542	0.2494
1870	0.4474	0.4405	0.4243	0.3277	0.3243	0.3160	0.2574	0.2554	0.2505
1880	0.4491	0.4422	0.4259	0.3291	0.3257	0.3173	0.2586	0.2566	0.2516
1890	0.4508	0.4438	0.4274	0.3305	0.3270	0.3186	0.2598	0.2577	0.2527
1900	0.4525	0.4454	0.4289	0.3319	0.3284	0.3199	0.2609	0.2589	0.2538
1910	0.4542	0.4471	0.4304	0.3334	0.3298	0.3212	0.2621	0.2600	0.2549
1920	0.4559	0.4487	0.4319	0.3348	0.3312	0.3225	0.2633	0.2612	0.2560
1930	0.4576	0.4503	0.4334	0.3362	0.3326	0.3238	0.2645	0.2624	0.2571
1940	0.4592	0.4519	0.4349	0.3376	0.3339	0.3251	0.2657	0.2635	0.2582
1950	0.4609	0.4535	0.4363	0.3390	0.3353	0.3264	0.2668	0.2647	0.2593
1960	0.4626	0.4551	0.4378	0.3404	0.3367	0.3277	0.2680	0.2658	0.2604
1970	0.4642	0.4567	0.4393	0.3418	0.3380	0.3289	0.2692	0.2670	0.2615
1980	0.4659	0.4583	0.4407	0.3432	0.3394	0.3302	0.2703	0.2681	0.2626
1990	0.4675	0.4599	0.4422	0.3446	0.3407	0.3315	0.2715	0.2692	0.2637
2000	0.4692	0.4615	0.4436	0.3459	0.3421	0.3328	0.2727	0.2704	0.2648
2010	0.4708	0.4631	0.4451	0.3473	0.3434	0.3340	0.2738	0.2715	0.2659
2020	0.4724	0.4646	0.4465	0.3487	0.3448	0.3353	0.2750	0.2727	0.2670
2030	0.4740	0.4662	0.4479	0.3501	0.3461	0.3366	0.2761	0.2738	0.2681
2040	0.4756	0.4677	0.4493	0.3514	0.3474	0.3378	0.2773	0.2749	0.2692
2050	0.4772	0.4693	0.4508	0.3528	0.3488	0.3391	0.2784	0.2761	0.2702
2060	0.4788	0.4708	0.4522	0.3542	0.3501	0.3403	0.2796	0.2772	0.2713
2070	0.4804	0.4724	0.4536	0.3555	0.3514	0.3416	0.2807	0.2783	0.2724
2080	0.4820	0.4739	0.4550	0.3569	0.3528	0.3428	0.2819	0.2794	0.2735
2090	0.4836	0.4754	0.4564	0.3583	0.3541	0.3440	0.2830	0.2806	0.2745
2100	0.4852	0.4769	0.4578	0.3596	0.3554	0.3453	0.2842	0.2817	0.2756
2110	0.4868	0.4784	0.4591	0.3610	0.3567	0.3465	0.2853	0.2828	0.2767
2120	0.4883	0.4799	0.4605	0.3623	0.3580	0.3477	0.2865	0.2839	0.2777
2130	0.4899	0.4814	0.4619	0.3637	0.3593	0.3490	0.2876	0.2850	0.2788
2140	0.4915	0.4829	0.4633	0.3650	0.3606	0.3502	0.2887	0.2862	0.2799
2150	0.4930	0.4844	0.4646	0.3663	0.3619	0.3514	0.2899	0.2873	0.2809
2160	0.4945	0.4859	0.4660	0.3677	0.3632	0.3526	0.2910	0.2884	0.2820
2170	0.4961	0.4874	0.4673	0.3690	0.3645	0.3538	0.2921	0.2895	0.2830
2180	0.4976	0.4889	0.4687	0.3703	0.3658	0.3550	0.2933	0.2906	0.2841

Continued on next page

B_1/Hz	$\alpha^{10}_{0.05}$	$\alpha^{10}_{0.1}$	$\alpha^{10}_{0.2}$	$\alpha^{15}_{0.05}$	$\alpha^{15}_{0.1}$	$\alpha^{15}_{0.2}$	$\alpha^{20}_{0.05}$	$\alpha^{20}_{0.1}$	$\alpha^{20}_{0.2}$
2190	0.4991	0.4903	0.4700	0.3717	0.3671	0.3563	0.2944	0.2917	0.2851
2200	0.5007	0.4918	0.4713	0.3730	0.3684	0.3575	0.2955	0.2928	0.2862
2210	0.5022	0.4932	0.4727	0.3743	0.3697	0.3587	0.2966	0.2939	0.2872
2220	0.5037	0.4947	0.4740	0.3756	0.3710	0.3599	0.2978	0.2950	0.2883
2230	0.5052	0.4961	0.4753	0.3769	0.3722	0.3610	0.2989	0.2961	0.2893
2240	0.5067	0.4976	0.4766	0.3782	0.3735	0.3622	0.3000	0.2972	0.2903
2250	0.5082	0.4990	0.4779	0.3795	0.3748	0.3634	0.3011	0.2983	0.2914
2260	0.5097	0.5004	0.4792	0.3808	0.3761	0.3646	0.3022	0.2994	0.2924
2270	0.5111	0.5018	0.4805	0.3821	0.3773	0.3658	0.3033	0.3005	0.2934
2280	0.5126	0.5033	0.4818	0.3834	0.3786	0.3670	0.3044	0.3015	0.2945
2290	0.5141	0.5047	0.4831	0.3847	0.3798	0.3681	0.3056	0.3026	0.2955
2300	0.5156	0.5061	0.4843	0.3860	0.3811	0.3693	0.3067	0.3037	0.2965
2310	0.5170	0.5075	0.4856	0.3873	0.3823	0.3705	0.3078	0.3048	0.2976
2320	0.5185	0.5089	0.4869	0.3886	0.3836	0.3716	0.3089	0.3059	0.2986
2330	0.5199	0.5103	0.4881	0.3899	0.3848	0.3728	0.3100	0.3069	0.2996
2340	0.5214	0.5116	0.4894	0.3912	0.3861	0.3739	0.3111	0.3080	0.3006
2350	0.5228	0.5130	0.4907	0.3924	0.3873	0.3751	0.3122	0.3091	0.3016
2360	0.5242	0.5144	0.4919	0.3937	0.3886	0.3762	0.3133	0.3102	0.3027
2370	0.5257	0.5158	0.4931	0.3950	0.3898	0.3774	0.3144	0.3112	0.3037
2380	0.5271	0.5171	0.4944	0.3963	0.3910	0.3785	0.3154	0.3123	0.3047
2390	0.5285	0.5185	0.4956	0.3975	0.3922	0.3797	0.3165	0.3134	0.3057
2400	0.5299	0.5198	0.4968	0.3988	0.3935	0.3808	0.3176	0.3144	0.3067
2410	0.5313	0.5212	0.4981	0.4000	0.3947	0.3820	0.3187	0.3155	0.3077
2420	0.5327	0.5225	0.4993	0.4013	0.3959	0.3831	0.3198	0.3166	0.3087
2430	0.5341	0.5238	0.5005	0.4025	0.3971	0.3842	0.3209	0.3176	0.3097
2440	0.5355	0.5252	0.5017	0.4038	0.3983	0.3853	0.3220	0.3187	0.3107
2450	0.5369	0.5265	0.5029	0.4050	0.3995	0.3865	0.3230	0.3197	0.3117
2460	0.5383	0.5278	0.5041	0.4063	0.4007	0.3876	0.3241	0.3208	0.3127
2470	0.5396	0.5291	0.5053	0.4075	0.4019	0.3887	0.3252	0.3218	0.3137
2480	0.5410	0.5304	0.5065	0.4088	0.4031	0.3898	0.3263	0.3229	0.3147
2490	0.5424	0.5317	0.5077	0.4100	0.4043	0.3909	0.3273	0.3239	0.3156
2500	0.5437	0.5330	0.5088	0.4112	0.4055	0.3920	0.3284	0.3250	0.3166
2510	0.5451	0.5343	0.5100	0.4125	0.4067	0.3931	0.3295	0.3260	0.3176
2520	0.5464	0.5356	0.5112	0.4137	0.4079	0.3942	0.3305	0.3270	0.3186
2530	0.5478	0.5369	0.5123	0.4149	0.4091	0.3953	0.3316	0.3281	0.3196
2540	0.5491	0.5382	0.5135	0.4161	0.4103	0.3964	0.3326	0.3291	0.3206
2550	0.5505	0.5395	0.5147	0.4173	0.4114	0.3975	0.3337	0.3301	0.3215
2560	0.5518	0.5407	0.5158	0.4186	0.4126	0.3986	0.3348	0.3312	0.3225
2570	0.5531	0.5420	0.5170	0.4198	0.4138	0.3997	0.3358	0.3322	0.3235
2580	0.5544	0.5433	0.5181	0.4210	0.4150	0.4008	0.3369	0.3332	0.3244
2590	0.5557	0.5445	0.5192	0.4222	0.4161	0.4018	0.3379	0.3343	0.3254
2600	0.5571	0.5458	0.5204	0.4234	0.4173	0.4029	0.3390	0.3353	0.3264

Continued on next page

B_1/Hz	$\alpha^{10}_{0.05}$	$\alpha^{10}_{0.1}$	$\alpha^{10}_{0.2}$	$\alpha^{15}_{0.05}$	$\alpha^{15}_{0.1}$	$\alpha^{15}_{0.2}$	$\alpha^{20}_{0.05}$	$\alpha^{20}_{0.1}$	$\alpha^{20}_{0.2}$
2610	0.5584	0.5470	0.5215	0.4246	0.4185	0.4040	0.3400	0.3363	0.3273
2620	0.5597	0.5483	0.5226	0.4258	0.4196	0.4051	0.3411	0.3373	0.3283
2630	0.5609	0.5495	0.5237	0.4270	0.4208	0.4061	0.3421	0.3384	0.3293
2640	0.5622	0.5507	0.5248	0.4282	0.4219	0.4072	0.3432	0.3394	0.3302
2650	0.5635	0.5520	0.5260	0.4293	0.4231	0.4083	0.3442	0.3404	0.3312
2660	0.5648	0.5532	0.5271	0.4305	0.4242	0.4093	0.3452	0.3414	0.3321
2670	0.5661	0.5544	0.5282	0.4317	0.4254	0.4104	0.3463	0.3424	0.3331
2680	0.5673	0.5556	0.5293	0.4329	0.4265	0.4114	0.3473	0.3434	0.3340
2690	0.5686	0.5568	0.5303	0.4341	0.4276	0.4125	0.3484	0.3444	0.3350
2700	0.5699	0.5580	0.5314	0.4352	0.4288	0.4135	0.3494	0.3454	0.3359
2710	0.5711	0.5592	0.5325	0.4364	0.4299	0.4146	0.3504	0.3464	0.3369
2720	0.5724	0.5604	0.5336	0.4376	0.4310	0.4156	0.3514	0.3474	0.3378
2730	0.5736	0.5616	0.5347	0.4387	0.4322	0.4166	0.3525	0.3484	0.3388
2740	0.5749	0.5628	0.5357	0.4399	0.4333	0.4177	0.3535	0.3494	0.3397
2750	0.5761	0.5640	0.5368	0.4411	0.4344	0.4187	0.3545	0.3504	0.3406
2760	0.5773	0.5651	0.5379	0.4422	0.4355	0.4197	0.3555	0.3514	0.3416
2770	0.5786	0.5663	0.5389	0.4434	0.4366	0.4208	0.3566	0.3524	0.3425
2780	0.5798	0.5675	0.5400	0.4445	0.4377	0.4218	0.3576	0.3534	0.3434
2790	0.5810	0.5686	0.5410	0.4457	0.4388	0.4228	0.3586	0.3544	0.3444
2800	0.5822	0.5698	0.5421	0.4468	0.4400	0.4238	0.3596	0.3554	0.3453
2810	0.5834	0.5709	0.5431	0.4480	0.4411	0.4249	0.3606	0.3564	0.3462
2820	0.5846	0.5721	0.5442	0.4491	0.4422	0.4259	0.3616	0.3574	0.3471
2830	0.5858	0.5732	0.5452	0.4502	0.4433	0.4269	0.3626	0.3584	0.3481
2840	0.5870	0.5744	0.5462	0.4514	0.4443	0.4279	0.3637	0.3593	0.3490
2850	0.5882	0.5755	0.5472	0.4525	0.4454	0.4289	0.3647	0.3603	0.3499
2860	0.5894	0.5766	0.5483	0.4536	0.4465	0.4299	0.3657	0.3613	0.3508
2870	0.5906	0.5778	0.5493	0.4548	0.4476	0.4309	0.3667	0.3623	0.3517
2880	0.5918	0.5789	0.5503	0.4559	0.4487	0.4319	0.3677	0.3632	0.3526
2890	0.5929	0.5800	0.5513	0.4570	0.4498	0.4329	0.3687	0.3642	0.3535
2900	0.5941	0.5811	0.5523	0.4581	0.4509	0.4339	0.3697	0.3652	0.3544
2910	0.5953	0.5822	0.5533	0.4592	0.4519	0.4349	0.3707	0.3662	0.3553
2920	0.5964	0.5833	0.5543	0.4604	0.4530	0.4358	0.3717	0.3671	0.3563
2930	0.5976	0.5844	0.5553	0.4615	0.4541	0.4368	0.3726	0.3681	0.3572
2940	0.5988	0.5855	0.5563	0.4626	0.4551	0.4378	0.3736	0.3690	0.3581
2950	0.5999	0.5866	0.5573	0.4637	0.4562	0.4388	0.3746	0.3700	0.3590
2960	0.6010	0.5877	0.5583	0.4648	0.4573	0.4398	0.3756	0.3710	0.3599
2970	0.6022	0.5888	0.5592	0.4659	0.4583	0.4407	0.3766	0.3719	0.3607
2980	0.6033	0.5899	0.5602	0.4670	0.4594	0.4417	0.3776	0.3729	0.3616
2990	0.6044	0.5910	0.5612	0.4681	0.4604	0.4427	0.3786	0.3738	0.3625
3000	0.6056	0.5920	0.5621	0.4692	0.4615	0.4436	0.3795	0.3748	0.3634
3010	0.6067	0.5931	0.5631	0.4702	0.4625	0.4446	0.3805	0.3757	0.3643
3020	0.6078	0.5942	0.5641	0.4713	0.4636	0.4455	0.3815	0.3767	0.3652

Continued on next page

B_1/Hz	$\alpha^{10}_{0.05}$	$\alpha^{10}_{0.1}$	$\alpha^{10}_{0.2}$	$\alpha^{15}_{0.05}$	$\alpha^{15}_{0.1}$	$\alpha^{15}_{0.2}$	$\alpha^{20}_{0.05}$	$\alpha^{20}_{0.1}$	$\alpha^{20}_{0.2}$
3030	0.6089	0.5952	0.5650	0.4724	0.4646	0.4465	0.3825	0.3776	0.3661
3040	0.6100	0.5963	0.5660	0.4735	0.4657	0.4475	0.3834	0.3786	0.3670
3050	0.6111	0.5973	0.5669	0.4746	0.4667	0.4484	0.3844	0.3795	0.3678
3060	0.6122	0.5984	0.5679	0.4756	0.4677	0.4493	0.3854	0.3805	0.3687
3070	0.6133	0.5994	0.5688	0.4767	0.4688	0.4503	0.3864	0.3814	0.3696
3080	0.6144	0.6005	0.5697	0.4778	0.4698	0.4512	0.3873	0.3823	0.3705
3090	0.6155	0.6015	0.5707	0.4788	0.4708	0.4522	0.3883	0.3833	0.3713
3100	0.6166	0.6025	0.5716	0.4799	0.4718	0.4531	0.3892	0.3842	0.3722
3110	0.6177	0.6035	0.5725	0.4810	0.4729	0.4540	0.3902	0.3851	0.3731
3120	0.6188	0.6046	0.5734	0.4820	0.4739	0.4550	0.3912	0.3861	0.3739
3130	0.6198	0.6056	0.5744	0.4831	0.4749	0.4559	0.3921	0.3870	0.3748
3140	0.6209	0.6066	0.5753	0.4841	0.4759	0.4568	0.3931	0.3879	0.3757
3150	0.6220	0.6076	0.5762	0.4852	0.4769	0.4578	0.3940	0.3889	0.3765
3160	0.6230	0.6086	0.5771	0.4862	0.4779	0.4587	0.3950	0.3898	0.3774
3170	0.6241	0.6096	0.5780	0.4873	0.4789	0.4596	0.3959	0.3907	0.3783
3180	0.6251	0.6106	0.5789	0.4883	0.4799	0.4605	0.3969	0.3916	0.3791
3190	0.6262	0.6116	0.5798	0.4894	0.4809	0.4614	0.3978	0.3925	0.3800
3200	0.6272	0.6126	0.5807	0.4904	0.4819	0.4623	0.3988	0.3935	0.3808
3210	0.6283	0.6136	0.5816	0.4915	0.4829	0.4633	0.3997	0.3944	0.3817
3220	0.6293	0.6146	0.5825	0.4925	0.4839	0.4642	0.4007	0.3953	0.3825
3230	0.6303	0.6156	0.5834	0.4935	0.4849	0.4651	0.4016	0.3962	0.3834
3240	0.6314	0.6165	0.5842	0.4945	0.4859	0.4660	0.4025	0.3971	0.3842
3250	0.6324	0.6175	0.5851	0.4956	0.4869	0.4669	0.4035	0.3980	0.3851
3260	0.6334	0.6185	0.5860	0.4966	0.4879	0.4678	0.4044	0.3989	0.3859
3270	0.6344	0.6195	0.5869	0.4976	0.4889	0.4687	0.4053	0.3998	0.3867
3280	0.6354	0.6204	0.5877	0.4986	0.4898	0.4696	0.4063	0.4007	0.3876
3290	0.6365	0.6214	0.5886	0.4996	0.4908	0.4704	0.4072	0.4016	0.3884
3300	0.6375	0.6223	0.5895	0.5007	0.4918	0.4713	0.4081	0.4025	0.3893
3310	0.6385	0.6233	0.5903	0.5017	0.4927	0.4722	0.4091	0.4034	0.3901
3320	0.6395	0.6242	0.5912	0.5027	0.4937	0.4731	0.4100	0.4043	0.3909
3330	0.6405	0.6252	0.5920	0.5037	0.4947	0.4740	0.4109	0.4052	0.3917
3340	0.6414	0.6261	0.5929	0.5047	0.4956	0.4749	0.4118	0.4061	0.3926
3350	0.6424	0.6271	0.5937	0.5057	0.4966	0.4757	0.4128	0.4070	0.3934
3360	0.6434	0.6280	0.5946	0.5067	0.4976	0.4766	0.4137	0.4079	0.3942
3370	0.6444	0.6289	0.5954	0.5077	0.4985	0.4775	0.4146	0.4088	0.3951
3380	0.6454	0.6299	0.5962	0.5087	0.4995	0.4783	0.4155	0.4097	0.3959
3390	0.6463	0.6308	0.5971	0.5097	0.5004	0.4792	0.4164	0.4106	0.3967
3400	0.6473	0.6317	0.5979	0.5107	0.5014	0.4801	0.4173	0.4114	0.3975
3410	0.6483	0.6326	0.5987	0.5116	0.5023	0.4809	0.4182	0.4123	0.3983
3420	0.6492	0.6335	0.5996	0.5126	0.5033	0.4818	0.4192	0.4132	0.3991
3430	0.6502	0.6344	0.6004	0.5136	0.5042	0.4826	0.4201	0.4141	0.4000
3440	0.6512	0.6353	0.6012	0.5146	0.5051	0.4835	0.4210	0.4150	0.4008

Continued on next page

B_1/Hz	$\alpha^{10}_{0.05}$	$\alpha^{10}_{0.1}$	$\alpha^{10}_{0.2}$	$\alpha^{15}_{0.05}$	$\alpha^{15}_{0.1}$	$\alpha^{15}_{0.2}$	$\alpha^{20}_{0.05}$	$\alpha^{20}_{0.1}$	$\alpha^{20}_{0.2}$
3450	0.6521	0.6362	0.6020	0.5156	0.5061	0.4843	0.4219	0.4158	0.4016
3460	0.6531	0.6371	0.6028	0.5165	0.5070	0.4852	0.4228	0.4167	0.4024
3470	0.6540	0.6380	0.6036	0.5175	0.5079	0.4860	0.4237	0.4176	0.4032
3480	0.6549	0.6389	0.6044	0.5185	0.5089	0.4869	0.4246	0.4185	0.4040
3490	0.6559	0.6398	0.6052	0.5194	0.5098	0.4877	0.4255	0.4193	0.4048
3500	0.6568	0.6407	0.6060	0.5204	0.5107	0.4886	0.4264	0.4202	0.4056
3510	0.6577	0.6416	0.6068	0.5214	0.5116	0.4894	0.4273	0.4211	0.4064
3520	0.6587	0.6425	0.6076	0.5223	0.5126	0.4902	0.4282	0.4219	0.4072
3530	0.6596	0.6433	0.6084	0.5233	0.5135	0.4911	0.4290	0.4228	0.4080
3540	0.6605	0.6442	0.6092	0.5242	0.5144	0.4919	0.4299	0.4236	0.4088
3550	0.6614	0.6451	0.6100	0.5252	0.5153	0.4927	0.4308	0.4245	0.4096
3560	0.6623	0.6460	0.6108	0.5261	0.5162	0.4936	0.4317	0.4254	0.4104
3570	0.6633	0.6468	0.6116	0.5271	0.5171	0.4944	0.4326	0.4262	0.4112
3580	0.6642	0.6477	0.6123	0.5280	0.5180	0.4952	0.4335	0.4271	0.4120
3590	0.6651	0.6485	0.6131	0.5290	0.5189	0.4960	0.4344	0.4279	0.4127
3600	0.6660	0.6494	0.6139	0.5299	0.5198	0.4968	0.4352	0.4288	0.4135
3610	0.6669	0.6502	0.6147	0.5308	0.5207	0.4977	0.4361	0.4296	0.4143
3620	0.6677	0.6511	0.6154	0.5318	0.5216	0.4985	0.4370	0.4305	0.4151
3630	0.6686	0.6519	0.6162	0.5327	0.5225	0.4993	0.4379	0.4313	0.4159
3640	0.6695	0.6528	0.6169	0.5336	0.5234	0.5001	0.4387	0.4322	0.4166
3650	0.6704	0.6536	0.6177	0.5346	0.5243	0.5009	0.4396	0.4330	0.4174
3660	0.6713	0.6544	0.6185	0.5355	0.5252	0.5017	0.4405	0.4338	0.4182
3670	0.6722	0.6553	0.6192	0.5364	0.5261	0.5025	0.4414	0.4347	0.4190
3680	0.6730	0.6561	0.6200	0.5373	0.5269	0.5033	0.4422	0.4355	0.4197
3690	0.6739	0.6569	0.6207	0.5383	0.5278	0.5041	0.4431	0.4363	0.4205
3700	0.6748	0.6578	0.6214	0.5392	0.5287	0.5049	0.4440	0.4372	0.4213
3710	0.6756	0.6586	0.6222	0.5401	0.5296	0.5057	0.4448	0.4380	0.4221
3720	0.6765	0.6594	0.6229	0.5410	0.5304	0.5065	0.4457	0.4388	0.4228
3730	0.6774	0.6602	0.6237	0.5419	0.5313	0.5073	0.4465	0.4397	0.4236
3740	0.6782	0.6610	0.6244	0.5428	0.5322	0.5081	0.4474	0.4405	0.4243
3750	0.6791	0.6618	0.6251	0.5437	0.5330	0.5088	0.4482	0.4413	0.4251
3760	0.6799	0.6626	0.6258	0.5446	0.5339	0.5096	0.4491	0.4422	0.4259
3770	0.6807	0.6634	0.6266	0.5455	0.5348	0.5104	0.4500	0.4430	0.4266
3780	0.6816	0.6642	0.6273	0.5464	0.5356	0.5112	0.4508	0.4438	0.4274
3790	0.6824	0.6650	0.6280	0.5473	0.5365	0.5120	0.4517	0.4446	0.4281
3800	0.6833	0.6658	0.6287	0.5482	0.5373	0.5127	0.4525	0.4454	0.4289
3810	0.6841	0.6666	0.6294	0.5491	0.5382	0.5135	0.4534	0.4463	0.4296
3820	0.6849	0.6674	0.6302	0.5500	0.5390	0.5143	0.4542	0.4471	0.4304
3830	0.6858	0.6682	0.6309	0.5509	0.5399	0.5150	0.4550	0.4479	0.4311
3840	0.6866	0.6690	0.6316	0.5518	0.5407	0.5158	0.4559	0.4487	0.4319
3850	0.6874	0.6697	0.6323	0.5527	0.5416	0.5166	0.4567	0.4495	0.4326
3860	0.6882	0.6705	0.6330	0.5535	0.5424	0.5173	0.4576	0.4503	0.4334

Continued on next page

B_1/Hz	$\alpha^{10}_{0.05}$	$\alpha^{10}_{0.1}$	$\alpha^{10}_{0.2}$	$\alpha^{15}_{0.05}$	$\alpha^{15}_{0.1}$	$\alpha^{15}_{0.2}$	$\alpha^{20}_{0.05}$	$\alpha^{20}_{0.1}$	$\alpha^{20}_{0.2}$
3870	0.6890	0.6713	0.6337	0.5544	0.5433	0.5181	0.4584	0.4511	0.4341
3880	0.6898	0.6721	0.6344	0.5553	0.5441	0.5189	0.4592	0.4519	0.4349
3890	0.6906	0.6728	0.6351	0.5562	0.5449	0.5196	0.4601	0.4527	0.4356
3900	0.6915	0.6736	0.6358	0.5571	0.5458	0.5204	0.4609	0.4535	0.4363
3910	0.6923	0.6743	0.6365	0.5579	0.5466	0.5211	0.4617	0.4543	0.4371
3920	0.6931	0.6751	0.6371	0.5588	0.5474	0.5219	0.4626	0.4551	0.4378
3930	0.6938	0.6759	0.6378	0.5597	0.5483	0.5226	0.4634	0.4559	0.4385
3940	0.6946	0.6766	0.6385	0.5605	0.5491	0.5234	0.4642	0.4567	0.4393
3950	0.6954	0.6774	0.6392	0.5614	0.5499	0.5241	0.4650	0.4575	0.4400
3960	0.6962	0.6781	0.6399	0.5622	0.5507	0.5248	0.4659	0.4583	0.4407
3970	0.6970	0.6789	0.6406	0.5631	0.5515	0.5256	0.4667	0.4591	0.4415
3980	0.6978	0.6796	0.6412	0.5639	0.5524	0.5263	0.4675	0.4599	0.4422
3990	0.6986	0.6803	0.6419	0.5648	0.5532	0.5271	0.4683	0.4607	0.4429
4000	0.6993	0.6811	0.6426	0.5657	0.5540	0.5278	0.4692	0.4615	0.4436
4010	0.7001	0.6818	0.6432	0.5665	0.5548	0.5285	0.4700	0.4623	0.4443
4020	0.7009	0.6825	0.6439	0.5673	0.5556	0.5293	0.4708	0.4631	0.4451
4030	0.7017	0.6833	0.6446	0.5682	0.5564	0.5300	0.4716	0.4638	0.4458
4040	0.7024	0.6840	0.6452	0.5690	0.5572	0.5307	0.4724	0.4646	0.4465
4050	0.7032	0.6847	0.6459	0.5699	0.5580	0.5314	0.4732	0.4654	0.4472
4060	0.7039	0.6854	0.6465	0.5707	0.5588	0.5322	0.4740	0.4662	0.4479
4070	0.7047	0.6862	0.6472	0.5715	0.5596	0.5329	0.4748	0.4670	0.4486
4080	0.7054	0.6869	0.6478	0.5724	0.5604	0.5336	0.4756	0.4677	0.4493
4090	0.7062	0.6876	0.6485	0.5732	0.5612	0.5343	0.4764	0.4685	0.4501
4100	0.7069	0.6883	0.6491	0.5740	0.5620	0.5350	0.4772	0.4693	0.4508
4110	0.7077	0.6890	0.6498	0.5749	0.5628	0.5357	0.4780	0.4700	0.4515
4120	0.7084	0.6897	0.6504	0.5757	0.5636	0.5365	0.4788	0.4708	0.4522
4130	0.7092	0.6904	0.6511	0.5765	0.5644	0.5372	0.4796	0.4716	0.4529
4140	0.7099	0.6911	0.6517	0.5773	0.5651	0.5379	0.4804	0.4724	0.4536
4150	0.7106	0.6918	0.6523	0.5782	0.5659	0.5386	0.4812	0.4731	0.4543
4160	0.7114	0.6925	0.6530	0.5790	0.5667	0.5393	0.4820	0.4739	0.4550
4170	0.7121	0.6932	0.6536	0.5798	0.5675	0.5400	0.4828	0.4746	0.4557
4180	0.7128	0.6939	0.6542	0.5806	0.5682	0.5407	0.4836	0.4754	0.4564
4190	0.7136	0.6946	0.6548	0.5814	0.5690	0.5414	0.4844	0.4762	0.4571
4200	0.7143	0.6953	0.6555	0.5822	0.5698	0.5421	0.4852	0.4769	0.4578
4210	0.7150	0.6959	0.6561	0.5830	0.5706	0.5428	0.4860	0.4777	0.4585
4220	0.7157	0.6966	0.6567	0.5838	0.5713	0.5435	0.4868	0.4784	0.4591
4230	0.7164	0.6973	0.6573	0.5846	0.5721	0.5442	0.4876	0.4792	0.4598
4240	0.7171	0.6980	0.6579	0.5854	0.5729	0.5448	0.4883	0.4799	0.4605
4250	0.7179	0.6987	0.6586	0.5862	0.5736	0.5455	0.4891	0.4807	0.4612
4260	0.7186	0.6993	0.6592	0.5870	0.5744	0.5462	0.4899	0.4814	0.4619
4270	0.7193	0.7000	0.6598	0.5878	0.5751	0.5469	0.4907	0.4822	0.4626
4280	0.7200	0.7007	0.6604	0.5886	0.5759	0.5476	0.4915	0.4829	0.4633

Continued on next page

B_1/Hz	$\alpha^{10}_{0.05}$	$\alpha^{10}_{0.1}$	$\alpha^{10}_{0.2}$	$\alpha^{15}_{0.05}$	$\alpha^{15}_{0.1}$	$\alpha^{15}_{0.2}$	$\alpha^{20}_{0.05}$	$\alpha^{20}_{0.1}$	$\alpha^{20}_{0.2}$
4290	0.7207	0.7013	0.6610	0.5894	0.5766	0.5483	0.4922	0.4837	0.4639
4300	0.7214	0.7020	0.6616	0.5902	0.5774	0.5489	0.4930	0.4844	0.4646
4310	0.7221	0.7026	0.6622	0.5910	0.5781	0.5496	0.4938	0.4852	0.4653
4320	0.7227	0.7033	0.6628	0.5918	0.5789	0.5503	0.4945	0.4859	0.4660
4330	0.7234	0.7040	0.6634	0.5926	0.5796	0.5510	0.4953	0.4866	0.4666
4340	0.7241	0.7046	0.6640	0.5933	0.5804	0.5516	0.4961	0.4874	0.4673
4350	0.7248	0.7053	0.6646	0.5941	0.5811	0.5523	0.4968	0.4881	0.4680
4360	0.7255	0.7059	0.6652	0.5949	0.5819	0.5530	0.4976	0.4889	0.4687
4370	0.7262	0.7065	0.6658	0.5957	0.5826	0.5536	0.4984	0.4896	0.4693
4380	0.7268	0.7072	0.6664	0.5964	0.5833	0.5543	0.4991	0.4903	0.4700
4390	0.7275	0.7078	0.6669	0.5972	0.5841	0.5550	0.4999	0.4910	0.4707
4400	0.7282	0.7085	0.6675	0.5980	0.5848	0.5556	0.5007	0.4918	0.4713
4410	0.7289	0.7091	0.6681	0.5988	0.5855	0.5563	0.5014	0.4925	0.4720
4420	0.7295	0.7097	0.6687	0.5995	0.5863	0.5569	0.5022	0.4932	0.4727
4430	0.7302	0.7104	0.6693	0.6003	0.5870	0.5576	0.5029	0.4940	0.4733
4440	0.7308	0.7110	0.6698	0.6010	0.5877	0.5583	0.5037	0.4947	0.4740
4450	0.7315	0.7116	0.6704	0.6018	0.5884	0.5589	0.5044	0.4954	0.4746
4460	0.7322	0.7123	0.6710	0.6026	0.5892	0.5596	0.5052	0.4961	0.4753
4470	0.7328	0.7129	0.6716	0.6033	0.5899	0.5602	0.5059	0.4968	0.4759
4480	0.7335	0.7135	0.6721	0.6041	0.5906	0.5608	0.5067	0.4976	0.4766
4490	0.7341	0.7141	0.6727	0.6048	0.5913	0.5615	0.5074	0.4983	0.4773
4500	0.7348	0.7147	0.6733	0.6056	0.5920	0.5621	0.5082	0.4990	0.4779
4510	0.7354	0.7153	0.6738	0.6063	0.5927	0.5628	0.5089	0.4997	0.4786
4520	0.7361	0.7160	0.6744	0.6071	0.5934	0.5634	0.5097	0.5004	0.4792
4530	0.7367	0.7166	0.6749	0.6078	0.5942	0.5641	0.5104	0.5011	0.4798
4540	0.7373	0.7172	0.6755	0.6086	0.5949	0.5647	0.5111	0.5018	0.4805
4550	0.7380	0.7178	0.6761	0.6093	0.5956	0.5653	0.5119	0.5025	0.4811
4560	0.7386	0.7184	0.6766	0.6100	0.5963	0.5660	0.5126	0.5033	0.4818
4570	0.7392	0.7190	0.6772	0.6108	0.5970	0.5666	0.5134	0.5040	0.4824
4580	0.7399	0.7196	0.6777	0.6115	0.5977	0.5672	0.5141	0.5047	0.4831
4590	0.7405	0.7202	0.6783	0.6122	0.5984	0.5679	0.5148	0.5054	0.4837
4600	0.7411	0.7208	0.6788	0.6130	0.5991	0.5685	0.5156	0.5061	0.4843
4610	0.7417	0.7214	0.6794	0.6137	0.5998	0.5691	0.5163	0.5068	0.4850
4620	0.7424	0.7220	0.6799	0.6144	0.6005	0.5697	0.5170	0.5075	0.4856
4630	0.7430	0.7226	0.6804	0.6152	0.6011	0.5704	0.5177	0.5082	0.4863
4640	0.7436	0.7231	0.6810	0.6159	0.6018	0.5710	0.5185	0.5089	0.4869
4650	0.7442	0.7237	0.6815	0.6166	0.6025	0.5716	0.5192	0.5096	0.4875
4660	0.7448	0.7243	0.6821	0.6173	0.6032	0.5722	0.5199	0.5103	0.4881
4670	0.7454	0.7249	0.6826	0.6180	0.6039	0.5728	0.5206	0.5109	0.4888
4680	0.7460	0.7255	0.6831	0.6188	0.6046	0.5734	0.5214	0.5116	0.4894
4690	0.7467	0.7260	0.6836	0.6195	0.6052	0.5741	0.5221	0.5123	0.4900
4700	0.7473	0.7266	0.6842	0.6202	0.6059	0.5747	0.5228	0.5130	0.4907

Continued on next page

B_1/Hz	$\alpha^{10}_{0.05}$	$\alpha^{10}_{0.1}$	$\alpha^{10}_{0.2}$	$\alpha^{15}_{0.05}$	$\alpha^{15}_{0.1}$	$\alpha^{15}_{0.2}$	$\alpha^{20}_{0.05}$	$\alpha^{20}_{0.1}$	$\alpha^{20}_{0.2}$
4710	0.7479	0.7272	0.6847	0.6209	0.6066	0.5753	0.5235	0.5137	0.4913
4720	0.7485	0.7278	0.6852	0.6216	0.6073	0.5759	0.5242	0.5144	0.4919
4730	0.7491	0.7283	0.6858	0.6223	0.6080	0.5765	0.5249	0.5151	0.4925
4740	0.7497	0.7289	0.6863	0.6230	0.6086	0.5771	0.5257	0.5158	0.4931
4750	0.7502	0.7295	0.6868	0.6237	0.6093	0.5777	0.5264	0.5164	0.4938
4760	0.7508	0.7300	0.6873	0.6244	0.6100	0.5783	0.5271	0.5171	0.4944
4770	0.7514	0.7306	0.6878	0.6251	0.6106	0.5789	0.5278	0.5178	0.4950
4780	0.7520	0.7312	0.6884	0.6258	0.6113	0.5795	0.5285	0.5185	0.4956
4790	0.7526	0.7317	0.6889	0.6265	0.6120	0.5801	0.5292	0.5191	0.4962
4800	0.7532	0.7323	0.6894	0.6272	0.6126	0.5807	0.5299	0.5198	0.4968
4810	0.7538	0.7328	0.6899	0.6279	0.6133	0.5813	0.5306	0.5205	0.4975
4820	0.7543	0.7334	0.6904	0.6286	0.6139	0.5819	0.5313	0.5212	0.4981
4830	0.7549	0.7339	0.6909	0.6293	0.6146	0.5825	0.5320	0.5218	0.4987
4840	0.7555	0.7345	0.6914	0.6300	0.6152	0.5831	0.5327	0.5225	0.4993
4850	0.7561	0.7350	0.6919	0.6307	0.6159	0.5837	0.5334	0.5232	0.4999
4860	0.7566	0.7356	0.6924	0.6314	0.6165	0.5842	0.5341	0.5238	0.5005
4870	0.7572	0.7361	0.6929	0.6321	0.6172	0.5848	0.5348	0.5245	0.5011
4880	0.7578	0.7366	0.6934	0.6327	0.6178	0.5854	0.5355	0.5252	0.5017
4890	0.7583	0.7372	0.6939	0.6334	0.6185	0.5860	0.5362	0.5258	0.5023
4900	0.7589	0.7377	0.6944	0.6341	0.6191	0.5866	0.5369	0.5265	0.5029
4910	0.7595	0.7383	0.6949	0.6348	0.6198	0.5872	0.5376	0.5272	0.5035
4920	0.7600	0.7388	0.6954	0.6354	0.6204	0.5877	0.5383	0.5278	0.5041
4930	0.7606	0.7393	0.6959	0.6361	0.6211	0.5883	0.5390	0.5285	0.5047
4940	0.7611	0.7399	0.6964	0.6368	0.6217	0.5889	0.5396	0.5291	0.5053
4950	0.7617	0.7404	0.6969	0.6375	0.6223	0.5895	0.5403	0.5298	0.5059
4960	0.7622	0.7409	0.6974	0.6381	0.6230	0.5900	0.5410	0.5304	0.5065
4970	0.7628	0.7414	0.6979	0.6388	0.6236	0.5906	0.5417	0.5311	0.5071
4980	0.7633	0.7420	0.6984	0.6395	0.6242	0.5912	0.5424	0.5317	0.5077
4990	0.7639	0.7425	0.6988	0.6401	0.6249	0.5917	0.5431	0.5324	0.5083
5000	0.7644	0.7430	0.6993	0.6408	0.6255	0.5923	0.5437	0.5330	0.5088

Table S2: Ubiquitin R_1, R_2, and steady-state nuclear Overhauser effect (NOE) ratios measured at 800 MHz and 30°C. Residue numbers, longitudinal relaxation rates (R_1), transverse relaxation rates (R_2), and NOE ratios ($\sigma\{^1\text{H}\}$) are shown in the first, second, third, and fourth column, respectively.

res.	R_1/s^{-1}	R_2/s^{-1}	$\sigma\{^1\text{H}\}$
2	1.711 ± 0.017	6.73 ± 0.07	0.775 ± 0.011
3	1.823 ± 0.036	6.59 ± 0.13	0.802 ± 0.011
4	1.829 ± 0.036	6.39 ± 0.13	0.812 ± 0.011
5	1.715 ± 0.035	5.84 ± 0.13	0.791 ± 0.011
6	1.794 ± 0.031	6.47 ± 0.13	0.791 ± 0.010
7	1.775 ± 0.022	6.43 ± 0.08	0.770 ± 0.010
8	1.827 ± 0.022	5.87 ± 0.06	0.703 ± 0.011
9	1.742 ± 0.024	6.06 ± 0.07	0.664 ± 0.010
10	1.711 ± 0.019	5.54 ± 0.06	0.670 ± 0.008
11	1.646 ± 0.017	5.57 ± 0.05	0.650 ± 0.008
12	1.658 ± 0.015	5.80 ± 0.05	0.722 ± 0.009
13	1.788 ± 0.031	6.25 ± 0.13	0.781 ± 0.012
14	1.701 ± 0.019	6.46 ± 0.07	0.792 ± 0.010
15	1.821 ± 0.032	6.08 ± 0.13	0.799 ± 0.010
16	1.601 ± 0.014	5.85 ± 0.06	0.761 ± 0.009
17	1.811 ± 0.019	6.34 ± 0.06	0.782 ± 0.009
18	1.662 ± 0.026	6.53 ± 0.11	0.773 ± 0.012
20	1.738 ± 0.029	6.24 ± 0.11	0.772 ± 0.011
21	1.937 ± 0.028	6.89 ± 0.10	0.812 ± 0.010
22	1.799 ± 0.028	6.14 ± 0.09	0.780 ± 0.012
23	1.881 ± 0.042	7.75 ± 0.16	0.813 ± 0.014
25	1.850 ± 0.033	9.96 ± 0.13	0.812 ± 0.012
26	1.838 ± 0.029	6.46 ± 0.10	0.802 ± 0.010
27	1.858 ± 0.041	6.89 ± 0.15	0.814 ± 0.014
28	1.903 ± 0.032	6.97 ± 0.12	0.806 ± 0.011
29	1.817 ± 0.034	6.45 ± 0.13	0.808 ± 0.011
30	1.825 ± 0.034	6.49 ± 0.13	0.806 ± 0.010
32	1.828 ± 0.030	6.46 ± 0.11	0.804 ± 0.011
33	1.758 ± 0.026	6.33 ± 0.11	0.776 ± 0.011
34	1.712 ± 0.029	6.24 ± 0.12	0.776 ± 0.011
35	1.738 ± 0.034	6.47 ± 0.15	0.801 ± 0.012
36	1.512 ± 0.022	6.05 ± 0.09	0.798 ± 0.008
39	1.846 ± 0.025	6.31 ± 0.09	0.786 ± 0.012
40	1.784 ± 0.030	6.32 ± 0.11	0.799 ± 0.011
41	1.802 ± 0.030	6.07 ± 0.11	0.779 ± 0.011
42	1.753 ± 0.030	5.99 ± 0.11	0.800 ± 0.011
43	1.718 ± 0.028	6.37 ± 0.10	0.786 ± 0.012

Continued on next page

res.	R_1/s^{-1}	R_2/s^{-1}	$\sigma\{^1\mathrm{H}\}$
44	1.741 ± 0.032	6.17 ± 0.13	0.800 ± 0.011
45	1.797 ± 0.034	6.68 ± 0.15	0.804 ± 0.012
46	1.846 ± 0.030	6.34 ± 0.11	0.773 ± 0.012
47	1.739 ± 0.024	5.74 ± 0.10	0.784 ± 0.011
48	1.749 ± 0.022	6.58 ± 0.09	0.788 ± 0.009
49	1.652 ± 0.015	5.73 ± 0.06	0.740 ± 0.009
50	1.789 ± 0.033	6.36 ± 0.12	0.780 ± 0.012
51	1.659 ± 0.034	6.35 ± 0.10	0.778 ± 0.014
52	1.572 ± 0.020	6.08 ± 0.08	0.783 ± 0.011
54	1.715 ± 0.028	6.83 ± 0.11	0.802 ± 0.011
55	1.783 ± 0.037	6.92 ± 0.13	0.777 ± 0.014
56	1.896 ± 0.032	6.41 ± 0.10	0.811 ± 0.011
57	1.857 ± 0.029	6.27 ± 0.09	0.793 ± 0.013
58	1.910 ± 0.032	7.10 ± 0.10	0.799 ± 0.012
59	1.774 ± 0.029	6.04 ± 0.10	0.796 ± 0.012
60	1.830 ± 0.033	6.43 ± 0.11	0.787 ± 0.012
61	1.806 ± 0.030	6.12 ± 0.11	0.796 ± 0.012
62	1.576 ± 0.021	5.71 ± 0.07	0.664 ± 0.011
63	1.678 ± 0.017	6.39 ± 0.07	0.778 ± 0.010
64	1.845 ± 0.031	6.18 ± 0.10	0.788 ± 0.011
65	1.836 ± 0.036	6.42 ± 0.10	0.813 ± 0.015
66	1.713 ± 0.019	6.16 ± 0.07	0.792 ± 0.010
67	1.799 ± 0.035	6.42 ± 0.14	0.804 ± 0.012
68	1.722 ± 0.039	6.09 ± 0.13	0.794 ± 0.012
69	1.758 ± 0.025	6.17 ± 0.10	0.805 ± 0.010
70	1.806 ± 0.037	7.54 ± 0.16	0.797 ± 0.012
71	1.730 ± 0.015	5.86 ± 0.06	0.771 ± 0.010
73	1.630 ± 0.013	3.94 ± 0.05	0.491 ± 0.007
74	1.467 ± 0.012	2.88 ± 0.03	0.286 ± 0.006
75	1.203 ± 0.010	1.66 ± 0.03	-0.052 ± 0.005
76	0.784 ± 0.006	1.20 ± 0.02	-0.544 ± 0.005

Table S3: Ubiquitin cross-correlated cross-relaxation (CCCR) rates measured at 800 MHz and 30°C. Residue numbers, longitudinal CCCR rates (η_z) measured by standard experiment, transverse CCCR rates (η_{xy}) measured by standard experiment, transverse CCCR rates ($\eta_{\text{ave}}^{xy}(\alpha-2)/\alpha$), and a linear combination of longitudinal and transverse CCCR rates (η_{ave}^{xyz}) measured by adiabatic experiment are shown in the first, second, third, fourth, and fifth column, respectively.

res	η_z/s^{-1}	η_{xy}/s^{-1}	$\eta_{\text{ave}}^{xy}\frac{\alpha-2}{\alpha}/\text{s}^{-1}$	$\eta_{\text{ave}}^{xyz}/\text{s}^{-1}$
2	1.310 ± 0.025	5.22 ± 0.05	5.23 ± 0.03	3.19 ± 0.02
3	1.397 ± 0.055	4.90 ± 0.09	4.83 ± 0.07	3.09 ± 0.05
4	1.357 ± 0.053	4.84 ± 0.09	4.79 ± 0.07	3.06 ± 0.06
5	1.309 ± 0.053	4.65 ± 0.08	4.73 ± 0.07	2.93 ± 0.05
6	1.466 ± 0.046	5.14 ± 0.07	5.13 ± 0.06	3.23 ± 0.05
7	1.346 ± 0.034	4.86 ± 0.06	4.84 ± 0.04	3.04 ± 0.03
8	1.278 ± 0.038	4.09 ± 0.05	4.15 ± 0.04	2.65 ± 0.03
9	1.190 ± 0.040	3.96 ± 0.05	3.84 ± 0.04	2.50 ± 0.03
10	1.291 ± 0.029	4.26 ± 0.04	4.16 ± 0.03	2.72 ± 0.02
11	1.294 ± 0.027	4.54 ± 0.04	4.56 ± 0.03	2.90 ± 0.03
12	1.242 ± 0.024	4.29 ± 0.04	4.30 ± 0.03	2.71 ± 0.02
13	1.388 ± 0.049	4.76 ± 0.07	4.83 ± 0.06	3.03 ± 0.04
14	1.318 ± 0.029	4.73 ± 0.05	4.78 ± 0.04	2.98 ± 0.02
15	1.411 ± 0.049	4.88 ± 0.08	4.83 ± 0.06	3.09 ± 0.07
16	1.250 ± 0.020	4.71 ± 0.03	4.70 ± 0.03	2.95 ± 0.03
17	1.348 ± 0.029	4.94 ± 0.05	4.98 ± 0.04	3.12 ± 0.03
18	1.179 ± 0.038	4.79 ± 0.07	4.76 ± 0.05	2.94 ± 0.03
20	1.272 ± 0.040	4.86 ± 0.07	4.77 ± 0.05	2.94 ± 0.04
21	1.514 ± 0.042	5.63 ± 0.07	5.67 ± 0.06	3.47 ± 0.05
22	1.331 ± 0.041	4.62 ± 0.06	4.57 ± 0.05	2.92 ± 0.05
23	1.467 ± 0.063	5.28 ± 0.10	5.26 ± 0.08	3.32 ± 0.10
25	1.411 ± 0.059	5.05 ± 0.10	5.07 ± 0.07	3.15 ± 0.04
26	1.454 ± 0.048	5.10 ± 0.07	5.07 ± 0.06	3.26 ± 0.04
27	1.445 ± 0.069	5.19 ± 0.10	5.17 ± 0.08	3.24 ± 0.05
28	1.472 ± 0.052	5.35 ± 0.08	5.45 ± 0.07	3.36 ± 0.04
29	1.398 ± 0.051	5.00 ± 0.08	4.97 ± 0.06	3.16 ± 0.04
30	1.422 ± 0.060	4.99 ± 0.08	5.05 ± 0.07	3.17 ± 0.06
32	1.384 ± 0.047	4.98 ± 0.07	4.94 ± 0.05	3.12 ± 0.04
33	1.329 ± 0.044	4.70 ± 0.07	4.67 ± 0.05	2.94 ± 0.05
34	1.250 ± 0.049	4.39 ± 0.07	4.36 ± 0.05	2.77 ± 0.05
35	1.259 ± 0.053	5.16 ± 0.09	5.03 ± 0.07	3.10 ± 0.05
36	1.246 ± 0.032	5.09 ± 0.06	5.13 ± 0.05	3.09 ± 0.03
39	1.350 ± 0.038	4.59 ± 0.06	4.53 ± 0.04	2.93 ± 0.04
40	1.327 ± 0.048	4.88 ± 0.07	4.92 ± 0.06	3.05 ± 0.04
41	1.407 ± 0.049	4.81 ± 0.07	4.83 ± 0.06	3.04 ± 0.03

Continued on next page

res	η_z/s^{-1}	$\eta_{xy}/\mathrm{s}^{-1}$	$\eta^{xy}_{\mathrm{ave}}\frac{\alpha-2}{\alpha}/\mathrm{s}^{-1}$	$\eta^{xyz}_{\mathrm{ave}}/\mathrm{s}^{-1}$
42	1.307 ± 0.043	4.62 ± 0.07	4.64 ± 0.06	2.94 ± 0.04
43	1.329 ± 0.041	4.78 ± 0.07	4.76 ± 0.05	2.98 ± 0.04
44	1.344 ± 0.047	4.70 ± 0.08	4.70 ± 0.06	2.97 ± 0.05
45	1.437 ± 0.052	5.31 ± 0.09	5.41 ± 0.07	3.33 ± 0.04
46	1.450 ± 0.053	5.07 ± 0.07	5.02 ± 0.05	3.15 ± 0.06
47	1.166 ± 0.031	4.02 ± 0.05	3.91 ± 0.04	2.50 ± 0.03
48	1.377 ± 0.034	5.37 ± 0.06	5.36 ± 0.04	3.34 ± 0.03
49	1.288 ± 0.021	4.44 ± 0.04	4.45 ± 0.03	2.83 ± 0.02
50	1.410 ± 0.051	5.04 ± 0.08	5.13 ± 0.06	3.16 ± 0.03
51	1.237 ± 0.054	4.80 ± 0.09	4.85 ± 0.07	2.98 ± 0.04
52	1.148 ± 0.028	4.66 ± 0.05	4.62 ± 0.04	2.82 ± 0.02
54	1.347 ± 0.042	5.32 ± 0.08	5.25 ± 0.05	3.25 ± 0.04
55	1.307 ± 0.054	4.57 ± 0.09	4.49 ± 0.07	2.83 ± 0.06
56	1.481 ± 0.047	5.05 ± 0.07	5.04 ± 0.06	3.22 ± 0.06
57	1.368 ± 0.050	4.69 ± 0.07	4.61 ± 0.05	2.96 ± 0.04
58	1.476 ± 0.056	5.14 ± 0.08	5.10 ± 0.06	3.25 ± 0.04
59	1.298 ± 0.048	4.57 ± 0.06	4.48 ± 0.06	2.90 ± 0.04
60	1.487 ± 0.058	5.14 ± 0.08	5.18 ± 0.06	3.27 ± 0.06
61	1.357 ± 0.044	4.74 ± 0.07	4.73 ± 0.06	3.01 ± 0.03
62	1.165 ± 0.028	4.17 ± 0.05	4.19 ± 0.04	2.60 ± 0.03
63	1.189 ± 0.023	4.67 ± 0.04	4.70 ± 0.03	2.88 ± 0.03
64	1.357 ± 0.046	4.66 ± 0.07	4.64 ± 0.05	2.92 ± 0.04
65	1.391 ± 0.069	5.04 ± 0.10	5.03 ± 0.08	3.17 ± 0.07
66	1.299 ± 0.031	4.63 ± 0.05	4.63 ± 0.04	2.94 ± 0.04
67	1.391 ± 0.052	4.84 ± 0.08	4.88 ± 0.06	3.05 ± 0.05
68	1.297 ± 0.057	4.62 ± 0.09	4.61 ± 0.07	2.88 ± 0.05
69	1.418 ± 0.037	5.02 ± 0.06	5.04 ± 0.05	3.15 ± 0.04
70	1.435 ± 0.053	5.16 ± 0.09	5.16 ± 0.07	3.23 ± 0.06
71	1.325 ± 0.022	4.50 ± 0.04	4.55 ± 0.03	2.87 ± 0.02
73	1.179 ± 0.018	2.77 ± 0.02	2.75 ± 0.02	1.93 ± 0.01
74	0.977 ± 0.015	2.00 ± 0.02	2.00 ± 0.02	1.48 ± 0.01
75	0.661 ± 0.011	1.09 ± 0.02	1.03 ± 0.01	0.85 ± 0.01
76	0.426 ± 0.006	0.68 ± 0.01	0.65 ± 0.01	0.54 ± 0.01

Table S4: Spectral density at zero frequency $J(0)$ measured at 800 MHz and 30°C in ubiquitin. Residue numbers, $J(0)$ values determined by adiabatic experiment $\eta^J_{\text{ave}}(\alpha-4)/(16C_{cd}\alpha)$ and single echo experiment $(-3\eta^J_{\text{ave}})/(16C_{cd})$ are shown in the first, second, and third column, respectively. $C_{cd}=(3\cos^2\varphi-1)\gamma_{\text{N}}^2\gamma_{\text{H}}B_0\Delta\sigma\mu\hbar r^{-3}_{\text{N-H}}/16$, γ_{H} and γ_{N} are the magnetogyric ratios of ^{1}H and ^{15}N, respectively, $r_{\text{N-H}}=1.02$ Åis the H-N internuclear distance, μ_0 is the permeability of vacuum, $\hbar$ is Planck's constant divided by 2π, $\Delta\sigma=-170$ ppm is the anisotropy of the ^{15}N chemical shielding tensor, $\varphi=20.6°$ is the angle between the H-N bond and the symmetry axis of the ^{15}N chemical shielding tensor, and B_0 is the external magnetic field.

res.	$\eta^J_{\text{ave}}\frac{\alpha-4}{16c\alpha}/\text{s}^{-1}$	$\eta^J_{\text{ave}}\frac{-3}{16c}/\text{s}^{-1}$
2	1.349 ± 0.006	1.325 ± 0.005
3	1.223 ± 0.013	1.222 ± 0.010
4	1.219 ± 0.012	1.192 ± 0.009
5	1.192 ± 0.012	1.166 ± 0.009
6	1.297 ± 0.011	1.301 ± 0.009
7	1.226 ± 0.008	1.214 ± 0.006
8	1.021 ± 0.008	1.017 ± 0.006
9	0.958 ± 0.008	1.044 ± 0.006
10	1.030 ± 0.006	1.072 ± 0.005
11	1.147 ± 0.006	1.153 ± 0.005
12	1.082 ± 0.006	1.074 ± 0.004
13	1.216 ± 0.011	1.198 ± 0.008
14	1.208 ± 0.007	1.187 ± 0.005
15	1.221 ± 0.011	1.217 ± 0.008
16	1.194 ± 0.005	1.198 ± 0.004
17	1.264 ± 0.007	1.216 ± 0.005
18	1.248 ± 0.010	1.234 ± 0.008
20	1.216 ± 0.009	1.224 ± 0.007
21	1.431 ± 0.010	1.457 ± 0.008
22	1.149 ± 0.009	1.179 ± 0.007
23	1.345 ± 0.015	1.365 ± 0.012
25	1.287 ± 0.014	1.271 ± 0.011
26	1.284 ± 0.010	1.266 ± 0.007
27	1.313 ± 0.015	1.305 ± 0.012
28	1.371 ± 0.012	1.389 ± 0.009
29	1.256 ± 0.012	1.263 ± 0.009
30	1.278 ± 0.013	1.292 ± 0.010
32	1.243 ± 0.010	1.258 ± 0.008
33	1.179 ± 0.010	1.167 ± 0.007
34	1.090 ± 0.010	1.093 ± 0.008
35	1.306 ± 0.012	1.344 ± 0.010
36	1.315 ± 0.009	1.308 ± 0.007

Continued on next page

res.	$\eta^J_{\text{ave}}\frac{\alpha-4}{16c\alpha}/\text{s}^{-1}$	$\eta^J_{\text{ave}}\frac{-3}{16c}/\text{s}^{-1}$
39	1.128 ± 0.008	1.137 ± 0.006
40	1.237 ± 0.010	1.235 ± 0.008
41	1.185 ± 0.011	1.195 ± 0.008
42	1.163 ± 0.010	1.192 ± 0.008
43	1.217 ± 0.010	1.208 ± 0.008
44	1.177 ± 0.011	1.218 ± 0.009
45	1.368 ± 0.012	1.337 ± 0.010
46	1.276 ± 0.011	1.291 ± 0.008
47	0.972 ± 0.007	0.994 ± 0.005
48	1.384 ± 0.008	1.365 ± 0.006
49	1.122 ± 0.005	1.111 ± 0.004
50	1.287 ± 0.011	1.260 ± 0.009
51	1.235 ± 0.013	1.250 ± 0.010
52	1.197 ± 0.007	1.232 ± 0.006
54	1.357 ± 0.010	1.380 ± 0.008
55	1.142 ± 0.013	1.152 ± 0.010
56	1.257 ± 0.011	1.261 ± 0.008
57	1.152 ± 0.010	1.143 ± 0.007
58	1.290 ± 0.012	1.284 ± 0.009
59	1.149 ± 0.011	1.156 ± 0.008
60	1.302 ± 0.012	1.285 ± 0.009
61	1.195 ± 0.010	1.232 ± 0.008
62	1.052 ± 0.007	1.045 ± 0.005
63	1.209 ± 0.006	1.190 ± 0.005
64	1.154 ± 0.010	1.178 ± 0.008
65	1.266 ± 0.014	1.292 ± 0.011
66	1.183 ± 0.007	1.171 ± 0.005
67	1.210 ± 0.012	1.259 ± 0.009
68	1.165 ± 0.013	1.177 ± 0.010
69	1.273 ± 0.009	1.245 ± 0.007
70	1.300 ± 0.013	1.298 ± 0.010
71	1.145 ± 0.005	1.162 ± 0.004
73	0.639 ± 0.004	0.621 ± 0.003
74	0.453 ± 0.003	0.461 ± 0.002
75	0.206 ± 0.003	0.207 ± 0.002
76	0.128 ± 0.002	0.137 ± 0.001

Table S5: Ubiquitin cross-correlated cross-relaxation (CCCR) rates measured at 500 MHz and 30°C. Residue numbers, longitudinal CCCR rates (η_z) measured by standard experiment, transverse CCCR rates (η_{xy}) measured by standard experiment, transverse CCCR rates ($\eta^{xy}_{\text{ave}}(\alpha-2)/\alpha$), and a linear combination of longitudinal and transverse CCCR rates (η^{xyz}_{ave}) measured by adiabatic experiment are shown in the first, second, third, fourth, and fifth column, respectively.

res	η_z/s^{-1}	η_{xy}/s^{-1}	$\eta^{xy}_{\text{ave}}\frac{\alpha-2}{\alpha}/\text{s}^{-1}$	$\eta^{xyz}_{\text{ave}}/\text{s}^{-1}$
2	1.573 ± 0.005	3.622 ± 0.021	3.706 ± 0.022	2.623 ± 0.011
3	1.602 ± 0.008	3.401 ± 0.033	3.456 ± 0.035	2.511 ± 0.018
4	1.572 ± 0.008	3.425 ± 0.032	3.429 ± 0.036	2.424 ± 0.017
5	1.513 ± 0.008	3.190 ± 0.031	3.356 ± 0.036	2.366 ± 0.018
6	1.720 ± 0.008	3.588 ± 0.030	3.621 ± 0.031	2.652 ± 0.015
7	1.533 ± 0.007	3.377 ± 0.025	3.479 ± 0.028	2.447 ± 0.013
8	1.417 ± 0.007	2.944 ± 0.025	2.912 ± 0.027	2.126 ± 0.013
9	1.332 ± 0.007	2.757 ± 0.025	2.761 ± 0.030	2.003 ± 0.014
10	1.436 ± 0.005	2.936 ± 0.018	2.969 ± 0.020	2.183 ± 0.011
11	1.467 ± 0.005	3.150 ± 0.020	3.174 ± 0.022	2.321 ± 0.011
12	1.424 ± 0.005	2.995 ± 0.019	3.057 ± 0.021	2.180 ± 0.010
13	1.598 ± 0.007	3.301 ± 0.030	3.429 ± 0.032	2.444 ± 0.016
14	1.543 ± 0.006	3.410 ± 0.023	3.351 ± 0.024	2.415 ± 0.012
15	1.642 ± 0.007	3.349 ± 0.030	3.503 ± 0.031	2.500 ± 0.015
16	1.453 ± 0.003	3.270 ± 0.017	3.311 ± 0.018	2.399 ± 0.009
17	1.572 ± 0.005	3.449 ± 0.022	3.460 ± 0.024	2.525 ± 0.011
18	1.433 ± 0.006	3.473 ± 0.029	3.377 ± 0.029	2.399 ± 0.014
20	1.535 ± 0.007	3.453 ± 0.028	3.382 ± 0.030	2.424 ± 0.015
21	1.783 ± 0.008	3.821 ± 0.031	4.017 ± 0.034	2.850 ± 0.017
22	1.526 ± 0.007	3.255 ± 0.028	3.222 ± 0.029	2.398 ± 0.015
23	1.733 ± 0.010	3.684 ± 0.042	3.736 ± 0.042	2.702 ± 0.021
25	1.678 ± 0.010	3.587 ± 0.040	3.514 ± 0.042	2.624 ± 0.021
26	1.670 ± 0.008	3.562 ± 0.030	3.621 ± 0.031	2.632 ± 0.016
27	1.688 ± 0.010	3.590 ± 0.038	3.647 ± 0.039	2.655 ± 0.020
28	1.735 ± 0.009	3.708 ± 0.034	3.905 ± 0.037	2.712 ± 0.018
29	1.657 ± 0.009	3.434 ± 0.033	3.430 ± 0.036	2.571 ± 0.017
30	1.662 ± 0.009	3.555 ± 0.033	3.538 ± 0.037	2.569 ± 0.018
32	1.640 ± 0.007	3.380 ± 0.028	3.477 ± 0.031	2.551 ± 0.016
33	1.543 ± 0.007	3.222 ± 0.026	3.247 ± 0.029	2.401 ± 0.014
34	1.452 ± 0.008	3.109 ± 0.028	3.085 ± 0.031	2.252 ± 0.015
35	1.540 ± 0.008	3.570 ± 0.030	3.570 ± 0.033	2.526 ± 0.017
36	1.521 ± 0.006	3.476 ± 0.030	3.526 ± 0.031	2.485 ± 0.015
39	1.548 ± 0.006	3.170 ± 0.023	3.233 ± 0.025	2.371 ± 0.012
40	1.566 ± 0.008	3.401 ± 0.029	3.404 ± 0.032	2.520 ± 0.016
41	1.615 ± 0.008	3.340 ± 0.030	3.352 ± 0.033	2.499 ± 0.015

Continued on next page

res	η_z/s^{-1}	$\eta_{xy}/\mathrm{s}^{-1}$	$\eta^{xy}_{\mathrm{ave}}\frac{\alpha-2}{\alpha}/\mathrm{s}^{-1}$	$\eta^{xyz}_{\mathrm{ave}}/\mathrm{s}^{-1}$
42	1.521 ± 0.007	3.245 ± 0.027	3.346 ± 0.031	2.385 ± 0.015
43	1.559 ± 0.007	3.288 ± 0.029	3.392 ± 0.031	2.463 ± 0.015
44	1.520 ± 0.008	3.315 ± 0.030	3.415 ± 0.033	2.387 ± 0.017
45	1.709 ± 0.009	3.613 ± 0.035	3.717 ± 0.037	2.691 ± 0.018
46	1.671 ± 0.011	3.512 ± 0.037	3.570 ± 0.041	2.608 ± 0.020
47	1.339 ± 0.006	2.827 ± 0.021	2.851 ± 0.025	2.043 ± 0.012
48	1.657 ± 0.006	3.727 ± 0.024	3.768 ± 0.026	2.726 ± 0.013
49	1.446 ± 0.004	3.108 ± 0.017	3.208 ± 0.019	2.280 ± 0.009
50	1.654 ± 0.007	3.561 ± 0.030	3.541 ± 0.031	2.580 ± 0.015
51	1.459 ± 0.010	3.340 ± 0.037	3.420 ± 0.041	2.413 ± 0.020
52	1.405 ± 0.005	3.183 ± 0.025	3.190 ± 0.026	2.310 ± 0.013
54	1.623 ± 0.007	3.675 ± 0.031	3.654 ± 0.032	2.672 ± 0.016
55	1.509 ± 0.008	3.207 ± 0.034	3.268 ± 0.037	2.339 ± 0.018
56	1.698 ± 0.008	3.547 ± 0.031	3.496 ± 0.034	2.629 ± 0.017
57	1.568 ± 0.009	3.238 ± 0.031	3.291 ± 0.033	2.406 ± 0.016
58	1.722 ± 0.011	3.570 ± 0.036	3.508 ± 0.041	2.676 ± 0.019
59	1.495 ± 0.009	3.165 ± 0.032	3.104 ± 0.037	2.362 ± 0.018
60	1.725 ± 0.010	3.588 ± 0.035	3.601 ± 0.038	2.656 ± 0.019
61	1.585 ± 0.007	3.258 ± 0.029	3.328 ± 0.032	2.475 ± 0.015
62	1.356 ± 0.006	2.835 ± 0.022	2.924 ± 0.025	2.123 ± 0.012
63	1.442 ± 0.004	3.222 ± 0.019	3.244 ± 0.019	2.336 ± 0.010
64	1.565 ± 0.007	3.234 ± 0.028	3.285 ± 0.031	2.389 ± 0.015
65	1.617 ± 0.011	3.507 ± 0.040	3.525 ± 0.046	2.580 ± 0.022
66	1.519 ± 0.006	3.201 ± 0.022	3.288 ± 0.025	2.394 ± 0.012
67	1.624 ± 0.007	3.275 ± 0.029	3.422 ± 0.031	2.502 ± 0.015
68	1.506 ± 0.008	3.240 ± 0.032	3.280 ± 0.035	2.358 ± 0.017
69	1.656 ± 0.006	3.574 ± 0.025	3.557 ± 0.027	2.606 ± 0.013
70	1.685 ± 0.008	3.508 ± 0.033	3.621 ± 0.036	2.663 ± 0.018
71	1.505 ± 0.004	3.094 ± 0.017	3.215 ± 0.019	2.359 ± 0.009
73	1.119 ± 0.001	1.860 ± 0.012	1.902 ± 0.013	1.542 ± 0.006
74	0.860 ± 0.002	1.352 ± 0.009	1.434 ± 0.011	1.116 ± 0.006
75	0.510 ± 0.002	0.759 ± 0.008	0.696 ± 0.010	0.605 ± 0.005
76	0.304 ± 0.001	0.447 ± 0.005	0.423 ± 0.007	0.381 ± 0.003

Table S6: Spectral density at zero frequency $J(0)$ measured at 500 MHz and 30°C in ubiquitin. Residue numbers, $J(0)$ values determined by adiabatic experiment $\eta^{J}_{\mathrm{ave}}(\alpha-4)/(16C_{cd}\alpha)$ and single echo experiment $(-3\eta^{J}_{\mathrm{ave}})/(16C_{cd})$ are shown in the first, second, and third column, respectively. $C_{cd} = (3\cos^2\varphi - 1)\gamma_{\mathrm{N}}^2\gamma_{\mathrm{H}}B_0\Delta\sigma\mu\hbar r_{\mathrm{N-H}}^{-3}/16$, γ_{H} and γ_{N} are the magnetogyric ratios of ^{1}H and ^{15}N, respectively, $r_{\mathrm{N-H}} = 1.02$ Åis the H-N internuclear distance, μ_0 is the permeability of vacuum, $\hbar$ is Planck's constant divided by 2π, $\Delta\sigma = -170$ ppm is the anisotropy of the ^{15}N chemical shielding tensor, $\varphi = 20.6°$ is the angle between the H-N bond and the symmetry axis of the ^{15}N chemical shielding tensor, and B_0 is the external magnetic field.

res.	$\eta^{J}_{\mathrm{ave}}\frac{\alpha-4}{16C_{cd}\alpha}/\mathrm{s}^{-1}$	$\frac{-3\eta^{J}_{\mathrm{ave}}}{16C_{cd}}/\mathrm{s}^{-1}$
2	1.360 ± 0.012	1.440 ± 0.007
3	1.237 ± 0.019	1.200 ± 0.011
4	1.248 ± 0.019	1.179 ± 0.012
5	1.201 ± 0.019	1.248 ± 0.012
6	1.282 ± 0.016	1.316 ± 0.011
7	1.260 ± 0.014	1.190 ± 0.009
8	1.024 ± 0.015	1.020 ± 0.009
9	0.988 ± 0.016	0.954 ± 0.009
10	1.036 ± 0.011	1.099 ± 0.006
11	1.154 ± 0.012	1.155 ± 0.007
12	1.104 ± 0.011	1.108 ± 0.007
13	1.215 ± 0.017	1.025 ± 0.011
14	1.207 ± 0.013	1.238 ± 0.008
15	1.253 ± 0.017	1.139 ± 0.010
16	1.214 ± 0.010	1.321 ± 0.006
17	1.259 ± 0.013	1.287 ± 0.008
18	1.253 ± 0.015	1.300 ± 0.010
20	1.225 ± 0.016	1.185 ± 0.010
21	1.467 ± 0.018	1.472 ± 0.011
22	1.161 ± 0.016	1.190 ± 0.010
23	1.336 ± 0.022	1.230 ± 0.014
25	1.272 ± 0.024	1.247 ± 0.014
26	1.295 ± 0.017	1.282 ± 0.010
27	1.301 ± 0.021	1.241 ± 0.013
28	1.393 ± 0.020	1.321 ± 0.012
29	1.212 ± 0.019	1.165 ± 0.011
30	1.271 ± 0.019	1.180 ± 0.011
32	1.226 ± 0.017	1.274 ± 0.010
33	1.151 ± 0.016	1.181 ± 0.009
34	1.118 ± 0.017	1.184 ± 0.011
35	1.313 ± 0.018	1.342 ± 0.011

Continued on next page

res.	$\eta^J_{\text{ave}} \frac{\alpha-4}{16C_{cd}\alpha}/\text{s}^{-1}$	$\frac{-3\eta^J_{\text{ave}}}{16C_{cd}}/\text{s}^{-1}$
36	1.309 ± 0.016	1.193 ± 0.010
39	1.157 ± 0.013	1.164 ± 0.008
40	1.220 ± 0.017	1.194 ± 0.010
41	1.195 ± 0.017	1.179 ± 0.010
42	1.190 ± 0.016	1.155 ± 0.010
43	1.191 ± 0.016	1.091 ± 0.009
44	1.215 ± 0.017	1.106 ± 0.010
45	1.370 ± 0.019	1.307 ± 0.012
46	1.256 ± 0.022	1.340 ± 0.014
47	1.010 ± 0.013	0.869 ± 0.007
48	1.373 ± 0.014	1.349 ± 0.008
49	1.151 ± 0.010	1.153 ± 0.006
50	1.278 ± 0.017	1.320 ± 0.011
51	1.242 ± 0.022	1.311 ± 0.013
52	1.176 ± 0.014	1.135 ± 0.009
54	1.346 ± 0.017	1.222 ± 0.011
55	1.172 ± 0.020	1.107 ± 0.012
56	1.248 ± 0.018	1.309 ± 0.011
57	1.165 ± 0.018	1.151 ± 0.011
58	1.292 ± 0.023	1.211 ± 0.013
59	1.137 ± 0.020	1.116 ± 0.012
60	1.315 ± 0.020	1.247 ± 0.012
61	1.227 ± 0.017	1.226 ± 0.010
62	1.043 ± 0.013	1.051 ± 0.008
63	1.200 ± 0.010	1.097 ± 0.006
64	1.185 ± 0.016	1.181 ± 0.010
65	1.304 ± 0.024	1.285 ± 0.015
66	1.196 ± 0.013	1.207 ± 0.008
67	1.243 ± 0.017	1.085 ± 0.010
68	1.179 ± 0.019	1.090 ± 0.011
69	1.279 ± 0.014	1.206 ± 0.008
70	1.331 ± 0.019	1.405 ± 0.013
71	1.160 ± 0.010	1.001 ± 0.006
73	0.627 ± 0.006	0.492 ± 0.004
74	0.456 ± 0.006	0.445 ± 0.003
75	0.208 ± 0.005	0.206 ± 0.003
76	0.148 ± 0.003	0.174 ± 0.002

Table S7: Ubiquitin R_1, R_2, and steady-state nuclear Overhauser effect (NOE) ratios measured at 500 MHz and 5°C. Residue numbers, longitudinal relaxation rates (R_1), transverse relaxation rates (R_2), and NOE ratios ($\sigma\{^1\mathrm{H}\}$) are shown in the first, second, third, and fourth column, respectively.

res.	R_1/s^{-1}	R_2/s^{-1}	$\sigma\{^1\mathrm{H}\}$
2	1.745 ± 0.020	9.50 ± 0.08	0.737 ± 0.010
3	1.893 ± 0.032	9.23 ± 0.11	0.782 ± 0.011
4	1.908 ± 0.033	9.30 ± 0.11	0.791 ± 0.011
5	1.819 ± 0.034	8.95 ± 0.12	0.781 ± 0.012
6	1.885 ± 0.031	9.39 ± 0.12	0.784 ± 0.010
7	1.848 ± 0.024	9.18 ± 0.08	0.746 ± 0.011
8	1.886 ± 0.024	8.44 ± 0.08	0.659 ± 0.012
9	1.766 ± 0.025	8.27 ± 0.09	0.582 ± 0.012
10	1.818 ± 0.019	7.87 ± 0.06	0.607 ± 0.009
11	1.705 ± 0.020	8.08 ± 0.06	0.564 ± 0.009
12	1.758 ± 0.016	8.25 ± 0.05	0.670 ± 0.008
13	1.852 ± 0.035	9.82 ± 0.15	0.748 ± 0.013
14	1.802 ± 0.021	9.68 ± 0.08	0.765 ± 0.011
15	1.904 ± 0.032	9.14 ± 0.11	0.787 ± 0.010
16	1.699 ± 0.016	8.78 ± 0.06	0.725 ± 0.008
17	1.843 ± 0.020	9.31 ± 0.06	0.759 ± 0.009
18	1.709 ± 0.028	9.47 ± 0.10	0.769 ± 0.013
20	1.792 ± 0.027	9.18 ± 0.11	0.757 ± 0.013
22	1.884 ± 0.026	9.08 ± 0.09	0.777 ± 0.014
23	1.936 ± 0.048	12.03 ± 0.20	0.788 ± 0.016
25	1.889 ± 0.042	16.08 ± 0.27	0.805 ± 0.018
26	1.915 ± 0.031	9.35 ± 0.10	0.789 ± 0.012
27	1.920 ± 0.043	9.87 ± 0.15	0.777 ± 0.014
29	1.893 ± 0.040	9.63 ± 0.15	0.818 ± 0.015
30	1.919 ± 0.039	9.59 ± 0.15	0.781 ± 0.013
31	1.914 ± 0.041	9.80 ± 0.16	0.793 ± 0.014
32	1.864 ± 0.031	9.60 ± 0.12	0.790 ± 0.012
33	1.837 ± 0.032	9.65 ± 0.14	0.776 ± 0.013
34	1.829 ± 0.035	9.20 ± 0.14	0.767 ± 0.014
35	1.780 ± 0.036	9.87 ± 0.17	0.808 ± 0.015
36	1.539 ± 0.060	8.86 ± 0.14	0.779 ± 0.014
39	1.878 ± 0.023	9.27 ± 0.09	0.786 ± 0.011
40	1.862 ± 0.033	9.27 ± 0.13	0.776 ± 0.015
41	1.872 ± 0.033	9.09 ± 0.12	0.761 ± 0.013
43	1.816 ± 0.035	9.67 ± 0.12	0.771 ± 0.015
44	1.858 ± 0.029	9.15 ± 0.10	0.786 ± 0.009
45	1.845 ± 0.035	10.00 ± 0.15	0.783 ± 0.013

Continued on next page

res.	R_1/s^{-1}	R_2/s^{-1}	$\sigma\{^1\mathrm{H}\}$
46	1.831 ± 0.028	9.18 ± 0.12	0.736 ± 0.012
47	1.781 ± 0.022	8.68 ± 0.10	0.725 ± 0.011
48	1.763 ± 0.022	9.66 ± 0.10	0.740 ± 0.010
49	1.732 ± 0.015	8.59 ± 0.06	0.680 ± 0.009
50	1.870 ± 0.030	9.27 ± 0.11	0.753 ± 0.012
52	1.599 ± 0.020	9.24 ± 0.08	0.736 ± 0.011
54	1.746 ± 0.026	9.65 ± 0.10	0.770 ± 0.011
55	1.830 ± 0.033	10.08 ± 0.13	0.766 ± 0.015
56	1.946 ± 0.031	9.15 ± 0.09	0.797 ± 0.012
57	1.908 ± 0.030	9.09 ± 0.10	0.776 ± 0.014
58	1.934 ± 0.037	9.70 ± 0.12	0.773 ± 0.016
59	1.851 ± 0.033	8.87 ± 0.11	0.789 ± 0.014
60	1.899 ± 0.034	9.24 ± 0.12	0.763 ± 0.013
61	1.884 ± 0.036	9.13 ± 0.12	0.783 ± 0.013
62	1.691 ± 0.023	7.91 ± 0.07	0.562 ± 0.011
63	1.717 ± 0.018	9.38 ± 0.07	0.772 ± 0.010
64	1.927 ± 0.029	9.11 ± 0.10	0.774 ± 0.012
65	1.864 ± 0.039	9.25 ± 0.13	0.790 ± 0.018
66	1.798 ± 0.021	8.79 ± 0.07	0.780 ± 0.011
67	1.854 ± 0.030	9.29 ± 0.12	0.777 ± 0.011
68	1.816 ± 0.036	8.99 ± 0.13	0.794 ± 0.012
69	1.853 ± 0.027	9.30 ± 0.10	0.780 ± 0.010
70	1.886 ± 0.041	13.26 ± 0.22	0.774 ± 0.016
71	1.847 ± 0.017	8.90 ± 0.06	0.722 ± 0.009
72	1.893 ± 0.021	8.13 ± 0.07	0.696 ± 0.010
73	1.934 ± 0.014	5.99 ± 0.04	0.478 ± 0.007
74	1.772 ± 0.011	4.65 ± 0.03	0.210 ± 0.005
75	1.587 ± 0.008	2.95 ± 0.02	-0.128 ± 0.004
76	1.177 ± 0.005	2.09 ± 0.01	-0.569 ± 0.004

Table S8: Ubiquitin cross-correlated cross-relaxation (CCCR) rates measured at 500 MHz and 5°C. Residue numbers, longitudinal CCCR rates (η_z) measured by standard experiment, transverse CCCR rates (η_{xy}) measured by standard experiment, transverse CCCR rates ($\eta^{xy}_{\text{ave}}(\alpha-2)/\alpha$), and a linear combination of longitudinal and transverse CCCR rates (η^{xyz}_{ave}) measured by adiabatic experiment are shown in the first, second, third, fourth, and fifth column, respectively.

res.	η_z/s^{-1}	η_{xy}/s^{-1}	$\eta^{xy}_{\text{ave}}\frac{\alpha-2}{\alpha}/\text{s}^{-1}$	$\eta^{xyz}_{\text{ave}}/\text{s}^{-1}$
2	1.117 ± 0.007	6.58 ± 0.06	6.63 ± 0.05	3.84 ± 0.03
3	1.189 ± 0.012	6.08 ± 0.10	5.98 ± 0.07	3.58 ± 0.05
4	1.159 ± 0.012	5.92 ± 0.10	6.02 ± 0.08	3.54 ± 0.05
5	1.125 ± 0.012	5.76 ± 0.10	5.82 ± 0.08	3.39 ± 0.05
6	1.277 ± 0.011	6.62 ± 0.09	6.32 ± 0.07	3.83 ± 0.04
7	1.148 ± 0.009	6.09 ± 0.07	6.07 ± 0.06	3.55 ± 0.04
8	1.132 ± 0.010	5.31 ± 0.07	5.24 ± 0.05	3.17 ± 0.03
9	1.082 ± 0.011	5.09 ± 0.07	4.84 ± 0.06	2.97 ± 0.04
10	1.151 ± 0.008	5.36 ± 0.05	5.39 ± 0.04	3.23 ± 0.03
11	1.149 ± 0.008	5.86 ± 0.05	5.96 ± 0.05	3.48 ± 0.03
12	1.108 ± 0.006	5.42 ± 0.04	5.41 ± 0.03	3.23 ± 0.02
13	1.192 ± 0.014	5.92 ± 0.11	6.08 ± 0.08	3.57 ± 0.05
14	1.126 ± 0.008	5.89 ± 0.07	5.95 ± 0.05	3.48 ± 0.03
15	1.227 ± 0.011	6.13 ± 0.09	6.10 ± 0.07	3.62 ± 0.04
16	1.063 ± 0.005	5.97 ± 0.04	5.91 ± 0.03	3.48 ± 0.02
17	1.152 ± 0.008	6.19 ± 0.06	6.27 ± 0.05	3.65 ± 0.03
18	0.991 ± 0.009	6.09 ± 0.09	6.07 ± 0.07	3.49 ± 0.04
20	1.121 ± 0.011	6.04 ± 0.08	5.99 ± 0.07	3.51 ± 0.04
22	1.131 ± 0.010	5.77 ± 0.07	5.77 ± 0.06	3.43 ± 0.04
23	1.286 ± 0.019	6.53 ± 0.18	6.56 ± 0.12	3.80 ± 0.08
25	1.227 ± 0.022	6.27 ± 0.23	6.14 ± 0.14	3.72 ± 0.09
26	1.257 ± 0.015	6.39 ± 0.10	6.38 ± 0.08	3.80 ± 0.05
27	1.245 ± 0.019	6.45 ± 0.14	6.29 ± 0.11	3.75 ± 0.07
29	1.218 ± 0.017	6.17 ± 0.13	6.05 ± 0.09	3.65 ± 0.06
30	1.233 ± 0.017	6.39 ± 0.13	6.18 ± 0.09	3.73 ± 0.06
31	1.244 ± 0.019	6.75 ± 0.13	6.66 ± 0.11	3.90 ± 0.07
32	1.201 ± 0.013	6.17 ± 0.10	6.08 ± 0.08	3.66 ± 0.05
33	1.134 ± 0.014	5.80 ± 0.10	5.77 ± 0.08	3.49 ± 0.05
34	1.086 ± 0.016	5.51 ± 0.12	5.36 ± 0.09	3.26 ± 0.06
35	1.082 ± 0.016	6.51 ± 0.13	6.34 ± 0.10	3.65 ± 0.06
36	1.079 ± 0.022	6.54 ± 0.22	6.38 ± 0.16	3.71 ± 0.10
39	1.174 ± 0.009	5.71 ± 0.07	5.69 ± 0.05	3.41 ± 0.03
40	1.145 ± 0.014	6.07 ± 0.11	6.07 ± 0.09	3.60 ± 0.05
41	1.227 ± 0.013	6.09 ± 0.10	6.03 ± 0.08	3.59 ± 0.05
43	1.148 ± 0.013	5.93 ± 0.10	5.95 ± 0.08	3.57 ± 0.05

Continued on next page

res.	η_z/s^{-1}	$\eta_{xy}/\mathrm{s}^{-1}$	$\eta_{\mathrm{ave}}^{xy}\frac{\alpha-2}{\alpha}/\mathrm{s}^{-1}$	$\eta_{\mathrm{ave}}^{xyz}/\mathrm{s}^{-1}$
44	1.136 ± 0.010	6.01 ± 0.09	5.91 ± 0.07	3.46 ± 0.04
45	1.236 ± 0.014	6.61 ± 0.11	6.65 ± 0.09	3.88 ± 0.05
46	1.258 ± 0.010	6.47 ± 0.08	6.33 ± 0.06	3.78 ± 0.04
47	1.014 ± 0.007	5.08 ± 0.05	4.89 ± 0.05	2.95 ± 0.03
48	1.174 ± 0.009	6.77 ± 0.08	6.79 ± 0.06	3.94 ± 0.04
49	1.118 ± 0.005	5.61 ± 0.04	5.58 ± 0.03	3.38 ± 0.02
50	1.233 ± 0.011	6.27 ± 0.09	6.40 ± 0.07	3.72 ± 0.05
52	0.984 ± 0.007	5.85 ± 0.07	5.81 ± 0.05	3.34 ± 0.03
54	1.159 ± 0.010	6.63 ± 0.09	6.60 ± 0.07	3.89 ± 0.04
55	1.113 ± 0.012	5.68 ± 0.10	5.64 ± 0.08	3.37 ± 0.05
56	1.280 ± 0.012	6.27 ± 0.09	6.25 ± 0.07	3.73 ± 0.04
57	1.180 ± 0.015	5.85 ± 0.10	5.73 ± 0.08	3.42 ± 0.05
58	1.296 ± 0.019	6.37 ± 0.12	6.39 ± 0.10	3.87 ± 0.06
59	1.109 ± 0.016	5.67 ± 0.10	5.54 ± 0.08	3.37 ± 0.05
60	1.303 ± 0.016	6.42 ± 0.11	6.35 ± 0.09	3.80 ± 0.06
61	1.167 ± 0.013	5.90 ± 0.10	6.05 ± 0.08	3.52 ± 0.05
62	1.032 ± 0.008	5.22 ± 0.06	5.16 ± 0.05	3.09 ± 0.03
63	1.020 ± 0.006	5.91 ± 0.06	5.83 ± 0.04	3.37 ± 0.03
64	1.168 ± 0.011	5.89 ± 0.09	5.83 ± 0.07	3.41 ± 0.04
65	1.180 ± 0.020	6.31 ± 0.14	6.30 ± 0.11	3.70 ± 0.07
66	1.119 ± 0.009	5.82 ± 0.06	5.85 ± 0.05	3.42 ± 0.03
67	1.194 ± 0.011	6.04 ± 0.09	6.16 ± 0.07	3.56 ± 0.04
68	1.108 ± 0.013	5.71 ± 0.10	5.77 ± 0.08	3.40 ± 0.05
69	1.214 ± 0.006	6.20 ± 0.08	6.23 ± 0.06	3.70 ± 0.04
70	1.225 ± 0.015	6.45 ± 0.17	6.61 ± 0.11	3.76 ± 0.07
71	1.179 ± 0.006	5.69 ± 0.05	5.75 ± 0.04	3.41 ± 0.02
72	1.192 ± 0.006	5.45 ± 0.05	5.41 ± 0.04	3.28 ± 0.03
73	1.189 ± 0.005	3.84 ± 0.02	3.89 ± 0.02	2.49 ± 0.01
74	1.013 ± 0.003	2.95 ± 0.02	2.93 ± 0.02	1.95 ± 0.01
75	0.806 ± 0.002	1.68 ± 0.01	1.67 ± 0.01	1.24 ± 0.01
76	0.612 ± 0.001	1.14 ± 0.01	1.20 ± 0.01	0.88 ± 0.01

Table S9: Spectral density at zero frequency $J(0)$ measured at 500 MHz and 5°C in ubiquitin. Residue numbers, $J(0)$ values determined by adiabatic experiment $\eta^{J}_{\text{ave}}(\alpha - 4)/(16C_{cd}\alpha)$ and single echo experiment $(-3\eta^{J}_{\text{ave}})/(16C_{cd})$ are shown in the first, second, and third column, respectively. $C_{cd} = (3\cos^2\varphi - 1)\gamma_{\text{N}}^2\gamma_{\text{H}}B_0\Delta\sigma\mu\hbar r_{\text{N-H}}^{-3}/16$, γ_{H} and γ_{N} are the magnetogyric ratios of ^{1}H and ^{15}N, respectively, $r_{\text{N-H}} = 1.02$ Åis the H-N internuclear distance, μ_0 is the permeability of vacuum, $\hbar$ is Planck's constant divided by 2π, $\Delta\sigma = -170$ ppm is the anisotropy of the ^{15}N chemical shielding tensor, $\varphi = 20.6°$ is the angle between the H-N bond and the symmetry axis of the ^{15}N chemical shielding tensor, and B_0 is the external magnetic field.

res.	$\eta^{J}_{\text{ave}}\frac{\alpha-4}{16C_{cd}\alpha}/\text{s}^{-1}$	$\frac{-3\eta^{J}_{\text{ave}}}{16C_{cd}}/\text{s}^{-1}$
2	2.862 ± 0.025	2.865 ± 0.021
3	2.637 ± 0.039	2.486 ± 0.029
4	2.522 ± 0.038	2.505 ± 0.032
5	2.499 ± 0.039	2.574 ± 0.033
6	2.741 ± 0.036	2.927 ± 0.031
7	2.626 ± 0.030	2.684 ± 0.025
8	2.238 ± 0.028	2.136 ± 0.021
9	2.018 ± 0.031	2.210 ± 0.022
10	2.246 ± 0.023	2.290 ± 0.017
11	2.511 ± 0.024	2.489 ± 0.018
12	2.299 ± 0.017	2.315 ± 0.014
13	2.597 ± 0.043	2.377 ± 0.036
14	2.539 ± 0.026	2.376 ± 0.021
15	2.565 ± 0.035	2.684 ± 0.028
16	2.544 ± 0.018	2.599 ± 0.014
17	2.690 ± 0.025	2.745 ± 0.020
18	2.608 ± 0.033	2.500 ± 0.029
20	2.510 ± 0.036	2.635 ± 0.029
22	2.411 ± 0.031	2.480 ± 0.025
23	2.804 ± 0.065	2.749 ± 0.059
25	2.695 ± 0.074	2.791 ± 0.080
26	2.672 ± 0.043	2.640 ± 0.032
27	2.699 ± 0.055	2.712 ± 0.047
29	2.498 ± 0.050	2.545 ± 0.043
30	2.647 ± 0.052	2.780 ± 0.042
31	2.769 ± 0.057	2.892 ± 0.045
32	2.626 ± 0.040	2.728 ± 0.035
33	2.452 ± 0.043	2.503 ± 0.035
34	2.282 ± 0.048	2.312 ± 0.037
35	2.749 ± 0.054	2.769 ± 0.044
36	2.781 ± 0.083	3.013 ± 0.078

Continued on next page

res.	$\eta^J_{\mathrm{ave}} \frac{\alpha-4}{16C_{cd}\alpha} / \mathrm{s}^{-1}$	$\frac{-3\eta^J_{\mathrm{ave}}}{16C_{cd}} / \mathrm{s}^{-1}$
39	2.416 ± 0.028	2.491 ± 0.023
40	2.563 ± 0.044	2.583 ± 0.035
41	2.550 ± 0.040	2.600 ± 0.034
43	2.534 ± 0.042	2.580 ± 0.035
44	2.504 ± 0.033	2.606 ± 0.027
45	2.841 ± 0.044	2.757 ± 0.037
46	2.693 ± 0.030	2.609 ± 0.025
47	2.089 ± 0.024	1.942 ± 0.018
48	2.936 ± 0.029	2.908 ± 0.024
49	2.357 ± 0.017	2.370 ± 0.014
50	2.745 ± 0.037	2.653 ± 0.029
52	2.499 ± 0.025	2.598 ± 0.022
54	2.880 ± 0.035	2.983 ± 0.031
55	2.453 ± 0.041	2.420 ± 0.033
56	2.640 ± 0.037	2.602 ± 0.028
57	2.412 ± 0.040	2.368 ± 0.032
58	2.625 ± 0.052	2.830 ± 0.042
59	2.374 ± 0.045	2.407 ± 0.033
60	2.699 ± 0.046	2.809 ± 0.035
61	2.557 ± 0.042	2.540 ± 0.035
62	2.203 ± 0.025	2.179 ± 0.020
63	2.501 ± 0.020	2.623 ± 0.018
64	2.442 ± 0.035	2.405 ± 0.028
65	2.718 ± 0.057	2.705 ± 0.047
66	2.467 ± 0.028	2.359 ± 0.020
67	2.614 ± 0.036	2.482 ± 0.029
68	2.482 ± 0.043	2.436 ± 0.034
69	2.673 ± 0.031	2.605 ± 0.024
70	2.824 ± 0.057	2.628 ± 0.054
71	2.449 ± 0.019	2.571 ± 0.015
72	2.285 ± 0.021	2.170 ± 0.016
73	1.533 ± 0.011	1.490 ± 0.008
74	1.139 ± 0.008	1.215 ± 0.005
75	0.594 ± 0.006	0.555 ± 0.003
76	0.421 ± 0.004	0.394 ± 0.002

Table S10: KIX transverse cross-correlated cross-relaxation (CCCR) rates measured at 500 MHz and 20°C. The KIX residue number, transverse CCCR rates (η_{xy}) measured by standard experiment, and transverse CCCR rates ($\eta_{\text{ave}}^{xy}(\alpha-2)/\alpha$) measured with the adiabatic scheme are shown in the first, second, and third column, respectively.

res.	η_{xy}/s^{-1}	$\eta_{\text{ave}}^{xy}\frac{\alpha-2}{\alpha}/\text{s}^{-1}$
582	8.83 ± 0.42	8.14 ± 0.37
585	1.15 ± 0.02	1.08 ± 0.03
586	1.25 ± 0.02	1.22 ± 0.02
587	2.81 ± 0.02	2.84 ± 0.01
588	3.59 ± 0.04	3.62 ± 0.04
589	4.82 ± 0.12	4.43 ± 0.09
590	6.26 ± 0.24	5.81 ± 0.28
593	7.87 ± 0.36	7.47 ± 0.28
594	5.83 ± 0.39	5.96 ± 0.33
595	8.13 ± 0.32	7.52 ± 0.25
596	6.13 ± 0.23	5.93 ± 0.17
597	8.50 ± 0.35	8.10 ± 0.28
598	9.57 ± 0.35	8.96 ± 0.30
599	9.69 ± 0.45	9.10 ± 0.46
600	9.19 ± 0.56	9.02 ± 0.51
601	9.64 ± 0.58	8.66 ± 0.52
602	10.00 ± 0.52	9.86 ± 0.54
603	9.99 ± 0.39	9.35 ± 0.37
604	8.90 ± 0.44	8.81 ± 0.41
605	9.20 ± 0.69	9.10 ± 0.59
606	9.59 ± 0.45	9.21 ± 0.36
607	9.41 ± 0.83	8.85 ± 0.66
608	9.19 ± 0.49	8.91 ± 0.39
609	9.37 ± 0.58	8.91 ± 0.45
610	9.55 ± 0.65	9.09 ± 0.55
611	8.27 ± 0.67	8.23 ± 0.54
612	8.48 ± 0.43	7.79 ± 0.30
614	5.17 ± 0.17	4.88 ± 0.11
618	5.80 ± 0.11	5.68 ± 0.08
619	6.11 ± 0.07	6.22 ± 0.07
620	5.06 ± 0.15	5.07 ± 0.11
621	5.22 ± 0.15	5.06 ± 0.11
622	6.32 ± 0.09	6.51 ± 0.10
623	5.92 ± 0.58	6.17 ± 0.41
624	6.89 ± 0.26	7.44 ± 0.26
626	8.58 ± 0.35	8.37 ± 0.31
628	8.51 ± 0.41	8.55 ± 0.38

Continued on next page

res.	η_{xy}/s^{-1}	$\eta^{xy}_{\text{ave}}\frac{\alpha-2}{\alpha}/\text{s}^{-1}$
629	8.50 ± 0.25	8.96 ± 0.32
630	9.39 ± 0.45	9.64 ± 0.42
632	9.48 ± 0.60	8.94 ± 0.59
633	8.76 ± 0.54	8.94 ± 0.53
634	9.62 ± 0.40	9.85 ± 0.46
635	9.00 ± 0.36	8.70 ± 0.31
636	9.60 ± 0.48	9.41 ± 0.44
637	9.60 ± 0.44	8.98 ± 0.36
638	10.13 ± 0.49	9.70 ± 0.40
640	9.61 ± 0.64	9.28 ± 0.67
641	9.61 ± 0.53	9.34 ± 0.47
642	8.59 ± 0.51	8.48 ± 0.53
643	8.64 ± 0.34	8.51 ± 0.32
644	7.72 ± 0.25	7.50 ± 0.26
645	8.79 ± 0.53	7.66 ± 0.40
646	9.93 ± 0.47	10.05 ± 0.54
647	9.73 ± 0.38	9.86 ± 0.37
650	10.00 ± 0.86	9.83 ± 0.83
651	9.90 ± 0.41	10.48 ± 0.51
652	9.97 ± 0.45	10.33 ± 0.54
656	9.79 ± 0.34	9.93 ± 0.36
657	9.65 ± 0.36	9.71 ± 0.42
658	7.53 ± 0.25	7.40 ± 0.24
659	9.67 ± 0.36	10.09 ± 0.43
660	9.07 ± 0.46	9.47 ± 0.50
662	9.33 ± 0.29	10.02 ± 0.31
665	8.22 ± 0.23	8.96 ± 0.24
666	8.51 ± 0.17	8.75 ± 0.20
670	4.27 ± 0.07	4.23 ± 0.06
671	3.09 ± 0.03	3.09 ± 0.03
672	1.73 ± 0.01	1.72 ± 0.01

Bruker pulse program for the measurement of transverse CCCR rates (η_{xy}) under an adiabatically swept pulse (scheme 1 in Fig. 1):

```
#include <Avance.incl>
#include <Grad.incl>


"d13=p10*(0.5*(1-cnst1))-9u-p12-100u"
"d14=p10-p12-p13-12u"
"d16=300u"
"d15=5u"
"d2 = 1/(4*cnst4)"
"d4 = d2-p16-d16-d15"
"d5 = d2-p17-d16-d15-p11"
"d30 = 30m"
"d0 = 3u"
"d10 = p1*2+d0*2"
"d22 = d2+p1+d0"
"d23 = d2-p1-d0-3u"
"in0=inf1/2"
"l5=1"


1 ze
2 d30 do:f2
3 d1 pl1:f1 pl0:f2
3u
(p9:sp9):f2
10u
10u pl2:f2
(p2 ph0):f2
d15 UNBLKGRAD
p16:gp0
1m
(p1 ph0):f1
d15
p16:gp1
d16
d4
(center (p1*2 ph2):f1 (p2*2 ph0):f2)
d4
d15
p16:gp1
```

```
d16
(p1 ph1):f1
d15
p20:gp2
2m

if "l5 if "l5 1u
(p2 ph10):f2
d2
(center (p1*2 ph0):f1 (p2*2 ph0):f2)
d2
(p2 ph1):f2
d15
p16:gp5
d16
(p1 ph2):f1
1u

goto 30

20 d15
p16:gp5
d16
(p1 ph0):f1
3u
(p1 ph10):f1
1u

;******* relaxation *******

30 3u
d13 pl0:f1 pl0:f2
100u fq=cnst12:f1
(p12:sp12 ph21:r):f1
3u
3u pl1:f1
(p1 ph0):f1
3u
(p1*2 ph1):f1
3u
(p1 ph0):f1

(center (p10:sp10):f2 (3u 3u pl0 p13:sp13 ph22:r d14 p12:sp12 ph21:r
3u 3u pl1):f1)
```

```
(p1 ph0):f1
3u
(p1*2 ph1):f1
3u
(p1 ph0):f1
3u
3u pl0:f1
(p13:sp13 ph22:r):f1
3u
d13 pl1:f1 pl2:f2
100u fq=0:f1



;******** end relaxation **********

if "l5 if "l5
1u
(p1 ph0):f1
d15
p16:gp7
d16
(p2 ph5):f2
d22
(p2*2 ph24):f2
d0
(p1*2 ph1):f1
d23
d0
(p2 ph1):f2
3u
goto 50

40 1u
(p1 ph0):f1
3u
(p1 ph14):f1
d15
p16:gp7
d16
(p2 ph5):f2
d0
(p1*2 ph0):f1
```

```
d0
(p2*2 ph0):f2
d10
(p2 ph0):f2

50 d15
p16:gp3
1m
(p1 ph0):f1
d15
p17:gp4
d16
d5
3u pl0:f1
3u
(p11:sp1 ph23:r):f1
3u
3u pl1:f1
3u
(center (p1*2 ph1):f1 (p2*2 ph0):f2)
3u
3u pl0:f1
3u
(p11:sp1 ph23:r):f1
6u
d15
p17:gp4
d16 BLKGRAD
d5 pl16:f2
go=2 ph31 cpds2:f2
d30 do:f2 mc #0 to 2
F1I(iu5, 4)
F1PH(ru5 & ip5, id0)
d30 do:f2
exit

ph0=0
ph1=1
ph21=1
ph22=1
ph2=2
ph3=3
ph23=3
ph5=0*1 2*1
```

```
ph10 = 0*4 2*4
ph11 = 0*8
ph12 = 1*8
ph14 = 0*2 2*2
ph15 = 2*2 0*2
ph20 = 2*4 0*4
ph24 = 0*2 1*2


ph31=0 2 2 0 2 0 0 2



;pl1 :  f1 channel - power level for pulse (default)
;p1 :  f1 channel - high power pulse
;p11 :  sp11 watergate
;p12 :  sp12 flip back z->x
;p13 :  sp13 flip back x->z
;d1 :  relaxation delay; 1-5 * T1
;cnst1 :  x/z proportion coefitient of used Chirp pulse during relaxation
;cnst4 :  JNH coupling
;cnst12 :  offset [Hz] to the center of amide region
```

Acquisition parameters for the measurement of transverse CCCR rates (η_{xy}) using an adiabatically swept pulse (scheme 1 in Fig. 1) at 500 MHz and 30°C:

```
##TITLE= Parameter file, TOPSPIN Version 2.1
##JCAMPDX= 5.0
##DATATYPE= Parameter Values
##NPOINTS= 12 $$ modification sequence number
##ORIGIN= Bruker BioSpin GmbH
##OWNER= username
$$ 2014-12-07 01:47:40.265 +0100 username@nmrspectrometer
$$ /opt/data/username/nmrspectrometer/Ubiq/32/acqus
$$ process /opt/topspin/prog/mod/shimcntl
##$ACQT0= 1000000
##$AMP= (0..31)
100 100 100 100 100 100 100 100 100 100 100 100 100 100 100 100 100
100
100 100 100 100 100 100 100 100 100 100 100 100 100 100
##$ANAVPT= -1
##$AQSEQ= 0
```

```
##$AQ_mod= 3
##$AUNM= <au_zg>
##$AUTOPOS= <>
##$BF1= 500.125
##$BF2= 50.677226
##$BF3= 500.125
##$BF4= 500.125
##$BF5= 500.125
##$BF6= 500.125
##$BF7= 500.125
##$BF8= 500.125
##$BYTORDA= 1
##$CFDGTYP= 2
##$CFRGTYP= 5
##$CHEMSTR= <none>
##$CNST= (0..63)
1 0.4868 145 1 92 1 1 1 1 1 1 1 1544.75 1 1 1 1 1 1 1 1 1 1 1 1 1
1 1 1
1 1 1 1 1 1 1 1 1 1 1 1 1 1 1 1 1 1 1 1 1 1 1 1 1 1 1 1 1 1 1 1 1
1 1
##$CPDPRG= <>
##$CPDPRG1= <>
##$CPDPRG2= <garp>
##$CPDPRG3= <garp>
##$CPDPRG4= <mlev>
##$CPDPRG5= <mlev>
##$CPDPRG6= <mlev>
##$CPDPRG7= <mlev>
##$CPDPRG8= <mlev>
##$CPDPRGB= <>
##$CPDPRGT= <>
##$D= (0..63)
3e-06 2 0.002717391 0 0.001412391 1.239134e-05 0 0 0 0.06 2.346e-05
0 0
0.018179 0.075508 5e-06 0.0003 0 0 0 0.05 0 0.002729121 0.002702661
0.00277778
0 0 0 0 0 0.03 0 0 0 0 0 0 0 0 0 0 0 0 0 0 0 0 0 0 0 0 0 0 0 0 0
0 0 0
0 0 0 0 0
##$DATE= 1417893593
##$DBL= (0..7)
120 120 120 120 120 120 120 120
##$DBP= (0..7)
150 150 150 150 150 150 150 150
```

```
##$DBP07= 0
##$DBPNAM0= <>
##$DBPNAM1= <>
##$DBPNAM2= <>
##$DBPNAM3= <>
##$DBPNAM4= <>
##$DBPNAM5= <>
##$DBPNAM6= <>
##$DBPNAM7= <>
##$DBPOAL= (0..7)
0.5 0.5 0.5 0.5 0.5 0.5 0.5 0.5
##$DBPOFFS= (0..7)
0 0 0 0 0 0 0 0
##$DE= 6.5
##$DECBNUC= <off>
##$DECIM= 24
##$DECNUC= <off>
##$DECSTAT= 4
##$DIGMOD= 1
##$DIGTYP= 8
##$DL= (0..7)
0 120 120 120 120 120 120 120
##$DP= (0..7)
150 150 150 150 150 150 150 150
##$DP07= 0
##$DPNAME0= <>
##$DPNAME1= <>
##$DPNAME2= <>
##$DPNAME3= <>
##$DPNAME4= <>
##$DPNAME5= <>
##$DPNAME6= <>
##$DPNAME7= <>
##$DPOAL= (0..7)
0.5 0.5 0.5 0.5 0.5 0.5 0.5 0.5
##$DPOFFS= (0..7)
0 0 0 0 0 0 0 0
##$DQDMODE= 0
##$DR= 18
##$DS= 256
##$DSLIST= <SSSSSSSSSSSSSSS>
##$DSPFIRM= 0
##$DSPFVS= 12
##$DTYPA= 0
```

```
##$EXP= <>
##$F1LIST= <111111111111111>
##$F2LIST= <222222222222222>
##$F3LIST= <333333333333333>
##$FCUCHAN= (0..9)
0 2 1 0 0 0 0 0 0 0
##$FL1= 90
##$FL2= 90
##$FL3= 90
##$FL4= 90
##$FOV= 20
##$FQ1LIST= <freqlist>
##$FQ2LIST= <freqlist>
##$FQ3LIST= <freqlist>
##$FQ4LIST= <freqlist>
##$FQ5LIST= <freqlist>
##$FQ6LIST= <freqlist>
##$FQ7LIST= <freqlist>
##$FQ8LIST= <freqlist>
##$FRQLO3= 1885504.20168067
##$FRQLO3N= 0
##$FS= (0..7)
83 83 83 83 83 83 83 83
##$FTLPGN= 0
##$FW= 125000
##$FnMODE= 0
##$FnTYPE= 0
##$GP031= 0
##$GPNAM0= <SINE.100>
##$GPNAM1= <SINE.100>
##$GPNAM10= <sine.100>
##$GPNAM11= <sine.100>
##$GPNAM12= <sine.100>
##$GPNAM13= <sine.100>
##$GPNAM14= <sine.100>
##$GPNAM15= <sine.100>
##$GPNAM16= <sine.100>
##$GPNAM17= <sine.100>
##$GPNAM18= <sine.100>
##$GPNAM19= <sine.100>
##$GPNAM2= <SINE.100>
##$GPNAM20= <sine.100>
##$GPNAM21= <sine.100>
##$GPNAM22= <sine.100>
```

##$GPNAM23= <sine.100>
##$GPNAM24= <sine.100>
##$GPNAM25= <sine.100>
##$GPNAM26= <sine.100>
##$GPNAM27= <sine.100>
##$GPNAM28= <sine.100>
##$GPNAM29= <sine.100>
##$GPNAM3= <SINE.100>
##$GPNAM30= <sine.100>
##$GPNAM31= <sine.100>
##$GPNAM4= <SINE.100>
##$GPNAM5= <SINE.100>
##$GPNAM6= <SINE.100>
##$GPNAM7= <SINE.100>
##$GPNAM8= <SINE.100>
##$GPNAM9= <SINE.100>
##$GPX= (0..31)
0 0 0 0 0 0 0 0 0 0 0 0 0 0 0 0 0 0 0 0 0 0 0 0 0 0 0 0 0 0 0 0
##$GPY= (0..31)
0 0 0 0 0 0 0 0 0 0 0 0 0 0 0 0 0 0 0 0 0 0 0 0 0 0 0 0 0 0 0 0
##$GPZ= (0..31)
13 7 43 29 71 23 0 31 0 0 0 0 0 0 0 0 0 0 0 0 0 0 0 0 0 0 0 0 0 0
0 0
##$GRDPROG= <grad_out>
##$GRPDLY= -1
##$HDDUTY= 20
##$HDRATE= 20
##$HGAIN= (0..3)
0 0 0 0
##$HL1= 256
##$HL2= 35
##$HL3= 16
##$HL4= 17
##$HOLDER= 0
##$HPMOD= (0..7)
0 0 0 0 0 0 0 0
##$HPPRGN= 0
##$IN= (0..63)
0.0004484 0.001 0.001 0.001 0.001 0.001 0.001 0.001 0.001 0.001 0.001
0.001
0.001 0.001 0.001 0.001 0.001 0.001 0.001 0.001 0.001 0.001 0.001
0.001
0.001 0.001 0.001 0.001 0.001 0.001 0.001 0.001 0.001 0.001 0.001
0.001

0.001 0.001 0.001 0.001 0.001 0.001 0.001 0.001 0.001 0.001 0.001
0.001
0.001 0.001 0.001 0.001 0.001 0.001 0.001 0.001 0.001 0.001 0.001
0.001
0.001 0.001 0.001 0.001
##$INF= (0..7)
0 896.836251561966 0 0 0 0 0 0
##$INP= (0..63)
0 0 0 0 0 0 0 0 0 0 0 0 0 0 0 0 0 0 0 0 0 0 0 0 0 0 0 0 0 0 0 0 0
0 0 0
0 0 0 0 0 0 0 0 0 0 0 0 0 0 0 0 0 0 0 0 0 0 0 0 0 0 0 0
##$INSTRUM= <spect>
##$L= (0..31)
1 1 1 1 1 1 1 1 1 1 1 1 1 1 1 1 1 1 1 1 1 1 1 1 1 1 1 1 1 1 1 1
##$LFILTER= 57
##$LGAIN= -7.69999980926514
##$LINPSTP= 10
##$LOCKED= yes
##$LOCKFLD= 4357
##$LOCKGN= 108.5
##$LOCKPOW= -18
##$LOCKPPM= 4.69999980926514
##$LOCNUC= <2H>
##$LOCPHAS= 253.7
##$LOCSHFT= no
##$LOCSW= 0
##$LTIME= 0.419999986886978
##$MASR= 4200
##$MASRLST= <masrlst>
##$NBL= 1
##$NC= -2
##$NLOGCH= 4
##$NS= 8
##$NUC1= <1H>
##$NUC2= <15N>
##$NUC3= <off>
##$NUC4= <off>
##$NUC5= <off>
##$NUC6= <off>
##$NUC7= <off>
##$NUC8= <off>
##$NUCLEI= 0
##$NUCLEUS= <off>
##$O1= 2318.1

```
##$O2= 5990
##$O3= 59114.775
##$O4= 0
##$O5= 2375.4762511885
##$O6= 2375.4762511885
##$O7= 2375.4762511885
##$O8= 2375.4762511885
##$OBSCHAN= (0..9)
0 0 0 0 0 0 0 0 0 0
##$OVERFLW= 1
##$P= (0..63)
8.3 8.73 45 12.4 24.8 16.5 25 50 0 15000 80000 1400 2240 2240 500
200000
1000 1000 0 0 1400 54.5 70 0 0 100 0 8.3 0 0 0 0 0 0 0 0 0 0 0 0
0 0 0
0 0 0 0 0 0 0 0 0 0 0 0 0 0 0 0 0 0 0 0 1500
##$PACOIL= (0..15)
0 0 0 0 0 0 0 0 0 0 0 0 0 0 0 0
##$PAPS= 2
##$PARMODE= 1
##$PCPD= (0..9)
100 55 320 320 100 100 100 100 100 100
##$PHCOR= (0..31)
0 0 0 0 0 0 0 0 0 0 0 0 0 0 0 0 0 0 0 0 0 0 0 0 0 0 0 0 0 0 0 0
##$PHLIST= <>
##$PHP= 1
##$PH_ref= 0
##$PL= (0..63)
120 4.65 -1 -1 120 120 120 120 120 52.52 10.58 22.62 9.6 120 120
120 16.04
120 1 17.43 120 120 120 120 120 120 120 22.62 120 120 120 120 120
120 120
120 120 120 120 120 120 120 120 120 120 120 120 120 120 120 120 120
120
120 120 120 120 120 120 120 120 120 120 120
##$PLSTEP= 0.1
##$PLSTRT= -6
##$POWMOD= 0
##$PQPHASE= 0
##$PQSCALE= 0
##$PR= 1
##$PRECHAN= (0..15)
-1 3 0 4 -1 -1 2 -1 -1 -1 -1 -1 -1 -1 -1 -1
##$PRGAIN= 0
```

```
##$PROBHD= <5 mm CPTCI 1H-13C/15N/D Z-GRD Z108549/0001
>
##$PROSOL= no
##$PULPROG= <fab_nnh_noSE>
##$PW= 0
##$PYNM= <acqu.py>
##$QNP= 1
##$RD= 0
##$RECCHAN= (0..15)
0 2 0 0 0 0 0 0 0 0 0 0 0 0 0 0
##$RECPH= 0
##$RECPRE= (0..15)
-1 0 -1 -1 -1 -1 -1 -1 -1 -1 -1 -1 -1 -1 -1 -1
##$RECPRFX= (0..15)
-1 0 0 0 0 0 0 0 0 0 0 0 0 0 0 0
##$RECSEL= (0..15)
0 0 1 0 0 0 0 0 0 0 0 0 0 0 0 0
##$RG= 512
##$RO= 0
##$ROUTWD1= (0..23)
0 1024 0 0 0 0 0 0 0 0 0 0 0 0 0 0 0 0 0 0 1 1 0 0
##$ROUTWD2= (0..23)
0 0 0 0 0 1 0 0 0 0 0 0 0 0 0 0 0 0 1 0 1 1 0 0
##$RSEL= (0..9)
0 3 2 0 0 0 0 0 0 0
##$S= (0..7)
83 4 83 83 83 83 83 83
##$SEOUT= 0
##$SFO1= 500.1273181
##$SFO2= 50.683216
##$SFO3= 500.184114775
##$SFO4= 500.125
##$SFO5= 500.127375476251
##$SFO6= 500.127375476251
##$SFO7= 500.127375476251
##$SFO8= 500.127375476251
##$SOLVENT= <H2O+D2O>
##$SP= (0..31)
1 45.5 120 1 0 0 120 120 0 7.75 7.75 0 46 55 0 0 150 150 150 150
150 150
150 150 150 150 150 150 150 150 150 150
##$SP07= 0
##$SPECTR= 0
##$SPNAM0= <gauss>
```

```
##$SPNAM1= <Sinc1.1000>
##$SPNAM10= <SmoothedChirp_80ms_10kHz_20per_10000pt:>
##$SPNAM11= <gauss>
##$SPNAM12= <Sinc1.1000>
##$SPNAM13= <Sinc1.1000>
##$SPNAM14= <gauss>
##$SPNAM15= <gauss>
##$SPNAM16= <gauss>
##$SPNAM17= <gauss>
##$SPNAM18= <gauss>
##$SPNAM19= <gauss>
##$SPNAM2= <Gaus1.1000>
##$SPNAM20= <gauss>
##$SPNAM21= <gauss>
##$SPNAM22= <gauss>
##$SPNAM23= <gauss>
##$SPNAM24= <gauss>
##$SPNAM25= <gauss>
##$SPNAM26= <gauss>
##$SPNAM27= <gauss>
##$SPNAM28= <gauss>
##$SPNAM29= <gauss>
##$SPNAM3= <Crp60,0.5,20.1>
##$SPNAM30= <gauss>
##$SPNAM31= <gauss>
##$SPNAM4= <gauss>
##$SPNAM5= <gauss>
##$SPNAM6= <Gaus1.1000>
##$SPNAM7= <Gaus1.1000>
##$SPNAM8= <gauss>
##$SPNAM9= <SmoothedChirp_15ms_10kHz_20per_1000pt>
##$SPOAL= (0..31)
0.5 0.5 0.5 0.5 0.5 0.5 0.5 0.5 0.5 0.5 0.5 0.5 0.5 0.5 0.5 0.5 0.5
0.5
0.5 0.5 0.5 0.5 0.5 0.5 0.5 0.5 0.5 0.5 0.5 0.5 0.5 0.5
##$SPOFFS= (0..31)
0 0 0 0 0 0 0 0 0 0 0 0 -1544.75 -1544.75 0 0 0 0 0 0 0 0 0 0 0 0
0 0 0
0 0 0
##$SUBNAM0= <"">
##$SUBNAM1= <"">
##$SUBNAM2= <"">
##$SUBNAM3= <"">
##$SUBNAM4= <"">
```

```
##$SUBNAM5= <"">
##$SUBNAM6= <"">
##$SUBNAM7= <"">
##$SUBNAM8= <"">
##$SUBNAM9= <"">
##$SW= 14.0020368154494
##$SWIBOX= (0..15)
0 1 2 3 0 0 6 0 0 0 0 0 0 0 0 0
##$SW_h= 7002.80112044818
##$SWfinal= 0
##$TD= 2048
##$TD0= 1
##$TE= 307.6
##$TE2= 300
##$TE3= 300
##$TEG= 300
##$TL= (0..7)
0 120 120 120 120 120 120 120
##$TP= (0..7)
150 150 150 150 150 150 150 150
##$TP07= 0
##$TPNAME0= <>
##$TPNAME1= <>
##$TPNAME2= <>
##$TPNAME3= <>
##$TPNAME4= <>
##$TPNAME5= <>
##$TPNAME6= <>
##$TPNAME7= <>
##$TPOAL= (0..7)
0.5 0.5 0.5 0.5 0.5 0.5 0.5 0.5
##$TPOFFS= (0..7)
0 0 0 0 0 0 0 0
##$TUNHIN= 0
##$TUNHOUT= 0
##$TUNXOUT= 0
##$USERA1= <user>
##$USERA2= <user>
##$USERA3= <user>
##$USERA4= <user>
##$USERA5= <user>
##$V9= 5
##$VALIST= <valist>
##$VCLIST= <CCCCCCCCCCCCCCC>
```

```
##$VD= 0
##$VDLIST= <DDDDDDDDDDDDDDDD>
##$VPLIST= <PPPPPPPPPPPPPPPP>
##$VTLIST= <TTTTTTTTTTTTTTTT>
##$WBST= 1024
##$WBSW= 0.6
##$XGAIN= (0..3)
0 0 0 0
##$XL= 0
##$YL= 0
##$YMAX_a= 55520
##$YMIN_a= -52830
##$ZGOPTNS= <>
##$ZL1= 120
##$ZL2= 120
##$ZL3= 120
##$ZL4= 120
##END=
```

Bruker pulse program for the measurement of spectral density values at zero frequency $J(0)$ using a single echo experiment (scheme 2 in Fig. 1):

```
#include <Avance.incl>
#include <Grad.incl>



"d16=300u"
"d15=5u"
"d2 = 1/(4*cnst4)"
"d4 = d2-p16-d16-d15"
"d5 = d2-p16-d16-d15-p11"
"d30 = 30m"
"d0 = 3u"
"d10 = p1*2+d0*2"
"d22 = d2+p1+d0"
"d23 = d2-p1-d0-3u"
"d24 = d20*0.5-p12-6u"
"d13 = d20*0.25-p12-126u"


;"l5=1"
"in0=inf1/2"

1 ze
2 d30 do:f2
3 d1
10u pl0:f2
(p10:sp10):f2
10u
10u pl1:f1 pl2:f2
(p2 ph0):f2
d15 UNBLKGRAD
p16:gp0
1m
(p1 ph0):f1
d15
p16:gp1
d16
d4
(center (p1*2 ph2):f1 (p2*2 ph0):f2)
d4
```

```
d15
p16:gp1
d16
(p1 ph4):f1
d15
p20:gp2
1m

if "l5 if "l5
(p2 ph10):f2
d2
(center (p1*2 ph0):f1 (p2*2 ph0):f2)
d2
(p2 ph3):f2
d15
p16:gp5
d16
(p1 ph0):f1
1u

goto 30

20 d15
p16:gp5
d16
(p1 ph0):f1
3u
(p1 ph10):f1
1u

;******* relaxation *******



30 20u
d13 pl0:f1
100u fq=cnst12:f1
(p12:sp12 ph21):f1
3u
3u pl1:f1
(center (p1 ph0 3u p1*2 ph1 3u p1 ph0):f1 (p2 ph11):f2)

3u
3u pl0:f1
```

```
(p13:sp13 ph22):f1

d24
(p2*2 ph0):f2
d24

(p12:sp12 ph21):f1
3u
3u pl1:f1

(center (p1 ph0 3u p1*2 ph1 3u p1 ph0):f1 (p2 ph12):f2)
3u
3u pl0:f1
(p13:sp13 ph22):f1
20u
d13 pl1:f1 pl2:f2
100u fq=0:f1

;******** end relaxation **********

if "l5 if "l5
1u
(p1 ph0):f1
d15
p16:gp7
d16
(p2 ph5):f2
d22
(p2*2 ph24):f2
d0
(p1*2 ph1):f1
d23
d0
(p2 ph3):f2
3u
goto 50

40 1u
(p1 ph0):f1
3u
(p1 ph14):f1
d15
p16:gp7
d16
```

```
(p2 ph5):f2
d0
(p1*2 ph0):f1
d0
(p2*2 ph0):f2
d10
(p2 ph0):f2

50 d15
p16:gp3
d16
(p1 ph0):f1
d15
p16:gp4
d16
d5
3u pl0:f1
3u
(p11:sp1 ph23:r):f1
3u
3u pl1:f1
3u
(center (p1*2 ph1):f1 (p2*2 ph0):f2)
3u
3u pl0:f1
3u
(p11:sp1 ph23:r):f1
6u
d15
p16:gp4
d16 BLKGRAD
d5 pl16:f2
go=2 ph31 cpds2:f2
d30 do:f2 mc #0 to 2
F1I(iu5, 4)
F1PH(ru5 & ip5, id0)
d30 do:f2
exit

ph0=0
ph1=1
ph2=2
ph3=3
ph21=1
```

```
ph22=1
ph23=3
ph4=1*8 3*8
ph5=0
ph10 = 0*4 2*4
ph11 = 0*1 2*1
ph12 = 0*16 2*16
ph14 = 0*2 2*2
ph15 = 2*2 0*2
ph20 = 2*4 0*4
ph24 = 0*2 1*2


ph31=0 2 2 0 2 0 0 2
2 0 0 2 0 2 2 0
2 0 0 2 0 2 2 0
0 2 2 0 2 0 0 2



;pl1 :  f1 channel - power level for pulse (default)
;p1 :  f1 channel - high power pulse
;d1 :  relaxation delay; 1-5 * T1
```

Acquisition parameters for the measurement of spectral density values at zero frequency $J(0)$ using a single echo experiment (scheme 2 in Fig. 1) at 500 MHz and 5°C:

```
##TITLE= Parameter file, TOPSPIN Version 2.1
##JCAMPDX= 5.0
##DATATYPE= Parameter Values
##NPOINTS= 12 $$ modification sequence number
##ORIGIN= Bruker BioSpin GmbH
##OWNER= username
$$ 2015-01-11 06:50:05.870 +0100 username@nmrspectrometer
$$ /opt/data/username/nmrspectrometer/Ubiq_5C/39/acqus
$$ process /opt/topspin/prog/mod/shimcntl
##$ACQT0= 1000000
##$AMP= (0..31)
100 100 100 100 100 100 100 100 100 100 100 100 100 100 100 100 100
100
100 100 100 100 100 100 100 100 100 100 100 100 100 100
##$ANAVPT= -1
```

```
##$AQSEQ= 0
##$AQ_mod= 3
##$AUNM= <au_zg>
##$AUTOPOS= <>
##$BF1= 500.125
##$BF2= 50.677226
##$BF3= 500.125
##$BF4= 500.125
##$BF5= 500.125
##$BF6= 500.125
##$BF7= 500.125
##$BF8= 500.125
##$BYTORDA= 1
##$CFDGTYP= 2
##$CFRGTYP= 5
##$CHEMSTR= <none>
##$CNST= (0..63)
1 1 145 1 92 1 1 1 1 1 1 1 1544.75 1 1 1 1 1 1 1 1 1 1 1 1 1 1 1
1 1 1
1 1 1 1 1 1 1 1 1 1 1 1 1 1 1 1 1 1 1 1 1 1 1 1 1 1 1 1 1 1 1 1 1
##$CPDPRG= <>
##$CPDPRG1= <>
##$CPDPRG2= <garp>
##$CPDPRG3= <garp>
##$CPDPRG4= <mlev>
##$CPDPRG5= <mlev>
##$CPDPRG6= <mlev>
##$CPDPRG7= <mlev>
##$CPDPRG8= <mlev>
##$CPDPRGB= <>
##$CPDPRGT= <>
##$D= (0..63)
3e-06 2 0.002717391 0 0.001412391 1.239134e-05 0 0 0 0.06 2.302e-05
0 0
0.014694 0 5e-06 0.0003 0 0 0 0.07 0 0.002728901 0.002702881 0.032314
0
0 0 0 0 0.03 0 0 0 0 0 0 0 0 0 0 0 0 0 0 0 0 0 0 0 0 0 0 0 0 0 0
0 0 0
0 0 0 0
##$DATE= 1420875278
##$DBL= (0..7)
120 120 120 120 120 120 120 120
##$DBP= (0..7)
150 150 150 150 150 150 150 150
```

```
##$DBP07= 0
##$DBPNAM0= <>
##$DBPNAM1= <>
##$DBPNAM2= <>
##$DBPNAM3= <>
##$DBPNAM4= <>
##$DBPNAM5= <>
##$DBPNAM6= <>
##$DBPNAM7= <>
##$DBPOAL= (0..7)
0.5 0.5 0.5 0.5 0.5 0.5 0.5 0.5
##$DBPOFFS= (0..7)
0 0 0 0 0 0 0 0
##$DE= 6.5
##$DECBNUC= <off>
##$DECIM= 24
##$DECNUC= <off>
##$DECSTAT= 4
##$DIGMOD= 1
##$DIGTYP= 8
##$DL= (0..7)
0 120 120 120 120 120 120 120
##$DP= (0..7)
150 150 150 150 150 150 150 150
##$DP07= 0
##$DPNAME0= <>
##$DPNAME1= <>
##$DPNAME2= <>
##$DPNAME3= <>
##$DPNAME4= <>
##$DPNAME5= <>
##$DPNAME6= <>
##$DPNAME7= <>
##$DPOAL= (0..7)
0.5 0.5 0.5 0.5 0.5 0.5 0.5 0.5
##$DPOFFS= (0..7)
0 0 0 0 0 0 0 0
##$DQDMODE= 0
##$DR= 18
##$DS= 256
##$DSLIST= <SSSSSSSSSSSSSSS>
##$DSPFIRM= 0
##$DSPFVS= 12
##$DTYPA= 0
```

```
##$EXP= <>
##$F1LIST= <111111111111111>
##$F2LIST= <222222222222222>
##$F3LIST= <333333333333333>
##$FCUCHAN= (0..9)
0 2 1 0 0 0 0 0 0 0
##$FL1= 90
##$FL2= 90
##$FL3= 90
##$FL4= 90
##$FOV= 20
##$FQ1LIST= <freqlist>
##$FQ2LIST= <freqlist>
##$FQ3LIST= <freqlist>
##$FQ4LIST= <freqlist>
##$FQ5LIST= <freqlist>
##$FQ6LIST= <freqlist>
##$FQ7LIST= <freqlist>
##$FQ8LIST= <freqlist>
##$FRQLO3= 1885504.20168067
##$FRQLO3N= 0
##$FS= (0..7)
83 83 83 83 83 83 83 83
##$FTLPGN= 0
##$FW= 125000
##$FnMODE= 0
##$FnTYPE= 0
##$GP031= 0
##$GPNAM0= <SINE.100>
##$GPNAM1= <SINE.100>
##$GPNAM10= <sine.100>
##$GPNAM11= <sine.100>
##$GPNAM12= <sine.100>
##$GPNAM13= <sine.100>
##$GPNAM14= <sine.100>
##$GPNAM15= <sine.100>
##$GPNAM16= <sine.100>
##$GPNAM17= <sine.100>
##$GPNAM18= <sine.100>
##$GPNAM19= <sine.100>
##$GPNAM2= <SINE.100>
##$GPNAM20= <sine.100>
##$GPNAM21= <sine.100>
##$GPNAM22= <sine.100>
```

```
##$GPNAM23= <sine.100>
##$GPNAM24= <sine.100>
##$GPNAM25= <sine.100>
##$GPNAM26= <sine.100>
##$GPNAM27= <sine.100>
##$GPNAM28= <sine.100>
##$GPNAM29= <sine.100>
##$GPNAM3= <SINE.100>
##$GPNAM30= <sine.100>
##$GPNAM31= <sine.100>
##$GPNAM4= <SINE.100>
##$GPNAM5= <SINE.100>
##$GPNAM6= <SINE.100>
##$GPNAM7= <SINE.100>
##$GPNAM8= <SINE.100>
##$GPNAM9= <SINE.100>
##$GPX= (0..31)
0 0 0 0 0 0 0 0 0 0 0 0 0 0 0 0 0 0 0 0 0 0 0 0 0 0 0 0 0 0 0 0
##$GPY= (0..31)
0 0 0 0 0 0 0 0 0 0 0 0 0 0 0 0 0 0 0 0 0 0 0 0 0 0 0 0 0 0 0 0
##$GPZ= (0..31)
13 7 43 29 71 23 0 31 0 0 0 0 0 0 0 0 0 0 0 0 0 0 0 0 0 0 0 0 0 0
0 0
##$GRDPROG= <grad_out>
##$GRPDLY= -1
##$HDDUTY= 20
##$HDRATE= 20
##$HGAIN= (0..3)
0 0 0 0
##$HL1= 256
##$HL2= 35
##$HL3= 16
##$HL4= 17
##$HOLDER= 0
##$HPMOD= (0..7)
0 0 0 0 0 0 0 0
##$HPPRGN= 0
##$IN= (0..63)
0.0004484 0.001 0.001 0.001 0.001 0.001 0.001 0.001 0.001 0.001 0.001
0.001
0.001 0.001 0.001 0.001 0.001 0.001 0.001 0.001 0.001 0.001 0.001
0.001
0.001 0.001 0.001 0.001 0.001 0.001 0.001 0.001 0.001 0.001 0.001
0.001
```

```
0.001 0.001 0.001 0.001 0.001 0.001 0.001 0.001 0.001 0.001 0.001
0.001
0.001 0.001 0.001 0.001 0.001 0.001 0.001 0.001 0.001 0.001 0.001
0.001
0.001 0.001 0.001 0.001
##$INF= (0..7)
0 896.836251561966 0 0 0 0 0 0
##$INP= (0..63)
0 0 0 0 0 0 0 0 0 0 0 0 0 0 0 0 0 0 0 0 0 0 0 0 0 0 0 0 0 0 0 0 0
0 0 0
0 0 0 0 0 0 0 0 0 0 0 0 0 0 0 0 0 0 0 0 0 0 0 0 0 0 0 0
##$INSTRUM= <spect>
##$L= (0..31)
1 1 1 1 1 1 1 1 1 1 1 1 1 1 1 1 1 1 1 1 1 1 1 1 1 1 1 1 1 1 1 1
##$LFILTER= 40
##$LGAIN= -11
##$LINPSTP= 10
##$LOCKED= yes
##$LOCKFLD= 4374
##$LOCKGN= 112
##$LOCKPOW= -18
##$LOCKPPM= 4.69999980926514
##$LOCNUC= <2H>
##$LOCPHAS= 258.7
##$LOCSHFT= no
##$LOCSW= 0
##$LTIME= 0.503000020980835
##$MASR= 4200
##$MASRLST= <masrlst>
##$NBL= 1
##$NC= -2
##$NLOGCH= 4
##$NS= 32
##$NUC1= <1H>
##$NUC2= <15N>
##$NUC3= <off>
##$NUC4= <off>
##$NUC5= <off>
##$NUC6= <off>
##$NUC7= <off>
##$NUC8= <off>
##$NUCLEI= 0
##$NUCLEUS= <off>
##$O1= 2313.57825
```

```
##$O2= 5990
##$O3= 59114.775
##$O4= 0
##$O5= 2375.4762511885
##$O6= 2375.4762511885
##$O7= 2375.4762511885
##$O8= 2375.4762511885
##$OBSCHAN= (0..9)
0 0 0 0 0 0 0 0 0 0
##$OVERFLW= 1
##$P= (0..63)
8.3 8.51 44.5 12.4 24.8 16.5 25 50 0 0 80000 1400 2680 2680 500 200000
1000 0 0 0 1400 54.5 70 0 0 100 0 8.3 0 0 0 0 0 0 0 0 0 0 0 0 0 0
0 0 0
0 0 0 0 0 0 0 0 0 0 0 0 0 0 0 0 0 0 1500
##$PACOIL= (0..15)
0 0 0 0 0 0 0 0 0 0 0 0 0 0 0 0
##$PAPS= 2
##$PARMODE= 1
##$PCPD= (0..9)
100 55 320 320 100 100 100 100 100 100
##$PHCOR= (0..31)
0 0 0 0 0 0 0 0 0 0 0 0 0 0 0 0 0 0 0 0 0 0 0 0 0 0 0 0 0 0 0 0
##$PHLIST= <>
##$PHP= 1
##$PH_ref= 0
##$PL= (0..63)
120 4.65 -1 -1 120 120 120 120 120 52.52 10.58 22.62 9.6 120 120
120 16.14
120 1 17.43 120 120 120 120 120 120 120 22.62 120 120 120 120 120
120 120
120 120 120 120 120 120 120 120 120 120 120 120 120 120 120 120 120
120
120 120 120 120 120 120 120 120 120 120 120
##$PLSTEP= 0.1
##$PLSTRT= -6
##$POWMOD= 0
##$PQPHASE= 0
##$PQSCALE= 0
##$PR= 1
##$PRECHAN= (0..15)
-1 3 0 4 -1 -1 2 -1 -1 -1 -1 -1 -1 -1 -1 -1
##$PRGAIN= 0
##$PROBHD= <5 mm CPTCI 1H-13C/15N/D Z-GRD Z108549/0001
```

```
>
##$PROSOL= no
##$PULPROG= <fab_nnh_noChirp_noSE>
##$PW= 0
##$PYNM= <acqu.py>
##$QNP= 1
##$RD= 0
##$RECCHAN= (0..15)
0 2 0 0 0 0 0 0 0 0 0 0 0 0 0 0
##$RECPH= 0
##$RECPRE= (0..15)
-1 0 -1 -1 -1 -1 -1 -1 -1 -1 -1 -1 -1 -1 -1 -1
##$RECPRFX= (0..15)
-1 0 0 0 0 0 0 0 0 0 0 0 0 0 0 0
##$RECSEL= (0..15)
0 0 1 0 0 0 0 0 0 0 0 0 0 0 0 0
##$RG= 128
##$RO= 0
##$ROUTWD1= (0..23)
0 1024 0 0 0 0 0 0 0 0 0 0 0 0 0 0 0 0 0 0 1 1 0 0
##$ROUTWD2= (0..23)
0 0 0 0 0 1 0 0 0 0 0 0 0 0 0 0 0 0 1 0 1 1 0 0
##$RSEL= (0..9)
0 3 2 0 0 0 0 0 0 0
##$S= (0..7)
83 4 83 83 83 83 83 83
##$SEOUT= 0
##$SFO1= 500.12731357825
##$SFO2= 50.683216
##$SFO3= 500.184114775
##$SFO4= 500.125
##$SFO5= 500.127375476251
##$SFO6= 500.127375476251
##$SFO7= 500.127375476251
##$SFO8= 500.127375476251
##$SOLVENT= <H2O+D2O>
##$SP= (0..31)
1 45 120 1 0 0 120 120 0 0 7.75 0 47 58.4 0 0 150 150 150 150 150
150 150
150 150 150 150 150 150 150 150 150
##$SP07= 0
##$SPECTR= 0
##$SPNAM0= <gauss>
##$SPNAM1= <Sinc1.1000>
```

```
##$SPNAM10= <SmoothedChirp_80ms_10kHz_5per_10000pt>
##$SPNAM11= <gauss>
##$SPNAM12= <Sinc1.1000>
##$SPNAM13= <Sinc1.1000>
##$SPNAM14= <gauss>
##$SPNAM15= <gauss>
##$SPNAM16= <gauss>
##$SPNAM17= <gauss>
##$SPNAM18= <gauss>
##$SPNAM19= <gauss>
##$SPNAM2= <Gaus1.1000>
##$SPNAM20= <gauss>
##$SPNAM21= <gauss>
##$SPNAM22= <gauss>
##$SPNAM23= <gauss>
##$SPNAM24= <gauss>
##$SPNAM25= <gauss>
##$SPNAM26= <gauss>
##$SPNAM27= <gauss>
##$SPNAM28= <gauss>
##$SPNAM29= <gauss>
##$SPNAM3= <Crp60,0.5,20.1>
##$SPNAM30= <gauss>
##$SPNAM31= <gauss>
##$SPNAM4= <gauss>
##$SPNAM5= <gauss>
##$SPNAM6= <Gaus1.1000>
##$SPNAM7= <Gaus1.1000>
##$SPNAM8= <gauss>
##$SPNAM9= <gauss>
##$SPOAL= (0..31)
0.5 0.5 0.5 0.5 0.5 0.5 0.5 0.5 0.5 0.5 0.5 0.5 0.5 0.5 0.5 0.5 0.5
0.5
0.5 0.5 0.5 0.5 0.5 0.5 0.5 0.5 0.5 0.5 0.5 0.5 0.5 0.5
##$SPOFFS= (0..31)
0 0 0 0 0 0 0 0 0 0 0 0 -1544.75 -1544.75 0 0 0 0 0 0 0 0 0 0 0 0
0 0 0
0 0 0
##$SUBNAM0= <"">
##$SUBNAM1= <"">
##$SUBNAM2= <"">
##$SUBNAM3= <"">
##$SUBNAM4= <"">
##$SUBNAM5= <"">
```

```
##$SUBNAM6= <"">
##$SUBNAM7= <"">
##$SUBNAM8= <"">
##$SUBNAM9= <"">
##$SW= 14.0020369420446
##$SWIBOX= (0..15)
0 1 2 3 0 0 6 0 0 0 0 0 0 0 0 0
##$SW_h= 7002.80112044818
##$SWfinal= 0
##$TD= 2048
##$TD0= 1
##$TE= 283.3
##$TE2= 300
##$TE3= 300
##$TEG= 300
##$TL= (0..7)
0 120 120 120 120 120 120 120
##$TP= (0..7)
150 150 150 150 150 150 150 150
##$TP07= 0
##$TPNAME0= <>
##$TPNAME1= <>
##$TPNAME2= <>
##$TPNAME3= <>
##$TPNAME4= <>
##$TPNAME5= <>
##$TPNAME6= <>
##$TPNAME7= <>
##$TPOAL= (0..7)
0.5 0.5 0.5 0.5 0.5 0.5 0.5 0.5
##$TPOFFS= (0..7)
0 0 0 0 0 0 0 0
##$TUNHIN= 0
##$TUNHOUT= 0
##$TUNXOUT= 0
##$USERA1= <user>
##$USERA2= <user>
##$USERA3= <user>
##$USERA4= <user>
##$USERA5= <user>
##$V9= 5
##$VALIST= <valist>
##$VCLIST= <CCCCCCCCCCCCCCC>
##$VD= 0
```

```
##$VDLIST= <DDDDDDDDDDDDDDDD>
##$VPLIST= <PPPPPPPPPPPPPPPP>
##$VTLIST= <TTTTTTTTTTTTTTTT>
##$WBST= 1024
##$WBSW= 0.6
##$XGAIN= (0..3)
0 0 0 0
##$XL= 0
##$YL= 0
##$YMAX_a= 57067
##$YMIN_a= -35787
##$ZGOPTNS= <>
##$ZL1= 120
##$ZL2= 120
##$ZL3= 120
##$ZL4= 120
##END=
```

References

[Hogben et al(2011)] Hogben HJ, Krzystyniak M, Charnock GTP, Hore PJ, Kuprov I (2011) Spinach A software library for simulation of spin dynamics in large spin systems. J Magn Reson 208:179–194, DOI 10.1016/j.jmr.2010.11.008

[MATLAB] MATLAB and Statistics Toolbox Release R2015a, The MathWorks, Inc, Natick, Massachusetts, United States

[Redfield(1965)] Redfield AG (1965) The theory of relaxation processes. Adv Magn Reson 1:1–32

[Wangsness and Bloch(1953)] Wangsness R, Bloch F (1953) The Dynamical Theory of Nuclear Induction. Phys Rev Lett 89:728–739, DOI 10.1103/PhysRev.89.728
